# Dyadic Partition-Based Training Schemes for TV/TGV Denoising

**DOI:** 10.1007/s10851-024-01213-x

**Published:** 2024-10-23

**Authors:** Elisa Davoli, Rita Ferreira, Irene Fonseca, José A. Iglesias

**Affiliations:** 1 Institute of Analysis and Scientific Computing, TU Wien, Wiedner Hauptstrasse 8-10, 1040 Vienna, Austria; 2 CEMSE Division, King Abdullah University of Science and Technology (KAUST), Thuwal, 23955-6900 Saudi Arabia; 3 Carnegie Mellon University, 5000 Forbes Avenue, Pittsburgh, PA 15213 USA; 4 Department of Applied Mathematics, University of Twente, P.O. Box 217, 7500 AE Enschede, The Netherlands

**Keywords:** Total variation, Total generalized variation, Discontinuous weights, Spatially-dependent regularization parameters, Box constraint, Bilevel optimization, 68U10, 26B30, 49J10, 94A08

## Abstract

Due to their ability to handle discontinuous images while having a well-understood behavior, regularizations with total variation (TV) and total generalized variation (TGV) are some of the best-known methods in image denoising. However, like other variational models including a fidelity term, they crucially depend on the choice of their tuning parameters. A remedy is to choose these automatically through multilevel approaches, for example by optimizing performance on noisy/clean image pairs. In this work, we consider such methods with space-dependent parameters which are piecewise constant on dyadic grids, with the grid itself being part of the minimization. We prove existence of minimizers for fixed discontinuous parameters under mild assumptions on the data, which lead to existence of finite optimal partitions. We further establish that these assumptions are equivalent to the commonly used box constraints on the parameters. On the numerical side, we consider a simple subdivision scheme for optimal partitions built on top of any other bilevel optimization method for scalar parameters, and demonstrate its improved performance on some representative test images when compared with constant optimized parameters.

## Introduction

A fundamental problem in image processing is the restoration of a given “noisy” image. Images are often deteriorated due to several factors occurring, for instance, in the process of transmission or acquisition, such as blur caused by motion or a deficient lens adjustment.

A well-established and successful approach for image restoration is hinged on variational PDE methods, where minimizers of certain energy functionals provide the sought “clean” and “sharp” images. In the particular case where the degradation consists of additive noise, these energy functionals usually take the form

1.1 $$\begin{aligned} \begin{aligned} E(u):= \Vert u - u_\eta \Vert ^p_X + R_\alpha (u)\quad \text {for } u\in {\widetilde{X}}, \end{aligned} \end{aligned}$$

where $u_\eta$ represents the given noisy image and $\widetilde{X}$ is the class of possible reconstructions of $u_\eta$. The first term in (1.1), $\Vert u - u_\eta \Vert ^p_X$, is the fidelity or data fitting term that, in a minimization process, controls the distance between $u$ and $u_\eta$ in some space $X$. The second term, $R_\alpha (u)$, is the so-called filter term, and is responsible for the regularization of the images. The parameter $\alpha$ is often called a tuning or regularization parameter, and accounts for a balance between the fidelity and filter terms.

A milestone approach in imaging denoising is due to Rudin, Osher, and Fatemi [60], who proposed (in a discrete setting, later extended to a function space framework in [1, 18]) an energy functional of the type (1.1) with $$X:=L^2(Q)$$, $$p:=2$$, $${\widetilde{X}}:= BV(Q)$$, and $$R_\alpha (u):= \alpha TV(u,Q)$$ with $$\alpha >0$$, where $$Q\subset \mathbb {R}^2$$ is the image domain and $$ TV(u,Q)$$ is the total variation in $$Q$$ of a function of bounded variation $$u\in BV(Q)$$. Precisely, given an observed noisy version $$u_\eta \in L^2(Q) $$ of a true image, the ROF or TV model consists in finding a reconstruction of the original clean image as the solution of the minimization problem

1.2 $$\begin{aligned} \begin{aligned} \min _{u\in BV(Q)} \Big \{\Vert u - u_\eta \Vert ^2_{L^2(Q)} +\alpha TV(u,Q)\Big \}. \end{aligned} \end{aligned}$$

A striking feature of this model is that it removes noise while preserving images’ edges. This model has been extended in several ways, including higher-order and vectorial settings to address color images, and gave rise to numerous related filter terms seeking to overcome some of its drawbacks, such as blurring and the staircasing effect (see, for instance, [4, 10, 22] for an overview).

In a nutshell, the TV model yields functions *u* that best fit the data, measured in terms of the $L^2$ norm, and whose gradient (total variation) is low so that noise is removed. The choice of the parameter $\alpha$ plays a decisive role in the success of this and similar variational approaches, as it balances the fitting and regularization features of such models. In fact, higher values of $\alpha$ in (1.2) lead to an oversmoothed reconstruction of $u_\eta$ because the total variation has to be “small” to compensate for high values of $\alpha$; conversely, lower values of $\alpha$ in (1.2) inhibit noise removal and, in particular, the reconstructed image provided by (1.2) converges to $u_\eta$ as $\alpha \rightarrow 0$ (see [34]).

In principle, the “optimal” parameter $\alpha$ needs to be chosen individually for each noisy image, which makes such models require additional information to be complete. To address this issue, a partial automatic selection of an “optimal” parameter $\alpha$ was proposed in [34, 35] (see also [23, 24, 39, 61]) in the flavor of machine learning optimization schemes. This automatic selection is based on a bilevel optimization scheme searching for the optimal $\alpha$ that minimizes the distance, in some space, between the reconstruction of a noisy image and the original clean image. In this setting, both the noisy image, $u_\eta$, and the original clean image, $u_c$, are known a priori and called the training data. The rationale is to use the same parameter $\alpha$ to reconstruct noisy images that are *qualitatively similar* to that of the training scheme and corrupted by a similar type and amount of noise and are thus expected to require a similar balance between fitting and regularization effects.

In the context of the TV model in (1.2), one such bilevel optimization scheme reads as follows. Here, and in the sequel, $\mathbb {R}^+$ stands for the set of positive real numbers, $(0,\infty )$. Moreover, for minimization problems over $\mathbb {R}^+$ or $\mathbb {R}^+ \times \mathbb {R}^+$, we write ${{\,\textrm{arginf}\,}}$ instead of ${\text {argmin}}$ to include the case where the infimum would be attained at the boundary of these open sets.

1.3 $$\begin{aligned}&({\mathscr {L}}\!{\mathscr {S}})_{TV}\; \mathbf{TV\; learning\; scheme}&\end{aligned}$$

**Level 1.** Find

$$\begin{aligned} \begin{aligned} \bar{\alpha }=\textrm{arg inf}\left\{ \int _Q |u_c-u_\alpha |^2\;\text {d}x:\,\alpha \in \mathbb {R}^+\right\} ; \end{aligned} \end{aligned}$$

**Level 2.** Given $\alpha \in \mathbb {R}^+$, find

$$\begin{aligned} \begin{aligned} u_\alpha = {\text {argmin}}\left\{ \int _{Q}|u_\eta -u|^2\;\text {d}x+\alpha TV(u,Q):\,u\in BV(Q)\right\} . \end{aligned} \end{aligned}$$

This approach yields a unified way of identifying the best fitting parameters for every class of training data lying in the same $L^2$-neighborhood. However, the learning scheme (1.3) does not address a major drawback of the TV and similar models using scalar regularization parameters. In fact, it does not take into account possible inhomogeneous noise (occurring, e.g., in parallel acquisition in magnetic resonance imaging [38]) and other local features in a given deteriorated image that would benefit from an adapted treatment.

A solution to this issue consists in resorting to adaptive methods and varying fitting parameters instead. The mathematical literature in this direction is vast, from which we single out the following contributions: [49, 58] for results in the finite-dimensional case and for optimal image filters, [33] for bilevel learning in function spaces and development of numerical optimization, [29, 30, 51–53] for a study of optimal regularizers, [54] for a bilevel analysis of novel classes of semi-norms, [55] for an approach via Young measures, and [14, 26, 37, 42] and the references therein for an overview.

A relevant question in image reconstruction (as pointed out in [50], among others) is the possibility of adapting the fitting parameters to the specific features of a given class of noisy images by performing, e.g., a stronger regularization in areas which have been highly deteriorated and by tuning down the filtering actions in portions that, instead, have been left unaffected.

Here, starting from the ideas in [50], we propose space-dependent learning schemes that locally search for the optimal level of refinement and the optimal regularization parameters. The optimal level of refinement translates into finding an optimal partition of the noisy image’s domain that takes into account its local features. Precisely, as before, $Q=(0,1)^2$ represents the images’ domain. We say that $\mathscr {L}$ is an admissible partition of $Q$ if it consists of dyadic squares, each of which we often denote by $L$ (see Sect. 2 for a more detailed description of these partitions). Note that an admissible partition might be more or less refined in different parts of the domain. We denote by $\mathscr {P}$ the class of all such admissible partitions $\mathscr {L}$ of $Q$. Finally, let $(u_\eta ,u_c)\in BV(Q)\times BV(Q)$ be a training pair of noisy and clean images. The first space-dependent learning scheme that we propose to restore $u_\eta$, based on the a priori knowledge of $u_c$, is as follows.

1.4 $$\begin{aligned}&({\mathscr {L}}\!{\mathscr {S}})_{{TV\!}_\omega } { \textbf{Weighted}-TV ~ learning~ scheme}&\end{aligned}$$

**Level 3.**: (optimal local training parameter) Fix $\mathscr {L}\in \mathscr {P}$; for each $L\in \mathscr {L}$, find
  1.5 $$\begin{aligned} \alpha _L:=\inf \left\{ {{\,\textrm{arginf}\,}}\left\{ \int _L |u_c- u_{\alpha ,L}|^2\,\;\text {d}x\!:\,\alpha \in \mathbb {R}^+\right\} \right\} ,\nonumber \\ \end{aligned}$$
  where, for $$\alpha \in \mathbb {R}^+$$,
  1.6 $$\begin{aligned} \begin{aligned} u_{\alpha ,L}&:= {\text {argmin}}\left\{ \int _{L}|u_\eta -u|^2\;\text {d}x +\alpha TV(u,L)\right. \\  &\left. :\,u\in BV(L)\right\} . \end{aligned} \end{aligned}$$
**Level 2.**: (space-dependent image denoising) For each $\mathscr {L}\in$
$$\mathscr {P}$$, find
  1.7 $$\begin{aligned} u_{\mathscr {L}}  &   :={\text {argmin}}\bigg \{\int _Q|u_\eta -u|^2\;\text {d}x +TV_{\omega _\mathscr {L}}(u,Q) \nonumber \\  &   :\,u\in BV_{\omega _{\mathscr {L}}}(Q)\bigg \}, \end{aligned}$$
  where we consider the piecewise constant weight $\omega _{\mathscr {L}}$ defined by
  1.8 $$\begin{aligned} \omega _{\mathscr {L}}(x):=\sum _{L\in \mathscr {L} }\alpha _L \chi _L(x) \quad \text {with }\alpha _L\text { given by Level 3},\nonumber \\ \end{aligned}$$
  and $BV_{\omega _{\mathscr {L}}}$ is the space of $\omega _{\mathscr {L}}$-weighted *BV*-functions (see Sect. 3.2).
**Level 1.**: (optimal partition and image restoration) Find
  $$\begin{aligned}  &   u^*\in {\text {argmin}}\left\{ \int _Q|u_c-u_{\mathscr {L}}|^2\;\text {d}x:\,\mathscr {L}\in \mathscr {P}\right\} \\  &   \text {with}\,\, u_{\mathscr {L}}\text { given by Level 2}. \end{aligned}$$

### Remark 1.1

**(i)** We observe that by taking the infimum in (1.5), the corresponding parameter $\alpha _L$ is always well defined. On the other hand, if $TV(u_\eta ,L) >\ TV(u_c,L)$ and $\Vert u_\eta - u_c\Vert ^2_{L^2(L)} <\Vert [ u_\eta ]_L - u_c\Vert ^2_{L^2(L)}$ as in [34], with $[ u_\eta ]_L:=\frac{1}{|L|}\int _L u_\eta \;\text {d}x$, we prove in Theorem 3.8 that there exists $\tilde{\alpha }_L\in (0,\infty )$ satisfying

$$\begin{aligned} \tilde{\alpha }_L\in {\text {argmin}}\left\{ \int _L |u_c- u_{\alpha ,L}|^2\,\;\text {d}x\!:\,\alpha \in \mathbb {R}^+\right\} \end{aligned}$$

(see [34] for similar statements), in which case the infimum on such $\tilde{\alpha }_L$ as in (1.5) may be regarded as a choice criterium on the optimal parameter.

**(ii)** We refer to Sect. 3.2 for the definition and discussion of the space $BV_{\omega _{\mathscr {L}}}$ of $\omega _{\mathscr {L}}$-weighted *BV*-functions, as introduced in [5]. In particular, using the results in [5] (and also [15, 16]), we prove under appropriate conditions that ${u}_{\mathscr {L}} \in BV(Q)$ and

1.9 $$\begin{aligned} \begin{aligned} TV_{\omega _\mathscr {L}}({u}_{\mathscr {L}},Q) = \int _Q \omega _{\mathscr {L}}^{sc^-}\!(x) \;\text {d}|D{u}_{\mathscr {L}}|(x), \end{aligned} \end{aligned}$$

where $\omega _\mathscr {L}^{sc^-}$ denotes the lower-semicontinuous envelope of $\omega _\mathscr {L}$. We further mention the works in [3, 41] addressing the study of inverse problems that include a weighted-$TV$ model of the form of the one in (1.7).

The existence of solutions to the learning scheme $({\mathscr {L}}\!{\mathscr {S}})_{{TV\!}_\omega }$ in (1.4) is intimately related to the existence of a stopping criterion for the refinement of the admissible partitions or, in other words, a lower bound on the size of the dyadic squares $L\in \mathscr {L}$, with $\mathscr {L}\in \mathscr {P}$. This notion is made precise in the following definition.

### Definition 1.2

(stopping criterion for the refinement of the admissible partitions) We say that a condition $(\mathscr {S})$ on $\mathscr {P}$ is a stopping criterion for the refinement of the admissible partitions if there exist $\kappa \in \mathbb {N}$ and $\mathscr {L}_1,..., \mathscr {L}_\kappa \in \mathscr {P}$ such that

$$\begin{aligned} \begin{aligned}&{\text {argmin}}\left\{ \int _Q|u_c-u_{\mathscr {L}}|^2\;\text {d}x:\,\mathscr {L}\in \mathscr {P}\right\} \\&\quad = {\text {argmin}}\left\{ \int _Q|u_c-u_{\mathscr {L}_i}|^2\;\text {d}x:\,i\in \{1,...,\kappa \}\right\} \end{aligned} \end{aligned}$$

provided that $(\mathscr {S})$ holds, where $u_{\mathscr {L}}$ and $u_{\mathscr {L}_i}$ are given by (1.7). In this case, we write .

We refer to Sect. 3.4 for examples of stopping criteria as in Definition 1.2, from which we highlight the box-constraint that we discuss next.

### Remark 1.3

(box constraint as a stopping criterion) To prove the existence of a solution to the learning scheme $({\mathscr {L}}\!{\mathscr {S}})_{{TV\!}_\omega }$ in (1.4), we adopt the usual box-constraint approach in which we replace $\alpha \in \mathbb {R}^+$ by

1.10 $$\begin{aligned} \begin{aligned} \alpha \in \bigg [c_0,\frac{1}{c_0}\bigg ]\quad \text { for some } c_0\in (0,1). \end{aligned} \end{aligned}$$

In this case, the analog of (1.5) becomes

1.11 $$\begin{aligned} \bar{\alpha }_L=\inf \left\{ {{\,\textrm{arginf}\,}}\left\{ \int _L |u_c- u_{\alpha ,L}|^2\;\text {d}x:\,\alpha \in \big [c_0,\tfrac{1}{c_0}\big ]\right\} \right\} .\nonumber \\ \end{aligned}$$

Under some assumptions on the training data, we prove in Subsect. 3.4 (see Theorem 1.4) that this box constraint is equivalent to the existence of a stopping criterion for the refinement of the admissible partitions as in Definition 1.2.

### Theorem 1.4

(Equivalence between box constraint and stopping criterion) Consider the learning scheme $({\mathscr {L}}\!{\mathscr {S}})_{{TV\!}_\omega }$ in (1.4). The two following statements hold:

- If we replace (1.5) by (1.11), then there exists a stopping criterion $(\mathscr {S})$ for the refinement of the admissible partitions as in Definition 1.2.
- Assume that there exists a stopping criterion $(\mathscr {S})$ for the refinement of the admissible partitions as in Definition 1.2 such that the training data satisfy for all , with $\bar{\mathscr {P}}$ as in Definition 1.2, the conditions
  (i) $$TV(u_c,L) < TV(u_\eta ,L)$$;
  (ii) $$ \Vert u_\eta - u_c\Vert ^2_{L^2(L)} <\Vert [u_\eta ]_L - u_c\Vert ^2_{L^2(L)} $$, where $$[ u_\eta ]_L=\frac{1}{|L|}\int _L u_\eta \;\text {d}x. $$
  Then, there exists $c_0\in \mathbb {R}^+$ such that the optimal solution $u^*$ provided by $({\mathscr {L}}\!{\mathscr {S}})_{{TV\!}_\omega }$ with $\mathscr {P}$ replaced by $\bar{\mathscr {P}}$ coincides with the optimal solution $u^*$ provided by $({\mathscr {L}}\!{\mathscr {S}})_{{TV\!}_\omega }$ with (1.5) replaced by (1.11).

Next, we state our main theorem regarding existence of solutions for the learning scheme $({\mathscr {L}}\!{\mathscr {S}})_{{TV\!}_\omega }$ in (1.4). We state this result under the box-constraint condition. However, in view of Theorem 1.4, this result holds true under any stopping criterion for the refinement of the admissible partitions, and in particular if the training data satisfies the conditions (i) and (ii) above.

### Theorem 1.5

(Existence of solutions to $({\mathscr {L}}\!{\mathscr {S}})_{{TV\!}_\omega }$) There exists an optimal solution $u^*$ to the learning scheme $({\mathscr {L}}\!{\mathscr {S}})_{{TV\!}_\omega }$ in (1.4), whenever (1.5) is replaced by (1.11) for some fixed $c_0 \in (0,1).$

The proofs of Theorems 1.4 and 1.5 are presented in Sect. 3, where we also explore alternative stopping criteria.

As shown in [47, Theorem 2.4.17], given a positive, bounded, and Lipschitz continuous function $\omega :Q\rightarrow (0,\infty )$ with $\nabla \omega \in BV(Q;\mathbb {R}^2)$, the solution of (1.7) with $\omega _{\mathscr {L}}$ replaced by $\omega$ may exhibit jumps inherited from the weight $\omega$ that are not present in the data $u_\eta$, see Fig. 2 for a numerical example. Because $\omega _{\mathscr {L}}$ in Level 2 is constructed using the local optimal parameters given by Level 3, we heuristically expect that, in most applications, these extra jumps do not induce clearly visible artifacts. However, this possible issue has led us to consider two alternative adaptive space-dependent learning schemes.

First, we consider a learning scheme based on $({\mathscr {L}}\!{\mathscr {S}})_{{TV\!}_\omega }$ in (1.4) with $\omega _{\mathscr {L}}$ replaced by a smooth regularization $(\omega _\epsilon )_{\mathscr {L}}$ (see the regularized weighted TV learning scheme $({\mathscr {L}}\!{\mathscr {S}})_{{TV\!}_{\omega _\epsilon }}$ in (1.12)). Second, using the fact that the minimizer in (1.6) coincides with

$$\begin{aligned} \begin{aligned} {\text {argmin}}\left\{ \frac{1}{\alpha }\int _{L}|u_\eta -u|^2\;\text {d}x+ TV(u,L)\!:\,u\in BV(L)\right\} , \end{aligned} \end{aligned}$$

we consider the weighted-fidelity learning scheme $({\mathscr {L}}\!{\mathscr {S}})_{{TV- Fid}_\omega }$ in (1.16) below, where the weight appears in the fidelity term. Let us point out that a detailed analysis of the differences arising between weighted-fidelity and weighted-regularization parameter for TV has been carried out in the one-dimensional case in [44].

We begin by describing the regularized scenario.

1.12 $$\begin{aligned}&({\mathscr {L}}\!{\mathscr {S}})_{{TV\!}_{\omega _\epsilon }} { \textbf{Regularized}~ weighted-TV~ learning~ scheme}&\end{aligned}$$

**Level 3.**: (optimal local training parameter) Fix $\mathscr {L}\in \mathscr {P}$; for each $L\in \mathscr {L}$, find
  1.13 $$\begin{aligned} \alpha _L=\inf \left\{ {{\,\textrm{arginf}\,}}\left\{ \int _L |u_c- u_{\alpha ,L}|^2\,\;\text {d}x\!:\,\alpha \in \mathbb {R}^+\right\} \right\} ,\nonumber \\ \end{aligned}$$
  where, for $$\alpha \in \mathbb {R}^+$$,
  $$\begin{aligned} \begin{aligned} u_{\alpha ,L}:= {\text {argmin}}\Bigg \{\int _{L}|u_\eta&-u|^2\;\text {d}x+\alpha TV(u,L)\\  &:\,u\in BV(L)\Bigg \}. \end{aligned} \end{aligned}$$
**Level 2.**: (space-dependent image denoising) For each $\mathscr {L}\in$ $\mathscr {P}$ and for $\epsilon >0$ fixed, find
  $$\begin{aligned} \begin{aligned}&u^\epsilon _{\mathscr {L}}:={\text {argmin}}\bigg \{\int _Q|u_\eta -u|^2\;\text {d}x+TV_{\omega ^\epsilon _{\mathscr {L}}}(u,Q)\\&\quad \quad :\,u\in BV_{\omega ^\epsilon _{\mathscr {L}}}(Q)\bigg \}, \end{aligned} \end{aligned}$$
  where we consider a regularized weight $\omega ^\epsilon _{\mathscr {L}}:Q\rightarrow [0,\infty )$ of $\omega _{\mathscr {L}}$ in (1.8) such that
  1.14 $$\begin{aligned} \begin{aligned}&\omega ^\epsilon _{\mathscr {L}} \in C^1(Q) \hspace{5.0pt}\text { and } \hspace{5.0pt}\omega ^\epsilon _{\mathscr {L}} \nearrow \omega _{\mathscr {L}} \text { as}\, \epsilon \rightarrow 0^+\\  &\text { and a.e. in }Q. \end{aligned} \end{aligned}$$
**Level 1.**: (optimal partition and image restoration) Find
  $$\begin{aligned} \begin{aligned}&u^*_\epsilon \in {\text {argmin}}\left\{ \int _Q|u_c-u^\epsilon _{\mathscr {L}}|^2\;\text {d}x:\,\mathscr {L}\in \mathscr {P}\right\} \quad \\  &\text {with }u^\epsilon _{\mathscr {L}}\text { given by Level 2}. \end{aligned} \end{aligned}$$

For each $\epsilon >0$ fixed, similar results to those regarding the learning scheme $({\mathscr {L}}\!{\mathscr {S}})_{{TV\!}_\omega }$ in (1.4) hold for the learning scheme $({\mathscr {L}}\!{\mathscr {S}})_{{TV\!}_{\omega _\epsilon }}$ in (1.12). A natural question is whether a sequence of optimal solutions of the latter, $\{u^*_\epsilon \}_\epsilon$, converge in some sense to an optimal solution of the former, $u^*$, as $\epsilon \rightarrow 0^+$. This turns out to be an interesting mathematical question (see Remark 4.3), which we partially address in the following proposition.

### Proposition 1.6

(On the energies in $({\mathscr {L}}\!{\mathscr {S}})_{{TV\!}_{\omega _\epsilon }}$ as $\epsilon \rightarrow 0^+$) Under the setup of the learning schemes $({\mathscr {L}}\!{\mathscr {S}})_{{TV\!}_\omega }$ and $({\mathscr {L}}\!{\mathscr {S}})_{{TV\!}_{\omega _\epsilon }}$ above, fix $\mathscr {L}\in \mathscr {P}$ and let $E_\mathscr {L}:L^1(Q)\rightarrow [0,\infty ]$ and $E_\mathscr {L}^\epsilon :L^1(Q)\rightarrow [0,\infty ]$ be the functionals defined for $u\in L^1(Q)$ by

$$\begin{aligned} \begin{aligned}&E_\mathscr {L}[u]\!:=\!{\left\{ \begin{array}{ll} \int _Q|u_\eta \!-\!u|^2\;\text {d}x \!+\!TV_{\omega _\mathscr {L}}(u,Q) & \text {if } u\in BV_{\omega _{\mathscr {L}}}(Q),\\ \!+\!\infty & \text {otherwise}, \end{array}\right. }\\&E_\mathscr {L}^\epsilon [ u]\!:=\!{\left\{ \begin{array}{ll} \int _Q|u_\eta \!-\!u|^2\;\text {d}x \!+\!TV_{\omega ^\epsilon _{\mathscr {L}}}(u,Q) & \text {if } u\in BV_{\omega ^\epsilon _{\mathscr {L}}}(Q),\\ \!+\!\infty & \text {otherwise}. \end{array}\right. } \end{aligned} \end{aligned}$$

If (1.14) holds, then

1.15 $$\begin{aligned} \begin{aligned}&\Gamma (L^1(Q))-\limsup _{\epsilon \rightarrow 0^+} E_\mathscr {L}^\epsilon \leqslant E_\mathscr {L}. \end{aligned} \end{aligned}$$

Inequality (1.15) states, roughly speaking, that the asymptotic behavior of the functionals $E_\mathscr {L}^\epsilon$ is bounded from above by $E_\mathscr {L}$, for it can be equivalently expressed as

$$\begin{aligned} \inf \left\{ \limsup _{\epsilon \rightarrow 0^+} E_\mathscr {L}^\epsilon [u_{\epsilon }]:\, u_{\epsilon }\rightarrow u\quad \text {strongly in }L^1(Q)\right\} \leqslant E_\mathscr {L}[u] \end{aligned}$$

for every $u\in L^1(Q)$. The proof of this proposition and an analytical discussion of the learning scheme $({\mathscr {L}}\!{\mathscr {S}})_{{TV\!}_{\omega _\epsilon }}$ in (1.12) can be found in Sect. 4, while the corresponding numerical scheme is detailed in Sect. 6.

Next, we study the weighted-fidelity learning scheme $({\mathscr {L}}\!{\mathscr {S}})_{{TV-Fid}_\omega }$ motivated above.

1.16 $$\begin{aligned}&({\mathscr {L}}\!{\mathscr {S}})_{{TV-Fid}_\omega } { \textbf{Weighted}-fidelity~ learning~ scheme}&\end{aligned}$$

**Level 3.** (optimal local training parameter) Fix $\mathscr {L}\in \mathscr {P}$; for each $L\in \mathscr {L}$, find

1.17 $$\begin{aligned} \alpha _L=\inf \left\{ {{\,\textrm{arginf}\,}}\left\{ \int _L |u_c- u_{\alpha ,L}|^2\,\;\text {d}x\!:\,\alpha \in \mathbb {R}^+\right\} \right\} ,\nonumber \\ \end{aligned}$$

where, for $$\alpha \in \mathbb {R}^+$$,

1.18 $$\begin{aligned} \begin{aligned} u_{\alpha ,L}&:= {\text {argmin}}\left\{ \int _{L}\frac{1}{\alpha }|u_\eta -u|^2\;\text {d}x+ TV(u,L)\right. \\  &\left. :\,u\in BV(L)\right\} . \end{aligned} \end{aligned}$$

**Level 2.** (space-dependent image denoising) For each $\mathscr {L}\in \mathscr {P}$, find

$$\begin{aligned} \begin{aligned} u_{\mathscr {L}}:={\text {argmin}}\bigg \{\int _Q\frac{1}{\omega _\mathscr {L}}|u_\eta&-u|^2\;\text {d}x +TV(u,Q)\\  &:\,u\in BV_{\omega _{\mathscr {L}}}(Q)\bigg \}, \end{aligned} \end{aligned}$$

where, similarly to (1.8), $\omega _{\mathscr {L}}$ is defined by

$$\begin{aligned} \begin{aligned} \omega _{\mathscr {L}}(x):=\sum _{L\in \mathscr {L} }\alpha _L \chi _L(x) \quad \text {with }\alpha _L\text { given by Level 3}. \end{aligned} \end{aligned}$$

**Level 1.** (optimal partition and image restoration) Find

$$\begin{aligned} \begin{aligned}&u^*\in {\text {argmin}}\left\{ \int _Q|u_c-u_{\mathscr {L}}|^2\;\text {d}x:\,\mathscr {L}\in \mathscr {P}\right\} \\&\text {with }u_{\mathscr {L}}\text { given by Level 2}. \end{aligned} \end{aligned}$$

Once more, similar results to those regarding the learning scheme $({\mathscr {L}}\!{\mathscr {S}})_{{TV\!}_\omega }$ in (1.4) hold for the learning scheme $({\mathscr {L}}\!{\mathscr {S}})_{{TV-Fid}_\omega }$ in (1.16). In particular, the box constraint here is essential to guarantee that **Level 2** of the scheme is well posed. This analysis is undertaken in Sect. 4, while the corresponding numerical study is addressed in Sect. 6.

The last theoretical result of this paper concerns replacing the *TV* term in our space-dependent bilevel learning schemes with a higher-order regularizer. A well-known drawback of the ROF model is the possible occurrence of staircasing effects whenever two neighboring areas of an image are both smoothed out and an abrupt spurious discontinuity is produced in the denoising process. To counteract this effect a canonical solution (among others like the use of Huber-type smoother approximations of the total variation as in [13]) consists in resorting to higher-order derivatives in the regularizer (see, e.g., [10, 21, 28, 56]). We consider here the total generalized variation (*TGV*) model introduced in [11], which is considered to be one of the most effective image-reconstruction models among those involving mixed first- and higher-order terms, cf. [12, 46, 56, 59] for some theoretical results about its solutions.

For a function $u\in BV(Q)$ and $\alpha =(\alpha _0,\alpha _1)\in \mathbb {R}^+\times \mathbb {R}^+$, the second-order *TGV* functional is given by

1.19 $$\begin{aligned} TGV_{\alpha _0,\alpha _1}(u):={{\min }}\big \{\alpha _0|D u  &   -v|(Q)+\alpha _1|\mathcal {E}v|(Q)\nonumber \\    &   \quad : v\in BD(Q)\big \},\nonumber \\ \end{aligned}$$

where, as before, *Du* denotes the distributional gradient of *u*, $|\mu |(Q)$ is the total variation on $Q$ of a Radon measure $\mu$, $\mathcal {E}$ is the symmetric part of the distributional gradient, and *BD* indicates the space of vector-valued functions with bounded deformation, cf. [62]. In this setting, our learning scheme reads as follows.

1.20 $$\begin{aligned}&({\mathscr {L}}\!{\mathscr {S}})_{{TGV\!}_\omega } { \textbf{Weighted}-TGV~ learning~ scheme}&\end{aligned}$$

**Level 3.** (optimal local regularization parameter) Fix $\mathscr {L}\in \mathscr {P}$; for each $L\in \mathscr {L}$, find

1.21 $$\begin{aligned} \begin{aligned} \alpha _L\!=\!\big ((\alpha _L)_0,(\alpha _L)_1\big )\!:=\! \inf \Bigg \{{{\,\textrm{arginf}\,}}\Bigg \{\!\int _L&|u_c\!-\! u_{\alpha ,L}|^2\,\;\text {d}x\\  &:\,\alpha \!=\!(\alpha _0,\alpha _1)\in \mathbb {R}^+\!\times \! \mathbb {R}^+\Bigg \}\Bigg \}, \end{aligned}\nonumber \\ \end{aligned}$$

where, for $$\alpha =(\alpha _0,\alpha _1)\in \mathbb {R}^+\times \mathbb {R}^+$$,

1.22 $$\begin{aligned} \begin{aligned} u_{\alpha ,L}:= {\text {argmin}}\Bigg \{ \int _{L}|u_\eta -u|^2&\;\text {d}x+ TGV_{\alpha _0,\alpha _1}(u,L)\\  &\quad :\,u\in BV(L)\Bigg \}, \end{aligned}\nonumber \\ \end{aligned}$$

and where the infimum in (1.21) is meant with respect to the lexicographic order in $\mathbb {R}^2$.

**Level 2.** (space-dependent TGV image denoising) For each $\mathscr {L}\in \mathscr {P}$, find

1.23 $$\begin{aligned} \begin{aligned} u_{\mathscr {L}}:={\text {argmin}}\bigg \{\int _Q |u_\eta -u|^2&\;\text {d}x +TGV_{\omega _{\mathscr {L}}^0,\omega _{\mathscr {L}}^1}(u,Q)\\  &\quad :\,u\in BV_{\omega _{\mathscr {L}}^0}(Q)\bigg \}, \end{aligned}\nonumber \\ \end{aligned}$$

where, for $i\in \{0,1\}$, the weight $\omega _{\mathscr {L}}^i$ is defined by

$$\begin{aligned} \begin{aligned} \omega _{\mathscr {L}}^i(x):=\sum _{L\in \mathscr {L} }(\alpha _L)_i\, \chi _L(x) \quad \text {with }\alpha _L\text { given by Level 3}. \end{aligned} \end{aligned}$$

In the expression above,

1.24 $$\begin{aligned}&TGV_{\omega _{\mathscr {L}}^0,\omega _{\mathscr {L}}^1}(u,Q):=\nonumber \\&\quad \inf _{v\in BD_{\omega _{\mathscr {L}}^1}(Q)}\left\{ \mathscr {V}_{\omega _{\mathscr {L}}^0}(Du-v,Q)+{\mathscr {V}_{\omega _{\mathscr {L}}^1}}(\mathcal {E}v,Q)\right\} , \end{aligned}$$

where the quantities $\mathscr {V}_{\omega _{\mathscr {L}}^0}$ and $\mathscr {V}_{\omega _{\mathscr {L}}^1}$ are weighted counterparts to the classical total variation of Radon measures. We refer to Sects. 2 and 5 for the precise definition and properties of these quantities. In particular, we will prove that

1.25 $$\begin{aligned} \mathscr {V}_{\omega _{\mathscr {L}}^0}(Du-v,Q)=\int _Q (\omega _{\mathscr {L}}^0)^{\text {sc}^-}\text {d}|Du-v|, \end{aligned}$$

and

1.26 $$\begin{aligned} \mathscr {V}_{\omega _{\mathscr {L}}^1}(\mathcal {E}v,Q)=\int _Q (\omega _{\mathscr {L}}^1)^{\text {sc}^-}\text {d}|\mathcal {E}v|, \end{aligned}$$

where $BV_{\omega _{\mathscr {L}}^0}$ is the space of $\omega _{\mathscr {L}}^0$-weighted *BV*-functions (see Subsect. 3.2) and $BD_{\omega _{\mathscr {L}}^1}$ is the space of $\omega _{\mathscr {L}}^1$-weighted *BD*-functions (see Sect. 5).

**Level 1.** (optimal partition and image restoration) Find

$$\begin{aligned} \begin{aligned}&u^*\in {\text {argmin}}\left\{ \int _Q|u_c-u_{\mathscr {L}}|^2\;\text {d}x:\,\mathscr {L}\in \mathscr {P}\right\} \\&\text {with }u_{\mathscr {L}}\text { given by Level 2}. \end{aligned} \end{aligned}$$

Analogously to $({\mathscr {L}}\!{\mathscr {S}})_{{TV-Fid}_\omega }$, we can also consider a weighted-fidelity TGV scheme, which we use in our numerical results and describe next.

1.27 $$\begin{aligned}&({\mathscr {L}}\!{\mathscr {S}})_{{TGV-Fid}_\omega } { \textbf{TGV}~ weighted-fidelity~ learning~ scheme}&\end{aligned}$$

With $\alpha _0,\alpha _1 \in \mathbb {R}^+$ fixed throughout:

**Level 3.** (optimal local training parameter) Fix $\mathscr {L}\in \mathscr {P}$; for each $L\in \mathscr {L}$, find

1.28 $$\begin{aligned} \lambda _L=\inf \left\{ {{\,\textrm{arginf}\,}}\left\{ \int _L |u_c- u_{\lambda ,L}|^2\,\;\text {d}x\!:\,\lambda \in \mathbb {R}^+\right\} \right\} ,\nonumber \\ \end{aligned}$$

where, for $$\lambda \in \mathbb {R}^+$$,

$$\begin{aligned} \begin{aligned} u_{\lambda ,L}:= {\text {argmin}}\Bigg \{\lambda \int _{L}|u_\eta -u|^2&\;\text {d}x+ TGV_{\alpha _0, \alpha _1}(u,L)\\  &\quad :\,u\in BV(L)\Bigg \}. \end{aligned} \end{aligned}$$

**Level 2.** (space-dependent image denoising) For each $\mathscr {L}\in \mathscr {P}$, find

$$\begin{aligned} \begin{aligned} u_{\mathscr {L}}:={\text {argmin}}\bigg \{\int _Q \omega _\mathscr {L} |u_\eta -u|^2&\;\text {d}x +TGV_{\alpha _0, \alpha _1}(u,Q)\\  &:\,u\in BV_{\omega _{\mathscr {L}}}(Q)\bigg \}, \end{aligned} \end{aligned}$$

where $\omega _{\mathscr {L}}$ is defined by

$$\begin{aligned} \begin{aligned} \omega _{\mathscr {L}}(x):= \sum _{L\in \mathscr {L} }\lambda _L \chi _L(x) \quad \text {with }\lambda _L\text { given by Level 3}. \end{aligned} \end{aligned}$$

**Level 1.** (optimal partition and image restoration) Find

$$\begin{aligned} \begin{aligned}&u^*\in {\text {argmin}}\left\{ \int _Q|u_c-u_{\mathscr {L}}|^2\;\text {d}x:\,\mathscr {L}\in \mathscr {P}\right\} \\  &\text {with }u_{\mathscr {L}}\text { given by Level 2}. \end{aligned} \end{aligned}$$

As in the case of our learning schemes for the weighted total variation, the analysis of $({\mathscr {L}}{\mathscr {S}})_{TGV_\omega }$ and $({\mathscr {L}}{\mathscr {S}})_{TGV } { - \textrm{Fid}_\omega }$ is performed under a box-constraint assumption, which for the first case reads as

1.29 $$\begin{aligned} \alpha = (\alpha _0, \alpha _1) \in \left[ c_0,\frac{1}{c_0}\right] \times \left[ c_1,\frac{1}{c_1}\right] . \end{aligned}$$

Our main result for the weighted-*TGV* scheme is the following.

### Theorem 1.7

(Existence of solutions to $({\mathscr {L}}\!{\mathscr {S}})_{{TGV\!}_\omega }$) There exists an optimal solution $u^*$ to the learning scheme $({\mathscr {L}}\!{\mathscr {S}})_{{TGV\!}_\omega }$ in (1.20) with the minimization in (1.21) restricted by (1.29).

Analogously, we infer the ensuing theorem for the *TGV* with weighted fidelity.

### Theorem 1.8

(Existence of solutions to $({\mathscr {L}}\!{\mathscr {S}})_{{TGV-Fid}_\omega }$) For every $c\in (0,1)$, there exists an optimal solution $u^*$ to the learning scheme $({\mathscr {L}}\!{\mathscr {S}})_{{TGV-Fid}_\omega }$ in (1.27) with the minimization in (1.28) restricted by the box constraint $\lambda \in \left[ c,\frac{1}{c}\right]$.

Also in the case of weighted-*TGV* learning schemes, we provide a connection between stopping criteria and existence of a box constraint. To be precise, we show that if (1.29) is imposed, then a stopping criterion can be naturally imposed on the schemes. Concerning the converse implication, we show that if a suitable stopping criterion is enforced, then $(\alpha _L)_0$ and $(\alpha _L)_1$ are both always bounded from below by a positive constant and that they cannot simultaneously blow up to infinity. The weaker nature of this latter implication is due to one main reason: the upper bound established on the optimal parameters for the weighted *TV* scheme is hinged upon a suitable Poincaré inequality for the total variation functional, cf. Proposition 3.5; in the *TGV* case, the analogous argument only provides a bound from above for the minimum between $(\alpha _L)_0$ and $(\alpha _L)_1$ and thus does not allow to conclude the existence of a uniform upper bound on either component, cf. Proposition 5.11. We refer to Subsect. 5.3 for a discussion of this issue and for the details of this argument. For completeness, we mention that a result related to Proposition 5.11 has been proven in [57, Proposition 6]. In Proposition 5.11, we make this study quantitative and keep track of the dependence on the cell size through the Poincaré constant.

The results we present suggest a number of possible directions and questions for future research. One possible avenue is the formulation of similar schemes with piecewise constant weights in the case of Mumford–Shah regularizations, relating to the Ambrosio–Tortorelli scheme of [40] which explicitly allows for discontinuous weights. Another is an investigation of the relation and apparent discrepancy between our results concluding stopping of the refinement of partitions, in which parameter variations at very fine scales are not advantageous, and numerical results in the literature where wildly varying parameter maps appear in the optimization, such as in [48].

The paper is organized as follows: in Sect. 2, we collect some notation which will be employed throughout the paper. The focus of Sects. 3 and 4 is on our weighted-*TV* scheme, as well as on the two variants thereof, including a regularization of the weight and a weighted fidelity, respectively. Section 5 is devoted to the study of our weighted-*TGV* learning scheme and of the corresponding *TGV* scheme with weighted fidelity. Section 6 contains some numerical results for the various learning schemes presented in the paper and a comparison of their performances.

## Glossary

Here we collect some notation that will be used throughout the paper, and introduce some energy functionals that will be studied.

We start by addressing our admissible partitions of the unit cube $Q=(0,1)^2$ into dyadic squares. For $\kappa \in \mathbb {N}_0$, let

$$\begin{aligned} \begin{aligned} Z_\kappa :=\left\{ 2^{-\kappa }z \in [0,1)^2:z\in \mathbb {Z}^2 \right\} . \end{aligned} \end{aligned}$$

For instance, $Z_0=\{(0,0)\}$ and $Z_1=\{(0,0),(0,\frac{1}{2}), (\frac{1}{2},0), (\frac{1}{2},\frac{1}{2})\}.$ Note that $Z_k$ has cardinality $2^k\times 2^k$, which allow us to write $Z_\kappa = \cup _{\iota =1}^{4^\kappa }z_\iota ^{(\kappa )}$, where $z_\iota ^{(\kappa )}=2^{-\kappa }z_\iota$ for a convenient $z_\iota \in \mathbb {Z}^2$. Then, for each $\kappa \in \mathbb {N}_0$ and $\iota \in \{1,...,4^\kappa \}$, we consider the dyadic square

$$\begin{aligned} \begin{aligned} Q_\iota ^\kappa :=\left( z_\iota ^{(\kappa )}+\left( 0,\frac{1}{2^\kappa }\right] ^2 \right) \cap Q. \end{aligned} \end{aligned}$$

For each $\kappa \in \mathbb {N}_0$ fixed, we have that $Q_{\iota _1}^\kappa \cap Q_{\iota _2}^\kappa =\emptyset$ for every $\iota _1, \iota _2 \in \{1,...,4^\kappa \}$ with $\iota _1\not =\iota _2$; moreover, $Q=\cup _{\iota =1}^{4^\kappa } Q_\iota ^\kappa$. In particular, $\mathscr {L}:=\{Q_\iota ^\kappa :\iota \in \{1,...,4^\kappa \}\}$ provides an example of an admissible partition of $Q$. More generally, recalling that we denote by $\mathscr {P}$ the class of all admissible partitions $\mathscr {L}$ of $Q$ consisting of dyadic squares as above, then if $\mathscr {L}\in \mathscr {P}$ and $L\in \mathscr {L}$ are arbitrary, there exist $\kappa \in \mathbb {N}_0$ and $\iota \in \{1,...,4^\kappa \}$ such that $L=Q_\iota ^\kappa$.

The setting of our work is a two-dimensional one, mainly due to the scale invariance of the constant in the two-dimensional Poincaré–Wirtinger inequality in $BV$, as discussed in the proof of Proposition 3.1. This invariance is crucial to prove existence of solutions for our schemes (see, for instance, Theorem 3.6). However, there are some theoretical results concerning the weighted-$BV$ and weighted-$TGV$ spaces that hold in any dimension $n\in \mathbb {N}$, for which reason we state such results in $\mathbb {R}^n$.

In what follows, $\Omega \subset \mathbb {R}^n$ is an open and bounded set and $\mathbb {X}$ stands for either $\mathbb {R}$, $\mathbb {R}^{n},$ or $\mathbb {R}^{n\times n}_{sym}$, where the latter is the space of all $n\times n$ symmetric matrices and $n\in \mathbb {N}$. We denote by $\mathcal {M}(\Omega ;\mathbb {X})$ the space of all finite Radon measures in $\Omega$ with values on $\mathbb {X}$, and by $|\mu |\in \mathcal {M}(\Omega ;\mathbb {R}^+_0)$ the total variation of $\mu \in \mathcal {M}(\Omega ;\mathbb {X})$, which is defined for each measurable set $B\subset \Omega$ by

$$\begin{aligned} \begin{aligned} |\mu |(B):= \sup \bigg \{\sum _{i=1}^\infty |\mu (B_i)|: \,\, \{B_i\}_{i\in \mathbb {N}} \text { is a partition of } B\bigg \}. \end{aligned} \end{aligned}$$

Using the Riesz representation theorem, $\mathcal {M}(\Omega ;\mathbb {X})$ can be identified with the dual of $C_0(\Omega ;\mathbb {X}')$, the closure with respect to the supremum norm of the set of all continuous functions on $\Omega$ with compact support. In particular, the total variation of a Radon measure $\mu \in \mathcal {M}(\Omega ;\mathbb {X})$ is alternatively given by

2.1 $$\begin{aligned} |\mu |(B) = \sup \bigg \{  &   \int _B \varphi (x)\cdot \! \;\text {d}\mu (x):\ \varphi \in C_0(B;\mathbb {X}'), \,\,\nonumber \\    &   \Vert \varphi \Vert _{L^\infty (B;\mathbb {X}')} \leqslant 1 \bigg \}, \hspace{5.0pt}B\subset \Omega \text { measurable,}\nonumber \\ \end{aligned}$$

where $\cdot$ represents the duality product between an element of $\mathbb {X}'$ and an element of $\mathbb {X}$. With the trivial identification of column vectors with row vectors, we will often write $\mathbb {X}$ in place of $\mathbb {X}'$.

In the case in which $\mu = Du\in \mathcal {M}(\Omega ;\mathbb {R}^n)$ for some $u\in BV(\Omega )$, a density argument shows that (2.1) is equivalent to

2.2 $$\begin{aligned} \begin{aligned} |Du|(B) =\sup \bigg \{ \int _B u(x)\,&{{\,\textrm{div}\,}}\varphi (x) \;\text {d}x:\ \varphi \in {{\,\textrm{Lip}\,}}_c(B;\mathbb {R}^n), \\  &\quad \Vert \varphi \Vert _{L^\infty (B;\mathbb {R}^n)} \leqslant 1 \bigg \}, \end{aligned}\nonumber \\ \end{aligned}$$

and we often write $TV(u,B)$ in place of $|Du|(B)$. In the preceding expression, and throughout this manuscript, ${{\,\textrm{Lip}\,}}_c(B;\mathbb {X})$ represents the space of all $\mathbb {X}$-valued Lipschitz functions with compact support in $B$.

Similarly, in the case in which $\mu = \mathcal {E}v\in \mathcal {M}(\Omega ;\mathbb {R}^{n\times n}_{sym})$ for some $v\in BD(\Omega )$ and $\mathcal {E}$ the symmetrical part of the distributional derivative, then (2.1) is equivalent to

2.3 $$\begin{aligned} |\mathcal {E}v|(B) =\sup \bigg \{ \int _B v(x)  &   \cdot {{\,\textrm{div}\,}}\varphi (x) \;\text {d}x:\ \varphi \in {{\,\textrm{Lip}\,}}_c(B;\mathbb {R}^{n\times n}_{sym}), \,\,\nonumber \\    &   \qquad \Vert \varphi \Vert _{L^\infty (B;\mathbb {R}^{n\times n}_{sym})} \leqslant 1 \bigg \},\nonumber \\ \end{aligned}$$

where $({\text {div}}\, \varphi )_j = \sum _{k=1}^n \frac{\partial \varphi _{jk}}{\partial x_k}$ for each $j\in \{1,...,n\}$.

At the core of the present manuscript are weighted versions of the spaces of bounded variation and of bounded deformation. These weighted versions rely on a generalization of (2.2) and (2.3) that cannot be derived directly from the Riesz representation theorem, and thus need a careful analysis to prove the variational identities stated in (1.9) and (1.25)–(1.26), addressed in Sects. 3 and 5, respectively.

Given a Radon measure $\mu \in \mathcal {M}(\Omega ;\mathbb {X})$ and a locally integrable function $\omega :\Omega \rightarrow [0,\infty )$, we define the $\omega$-weighted variation of $\mu$ on $\Omega$, written $\mathscr {V}_\omega (\mu ,\Omega )$, by

2.4 $$\begin{aligned} \mathscr {V}_\omega (\mu ,\Omega ):=\sup \bigg \{  &   \int _{\Omega } \varphi (x)\cdot \! \;\text {d}\mu (x):\nonumber \\ \  &   \quad \varphi \in {{\,\textrm{Lip}\,}}_c(\Omega ;\mathbb {X}'), \,\, | \varphi | \leqslant \omega \bigg \}.\nonumber \\ \end{aligned}$$

As before, if $\mu = Du\in \mathcal {M}(\Omega ;\mathbb {R}^n)$ for some $u\in BV(\Omega )$, then (2.4) is equivalent to

$$\begin{aligned} \mathscr {V}_\omega (Du,\Omega ) =\sup \bigg \{  &   \int _{\Omega } u(x)\, {{\,\textrm{div}\,}}\varphi (x)\\  &   \;\text {d}x:\ \varphi \in {{\,\textrm{Lip}\,}}_c(\Omega ;\mathbb {R}^n), \,\, | \varphi | \leqslant \omega \bigg \}, \end{aligned}$$

which we often represent by $TV_\omega (u,\Omega )$, and we define

$$\begin{aligned} \begin{aligned} BV_{\omega }(\Omega )&:=\Bigg \{ u:\Omega {\rightarrow } \mathbb {R}\text { measurable:} \int _\Omega |u(x)|\, \omega (x)\;\text {d}x{<}\infty \\  &\qquad \qquad \qquad \text { and } TV_{\omega }(u,\Omega )<\infty \Bigg \}. \end{aligned} \end{aligned}$$

Also, if $\mu = Du-v:= Du-v\mathcal {L}^n\lfloor \Omega \in \mathcal {M}(\Omega ;\mathbb {R}^n)$ for some $u\in BV(\Omega )$ and $v\in L^1(\Omega ;\mathbb {R}^n)$, then (2.4) is equivalent to

$$\begin{aligned} \begin{aligned} \mathscr {V}_\omega (Du\!-\!v,\Omega ) \!{=}\!\sup \bigg \{ \int _{\Omega } \big (u(x)\,&{{\,\textrm{div}\,}}\varphi (x) \!{+}\!v(x) \cdot \varphi (x)\big ) \;\text {d}x\\  &:\ \varphi \!\in \! {{\,\textrm{Lip}\,}}_c(\Omega ;\mathbb {R}^n), \,\, | \varphi | \!\leqslant \! \omega \bigg \}. \end{aligned} \end{aligned}$$

Moreover, if $\mu = \mathcal {E}v\in \mathcal {M}(\Omega ;\mathbb {R}^{n\times n}_{sym})$ for some $v\in BD(\Omega )$, then (2.4) is equivalent to

$$\begin{aligned} \mathscr {V}_\omega (\mathcal {E}v,\Omega ) =\sup \quad \bigg \{  &   \int _{\Omega } v(x) \cdot {{\,\textrm{div}\,}}\varphi (x) \;\text {d}x:\ \\  &   \quad \varphi \in {{\,\textrm{Lip}\,}}_c(\Omega ;\mathbb {R}^{n\times n}_{sym}), \,\, | \varphi | \leqslant \omega \bigg \}, \end{aligned}$$

and we define

$$\begin{aligned} \begin{aligned} BD_{\omega }(\Omega ):=\Bigg \{ v:\Omega {\rightarrow }&\mathbb {R}\text { measurable:} \int _\Omega |v(x)|\, \omega (x)\;\text {d}x{<}\infty \, \\  &\text { and } \mathscr {V}_\omega (\mathcal {E}v,\Omega )<\infty \Bigg \}. \end{aligned} \end{aligned}$$

The energy functional associated with the analogue to the ROF’s model, where we use a weighted-TV regularizer on $\Omega \subset \mathbb {R}^2$ instead of the total variation (TV), is denoted by (see Theorem 3.2)

$$\begin{aligned} \begin{aligned} E[u]:= \int _\Omega |u_\eta -u|^2\;\text {d}x +TV_{\omega }(u,\Omega ). \end{aligned} \end{aligned}$$

To highlight the dependence on a partition $\mathscr {L}$ of $Q$ made of dyadic cubes, the extension of the preceding functional (for a weight $\omega _\mathscr {L}$ and $\Omega =Q$) to $L^1(Q)$ is represented by

$$\begin{aligned} \begin{aligned}&E_\mathscr {L}[u]:={\left\{ \begin{array}{ll} \int _Q|u_\eta -u|^2\;\text {d}x +TV_{\omega _\mathscr {L}}(u,Q)&  \text {if } u\in BV_{\omega _{\mathscr {L}}}(Q),\\ +\infty & \text {otherwise}. \end{array}\right. } \end{aligned} \end{aligned}$$

Moreover, for the $\epsilon$-dependent regularized weight $\omega ^\epsilon _\mathscr {L}$, introduced in (1.14), the energy above is written as

$$\begin{aligned} \begin{aligned}&E_\mathscr {L}^\epsilon [ u]:={\left\{ \begin{array}{ll} \int _Q|u_\eta -u|^2\;\text {d}x +TV_{\omega ^\epsilon _{\mathscr {L}}}(u,Q)&  \text {if } u\in BV_{\omega ^\epsilon _{\mathscr {L}}}(Q),\\ +\infty &  \text {otherwise}. \end{array}\right. } \end{aligned} \end{aligned}$$

The two preceding functionals are introduced in Proposition 1.6, where we address the relationship between the weighted-TV and the regularized weighted-TV learning schemes in (1.4) and (1.12), respectively.

For a fixed image domain $\Omega \subset \mathbb {R}^2$, the optimal tuning parameter $\alpha$ in Level 3 of any of the *TV* learning schemes addressed here is found by minimizing the cost function $I:(0,\infty )\rightarrow \mathbb {R}$ defined by

2.5 $$\begin{aligned} \begin{aligned}&I(\alpha ):=\int _\Omega |u_c-u_\alpha |^2\;\text {d}x \text { for } \alpha \in (0,+\infty ), \end{aligned} \end{aligned}$$

where $u_c$ is the clean image and $u_\alpha$ is the reconstructed image obtained as the minimizer of the denoising model in aforementioned Level 3. In our analysis, we make use of the extension $\widehat{I}:[0,+\infty ]\rightarrow [0,+\infty ]$ of $I$ to the closed interval $[0,+\infty ]$ defined for $\bar{\alpha }\in [0,+\infty ]$ by

2.6 $$\begin{aligned} \begin{aligned} \widehat{I}(\bar{\alpha }):= \inf \Big \{\liminf _{j\rightarrow \infty } I(\alpha _j)&:\, (\alpha _j)_{j\in \mathbb {N}}\subset (0,+\infty ), \, \alpha _j \rightarrow \bar{\alpha }\\  &\text { in } [0,+\infty ]\Big \}, \end{aligned}\nonumber \\ \end{aligned}$$

which can be seen as the lower-semicontinuous envelope of $I$ on the closed interval $[0,+\infty ]$. As it turns out, $\widehat{I}$ is actually a continuous function on $[0,+\infty ]$ (cf. Corollary 3.11). The study of existence of minimizers for $I$ and the characterization of $\widehat{I}$ for the weighted-TV learning scheme in (1.4) is addressed in Theorem 3.8, Lemma 3.10, and Corollary 3.11. This study relies on the convergence of minimizers of the family, parametrized by $\alpha \in (0,\infty )$, of energy functionals associated with ROF’s model,

$$\begin{aligned} \begin{aligned}&F_\alpha [u]:={\left\{ \begin{array}{ll} \int _{\Omega }|u_\eta -u|^2\;\text {d}x+\alpha TV(u,\Omega ) & \text {if } u\in BV(\Omega ),\\ +\infty & \text {otherwise.} \end{array}\right. }\quad \end{aligned} \end{aligned}$$

In turn, this convergence analysis naturally involves the extreme points $\bar{\alpha } = 0$ and $\bar{\alpha }=+\infty$, which are associated with the energies

$$\begin{aligned} \begin{aligned}&F_0 [u]:={\left\{ \begin{array}{ll} \int _{\Omega }|u_\eta -u|^2\;\text {d}x & \text {if } u\in L^2(\Omega ),\\ +\infty & \text {otherwise,} \end{array}\right. }\\&\text {and}\quad F_{\infty } [u]:={\left\{ \begin{array}{ll} \int _{\Omega }|u_\eta -c|^2\;\text {d}x & \text {if } u\equiv c\in \mathbb {R}, \\ +\infty & \text {otherwise,} \end{array}\right. } \end{aligned} \end{aligned}$$

respectively (we remark that, since the local parameters in each dyadic square are constant, this analysis also applies for the weighted-TV learning scheme in (1.4)).

Regarding the $TGV$ case, to obtain the existence of optimal parameters for Level 3 of the schemes (1.20) and (1.27), stated in Theorem 5.13, we are led to study $\Gamma$-convergence of the family of functionals, parametrized by $\alpha =(\alpha _0,\alpha _1)\in (0,+\infty )^2$, defined as

$$\begin{aligned} \begin{aligned}&G_\alpha [u]:={\left\{ \begin{array}{ll} \int _{\Omega }|u_\eta -u|^2\;\text {d}x + TGV_{\alpha _0,\alpha _1}(u,\Omega ) & \text {if } u\in BV(\Omega ),\\ +\infty & \text {otherwise.} \end{array}\right. }\quad \end{aligned} \end{aligned}$$

In this case, the $\Gamma$-convergence result is more involved because it includes different combinations of $\bar{\alpha }_i = 0$, $\bar{\alpha }_i \in \mathbb {R}^+$, or $\bar{\alpha }_i = +\infty$ for $i=0$ and $i=1$. The expressions for the ensuing limits can be found in the statement of Lemma 5.15.

The characterization of the extension to the closed interval $[0,+\infty ]^2$ of the *TGV* analog of (2.5), denoted by $J(\alpha )$ for $\alpha =(\alpha _0, \alpha _1)$, is contained in Lemma 5.18.

In the sequel, we use both the average of a function $u:\Omega \rightarrow \mathbb {R}$ on a subdomain $L \subset \Omega$,

$$\begin{aligned} \begin{aligned} [u]_L:= \frac{1}{|L|}\int _L u(x)\;\text {d}x, \end{aligned} \end{aligned}$$

and its projection onto affine functions $\langle u\rangle _L$, which is the unique solution to the minimum problem

$$\begin{aligned} \min \left\{ \int _L |u-v|^2\;\text {d}x:\,v\text { is affine in }L\right\} , \end{aligned}$$

where in both cases the subscript may be omitted when $L = \Omega$.

## Analysis of the Weighted-TV Learning Scheme $({\mathscr {L}}\!{\mathscr {S}})_{{TV\!}_\omega }$

Here, we prove existence of solutions to the weighted-TV learning scheme, $({\mathscr {L}}\!{\mathscr {S}})_{{TV\!}_\omega }$, introduced in (1.4). We analyze each level in the three subsequent subsections. In particular, we prove Theorem 1.5 in Subsect. 3.3. Then, in Subsect. 3.4, we prove Theorem 1.4 and we provide different examples of stopping criteria for the refinement of the admissible partitions introduced in Definition 1.2.

### On Level 3

In this section, we discuss the main features of Level 3, and variants thereof, of the learning scheme $({\mathscr {L}}\!{\mathscr {S}})_{{TV\!}_\omega }$ in (1.4).

As we mentioned in Remark 1.1, the parameter $\alpha _L$ in (1.5) is uniquely determined by definition, with $\alpha _L\in [0,+\infty ]$. Then, in view of Theorem 3.8 (see Subsect. 3.4), if $L\in \mathscr {L}$ is such that

3.1 $$\begin{aligned} \begin{aligned}&TV(u_c,L)< TV(u_\eta ,L) \\&\text { and } \quad \Vert u_\eta - u_c\Vert ^2_{L^2(L)} <\Vert [ u_\eta ]_L - u_c\Vert ^2_{L^2(L)}, \end{aligned} \end{aligned}$$

then

$$\begin{aligned} \begin{aligned}&{{\,\textrm{arginf}\,}}\left\{ \int _L |u_c- u_{\alpha ,L}|^2\,\;\text {d}x\!:\,\alpha \in \mathbb {R}^+\right\} \\&= {\text {argmin}}\left\{ \int _L |u_c- u_{\alpha ,L}|^2\,\;\text {d}x\!:\,\alpha \in \big [c_L,C_Q\Vert u_\eta \Vert _{L^2(L)}\big ]\right\} , \end{aligned} \end{aligned}$$

where $c_L$ and $C_Q$ are positive constants, with $c_Q$ depending only on $Q$. In particular, we have that $\alpha _L{\in } \big [c_L,C_Q\Vert u_\eta \Vert _{L^2(L)}\big ].$ Furthermore, because each partition $\mathscr {L}\in {\mathscr {P}}$ is finite, it follows that if (3.1) holds for all $L\in \mathscr {L}$, then

$$\begin{aligned} \begin{aligned} \alpha _L\in K_{\mathscr {L}}:=\Big [\min _{L\in \mathscr {L}} c_L, C_Q\max _{L\in \mathscr {L}}\Vert u_\eta \Vert _{L^2(L)}\Big ] \subset (0,+\infty ) \end{aligned} \end{aligned}$$

for every $L\in \mathscr {L}$, which yields a natural box constraint for a fixed partition. Note, however, that the box constraint given by the compact set $K_{\mathscr {L}}$ may vary according to the choice of the partition $\mathscr {L}$.

Finally, if we consider Level 3 with (1.5) replaced by (1.11), then the minimum

$$\begin{aligned} \begin{aligned} \min _{\alpha \in [c_0,\frac{1}{c_0}]} \int _L |u_c- u_{\alpha ,L}|^2\,\;\text {d}x \end{aligned} \end{aligned}$$

exists as the minimum of a lower semicontinuous function (see Corollary 3.11 in Subsect. 3.4) on a compact set. In particular, $\bar{\alpha }_L$ is uniquely determined, with

$$\begin{aligned} \begin{aligned} \bar{\alpha }_L\in \Big [c_0,\frac{1}{c_0}\Big ] \text { for all }L\in \mathscr {L}\text { and }\mathscr {L}\in \mathscr {P}. \end{aligned} \end{aligned}$$

### On Level 2

Here, we discuss existence and uniqueness of solutions to the minimization problem in (1.7). A key step in this discussion is the study of the space $BV_{\omega }(\Omega )$ of $\omega$-weighted *BV*-functions in an open set $\Omega \subset \mathbb {R}^n$, where the weight $\omega :\Omega \rightarrow [0,\infty )$ is assumed to be a locally integrable function. We adopt the approach introduced in [5], and further analyzed in [15, 16].

Given a $\omega$-weighted locally integrable function in $\Omega$, $u\in L^1_{\omega ,\text {loc}}(\Omega )$, where

3.2 $$\begin{aligned} \begin{aligned} L^1_{\omega ,\text {loc}}(\Omega )&\!:=\! \bigg \{ v: \Omega \!\rightarrow \!\mathbb {R}\text { measurable:} \!\int _K |v(x)|\, \omega (x)\;\text {d}x<\infty \\  &\quad \text { for all compact }K\subset \Omega \bigg \}, \end{aligned} \end{aligned}$$

we define its $\omega$-weighted total variation in $\Omega ,$ $TV_{\omega }(u,\Omega )$, by

3.3 $$\begin{aligned} \begin{aligned} TV_{\omega }(u,\Omega ):= \sup \bigg \{ \int _\Omega u\,{{\,\textrm{div}\,}}\varphi \;\text {d}x&: \, \varphi \in {{\,\textrm{Lip}\,}}_c(\Omega ;\mathbb {R}^2), \\  &\quad |\varphi | \leqslant \omega \bigg \} \end{aligned} \end{aligned}$$

(see also Sect. 2). Accordingly, we define the space $BV_{\omega }(\Omega )$ of $\omega$-weighted *BV*-functions in $\Omega$ by

$$\begin{aligned} \begin{aligned} BV_{\omega }(\Omega ):=\big \{ u\in L^1_\omega (\Omega )\!:\, TV_{\omega }(u,\Omega )<\infty \big \}, \end{aligned} \end{aligned}$$

endowed with the semi-norm

3.4 $$\begin{aligned} \begin{aligned}&\Vert u\Vert _{BV_{\omega }(\Omega )}:=\Vert u\Vert _{L^1_{\omega }(\Omega )} +TV_{\omega }(u,\Omega ), \\&\quad \quad \text {where } \Vert u\Vert _{L^1_{\omega }(\Omega )}:= \int _\Omega |u(x)|\, \omega (x)\;\text {d}x. \end{aligned} \end{aligned}$$

Clearly, if $\omega \equiv 1$, then we recover the usual space $BV$ of functions of bounded variation. Moreover, if $\omega >0$ (Lebesgue)-a.e. in $\Omega$ and $\omega$ belongs to the global Muckenhoupt class $A_1$, meaning that there is $c>0$ such that for (Lebesgue)-a.e.  $x\in \Omega$ and for every ball $B(x,r)\subset \Omega$, we have

3.5 $$\begin{aligned} \begin{aligned} \omega (x)\geqslant c[\omega ]_{B(x,r)}, \end{aligned} \end{aligned}$$

then expression in (3.4) defines a norm in $BV_{\omega }(\Omega )$. Next, we collect some properties of $BV_{\omega }(\Omega )$, proved in [5, 15, 16], that will be used in our analysis.

#### Theorem 3.1

Let $\Omega \subset \mathbb {R}^n$ be an open set and let $\omega :\Omega \rightarrow [0,\infty )$ be a locally integrable function. Then, the following hold:

(i) The map $u\mapsto TV_{\omega }(u,\Omega )$ is lower-semicontinuous with respect to the (strong) convergence in $L^1_{\omega ,\text {loc}}(\Omega )$.
(ii) Given $u\in L^1_{\omega ,\text {loc}}(\Omega )$, we have that $TV_{\omega }(u,\Omega )=TV_{\omega ^{sc^-}}(u,\Omega )$, where $\omega ^{sc^-}$ denotes the lower-semicontinuous envelope of $\omega$.
(iii) Assume that $\omega$ is lower-semicontinuous and strictly positive everywhere in $\Omega$. Then, we have that $u\in L^1_{\text {loc}}(\Omega )$ and $TV_{\omega }(u,\Omega )<\infty$ if and only if $u\in BV_\text {loc}(\Omega )$ and $\omega \in L^1(\Omega ;\vert Du\vert )$. If any of these two equivalent conditions hold, then we have
  $$\begin{aligned} \begin{aligned} TV_{\omega }(u,B)= \int _B \omega (x)\;\text {d}|Du|(x) \end{aligned} \end{aligned}$$
  for every Borel set $B\subset \Omega$.

#### Proof

The proof of (*i*)–(*iii*) may be found in [5] under the additional assumption that $\omega$ satisfies a Muckenhoupt $A_1$ condition in (3.5) (see [5] for the details). Without assuming this extra assumption on $\omega$, the proof of (*i*) may be found in [15, Proposition 1.3.1 and Remark 1.3.2]; the proof of (*ii*) follows from [15, Proposition 2.1.1 and Theorem 2.1.2]; finally, (*iii*) is shown in [15, Theorem 2.1.5]. $\square$

The existence and uniqueness of solutions of Level 2 of the learning scheme $({\mathscr {L}}\!{\mathscr {S}})_{{TV\!}_\omega }$ in (1.4) with (1.5) replaced by (1.11) are hinged on the following theorem.

#### Theorem 3.2

Let $v\in L^2(\Omega )$ and let $\omega :\Omega \rightarrow (0,\infty )$ be an $L^\infty$ function with $0<{{\,\mathrm{ess\,inf}\,}}_\Omega \omega \leqslant {{\,\mathrm{ess\,sup}\,}}_\Omega \omega <\infty$. Then, there exists a unique $\bar{u} \in BV_{\omega }(\Omega )$ satisfying

$$\begin{aligned} \begin{aligned}&\int _\Omega |v-\bar{u}|^2\;\text {d}x +TV_{\omega }(\bar{u},\Omega )\\&\quad =\min _{u\in BV_{\omega }(\Omega )}\bigg \{\int _\Omega |v-u|^2\;\text {d}x +TV_{\omega }(u,\Omega ) \bigg \}. \end{aligned} \end{aligned}$$

Moreover, denoting by $\omega ^{sc^-}$ the lower-semicontinuous envelope of $\omega$, we have $\bar{u}\in BV_{\omega }(\Omega )\cap BV(\Omega ) \cap BV_{\omega ^{sc^-}}(\Omega )$ and

$$\begin{aligned} \begin{aligned} TV_{\omega }(\bar{u},\Omega )= \int _\Omega \omega ^{sc^-}(x)\;\text {d}|D\bar{u}|(x). \end{aligned} \end{aligned}$$

#### Proof

For $$u\in BV_{\omega }(\Omega )$$, set

$$\begin{aligned} \begin{aligned} E[u]:= \int _\Omega |v-u|^2\;\text {d}x +TV_{\omega }(u,\Omega ), \end{aligned} \end{aligned}$$

and let

$$\begin{aligned} \begin{aligned} {\mathcalligra{m}}:= \inf _{u\in BV_{\omega }(\Omega )} E[u]. \end{aligned} \end{aligned}$$

Note that $0\leqslant {\mathcalligra{m}} \leqslant E[0] = \Vert v\Vert _{L^2(\Omega )}^2$, and consider $(u_n)_{n\in \mathbb {N}} \subset BV_{\omega }(\Omega )$ such that

3.6 $$\begin{aligned} \begin{aligned} {\mathcalligra{m}} = \lim _{n\rightarrow \infty } E[u_n]. \end{aligned} \end{aligned}$$

By hypothesis, there exist $c_1$, $c_2\in \mathbb {R}^+$ such that for a.e. $x\in \Omega$, we have

3.7 $$\begin{aligned} \begin{aligned} c_1 \leqslant \omega (x) \leqslant c_2. \end{aligned} \end{aligned}$$

Consequently, for all $x\in \Omega$,

3.8 $$\begin{aligned} \begin{aligned} c_1 \leqslant \omega ^{sc^-}(x) \leqslant c_2. \end{aligned} \end{aligned}$$

Then, in view of (3.6) and Theorem 3.1 (*ii*)–(*iii*), for all $n\in \mathbb {N}$ sufficiently large, we have

$$\begin{aligned} \begin{aligned} {\mathcalligra{m}} + 1&\geqslant \int _\Omega |v-u_n|^2\;\text {d}x +TV_{\omega }(u_n,\Omega ) \\&= \int _\Omega |v-u_n|^2\;\text {d}x +TV_{\omega ^{sc^-}}(u_n,\Omega )\\&= \int _\Omega |v-u_n|^2\;\text {d}x + \int _\Omega \omega ^{sc^-}(x)\;\text {d}|D u_n|(x) \\&\geqslant \int _\Omega |v-u_n|^2\;\text {d}x + c_1|Du_n|(\Omega ). \end{aligned} \end{aligned}$$

Thus, extracting a subsequence if necessary (not relabeled), there exists $\bar{u}\in BV(\Omega )$ such that

$$\begin{aligned} \begin{aligned} u_n \overset{*}{\rightharpoonup }\bar{u} \text { in } BV(\Omega ), \quad u_n \rightharpoonup \bar{u} \text { in } L^2(\Omega ), \quad u_n \rightarrow \bar{u} \text { in } L^1(\Omega ). \end{aligned} \end{aligned}$$

Moreover, by (3.7)–(3.8) and Theorem 3.1, we have also $\bar{u}\in BV(\Omega ) \cap BV_{\omega ^{sc^-}}(\Omega )$, with

$$\begin{aligned} \begin{aligned} TV_{\omega }(\bar{u},\Omega )= \int _\Omega \omega ^{sc^-}(x)\;\text {d}|D\bar{u}|(x), \end{aligned} \end{aligned}$$

and

$$\begin{aligned} \begin{aligned} {\mathcalligra{m}}&\leqslant E[\bar{u}] = \int _\Omega |v-\bar{u}|^2\;\text {d}x +TV_{\omega }(\bar{u},\Omega ) \\  &\leqslant \liminf _{n\rightarrow \infty } \bigg ( \int _\Omega |v-u_n|^2\;\text {d}x +TV_{\omega ^{sc^-}}(u_n,\Omega ) \bigg )\\  &=\lim _{n\rightarrow \infty } E[u_n]={\mathcalligra{m}}. \end{aligned} \end{aligned}$$

Because $|\cdot |^2$ is strictly convex, $\bar{u}$ is the unique minimizer of $E[\cdot ]$ over $BV_{\omega }(\Omega )$. $\square$

#### Corollary 3.3

There exists a unique solution $u_\mathscr {L}\in BV_{\omega _\mathscr {L}} (\Omega )\cap BV(\Omega ) \cap BV_{\omega ^{sc^-}_\mathscr {L}}(\Omega )$ to Level 2 of the learning scheme $({\mathscr {L}}\!{\mathscr {S}})_{{TV\!}_\omega }$ in (1.4) with (1.5) replaced by (1.11), where $\omega _\mathscr {L}^{sc^-}$ denotes the lower-semicontinuous envelope of $\omega _\mathscr {L}$. Moreover,

$$\begin{aligned} \begin{aligned}&\min \bigg \{\int _Q|u_\eta -u|^2\;\text {d}x +TV_{\omega _\mathscr {L}}(u,Q)\!:\,u\in BV_{\omega _{\mathscr {L}}}(Q)\bigg \}\\&\quad =\int _Q|u_\eta -u_\mathscr {L}|^2\;\text {d}x + \int _\Omega \omega ^{sc^-}_\mathscr {L}(x)\;\text {d}|Du_\mathscr {L}|(x). \end{aligned} \end{aligned}$$

#### Proof

Using the analysis in Subsect. 3.1, the function $\omega _\mathscr {L}$ in (1.8) satisfies the bounds $c_0 \leqslant \omega _\mathscr {L}\leqslant \tfrac{1}{c_0}$ in $Q$, which, together with Theorem 3.2, concludes the proof. $\square$

#### Remark 3.4

Recalling once again the analysis in Subsect. 3.1, the previous corollary still holds if we assume that (3.1) holds for all $L\in \mathscr {L}$ instead of replacing (1.5) by (1.11).

### On Level 1

Here, we prove that Level 1 of the learning scheme $({\mathscr {L}}\!{\mathscr {S}})_{{TV\!}_\omega }$ admits a solution provided we consider a stopping criterion as in Definition 1.2. We start by checking that the box constraint (1.10) yields such a stopping criterion, after which we establish the converse statement. We then explore alternative stopping criteria.

To prove that the box constraint (1.10) yields a stopping criterion for the refinement of the admissible partitions, we first recall the existence of a smallness condition on the tuning parameter under which the restored image given by the TV model is constant.

#### Proposition 3.5

There exists a positive constant, $C_Q$, depending only on $Q$, such that for any dyadic cube $L\subset Q$ and for all $\alpha \geqslant C_Q \Vert u_\eta \Vert _{L^2(L)}$, the solution $u_{\alpha ,L}$ of (1.6) is constant, with $u_{\alpha ,L} \equiv [ u_\eta ]_L$.

#### Proof

The proof is a simple consequence of [47, Proposition 2.5.7] combined with the scaling invariance of the constant in the 2-dimensional Poincaré–Wirtinger inequality in $BV$ (see [2, Remark 3.50]). $\square$

#### Theorem 3.6

Consider the learning scheme $({\mathscr {L}}\!{\mathscr {S}})_{{TV\!}_\omega }$ in (1.4) with (1.5) replaced by (1.11). Then, there exist $\kappa \in \mathbb {N}$ and $\mathscr {L}_1,..., \mathscr {L}_\kappa \in \mathscr {P}$ such that

3.9 $$\begin{aligned} \begin{aligned}&{\text {argmin}}\left\{ \int _Q|u_c-u_{\mathscr {L}}|^2\;\text {d}x:\,\mathscr {L}\in \mathscr {P}\right\} \\&\quad = {\text {argmin}}\left\{ \int _Q|u_c-u_{\mathscr {L}_i}|^2\;\text {d}x:\,i\in \{1,...,\kappa \}\right\} . \end{aligned} \end{aligned}$$

#### Proof

We use Proposition 3.5 to prove that if a partition contains dyadic squares of side length smaller than a certain threshold, then it can be replaced by a partition of dyadic squares of side length greater than that threshold without changing the minimizer at Level 2.

Let $\bar{\epsilon }\in (0,1)$ be such that for every measurable set $E\subset Q$ with $|E|\leqslant \bar{\epsilon }$, we have

3.10 $$\begin{aligned} \begin{aligned} \Vert u_\eta \Vert _{L^2(E)} \leqslant \frac{c_0}{C_Q}, \end{aligned} \end{aligned}$$

where $c_0$ is the constant in (1.11) and $C_Q$ is the constant given by Proposition 3.5. Set

$$\begin{aligned} \begin{aligned}&\bar{k}:= \min \Big \{k\in \mathbb {N}\!: \, \frac{1}{4^{k}}\leqslant \bar{\epsilon }\Big \}\quad \text {and} \\&\quad \bar{\mathscr {P}} := \Big \{\mathscr {L}\in \mathscr {P}\!:\, |L|\geqslant \frac{1}{4^{\bar{k}}} \text { for all }L\in \mathscr {L}\Big \}. \end{aligned} \end{aligned}$$

Note that $\bar{\mathscr {P}}$ has finite cardinality. Finally, define

$$\begin{aligned} \mathscr {P}^*:= \mathscr {P} \setminus \bar{\mathscr {P}}. \end{aligned}$$

**Fig. 1:**
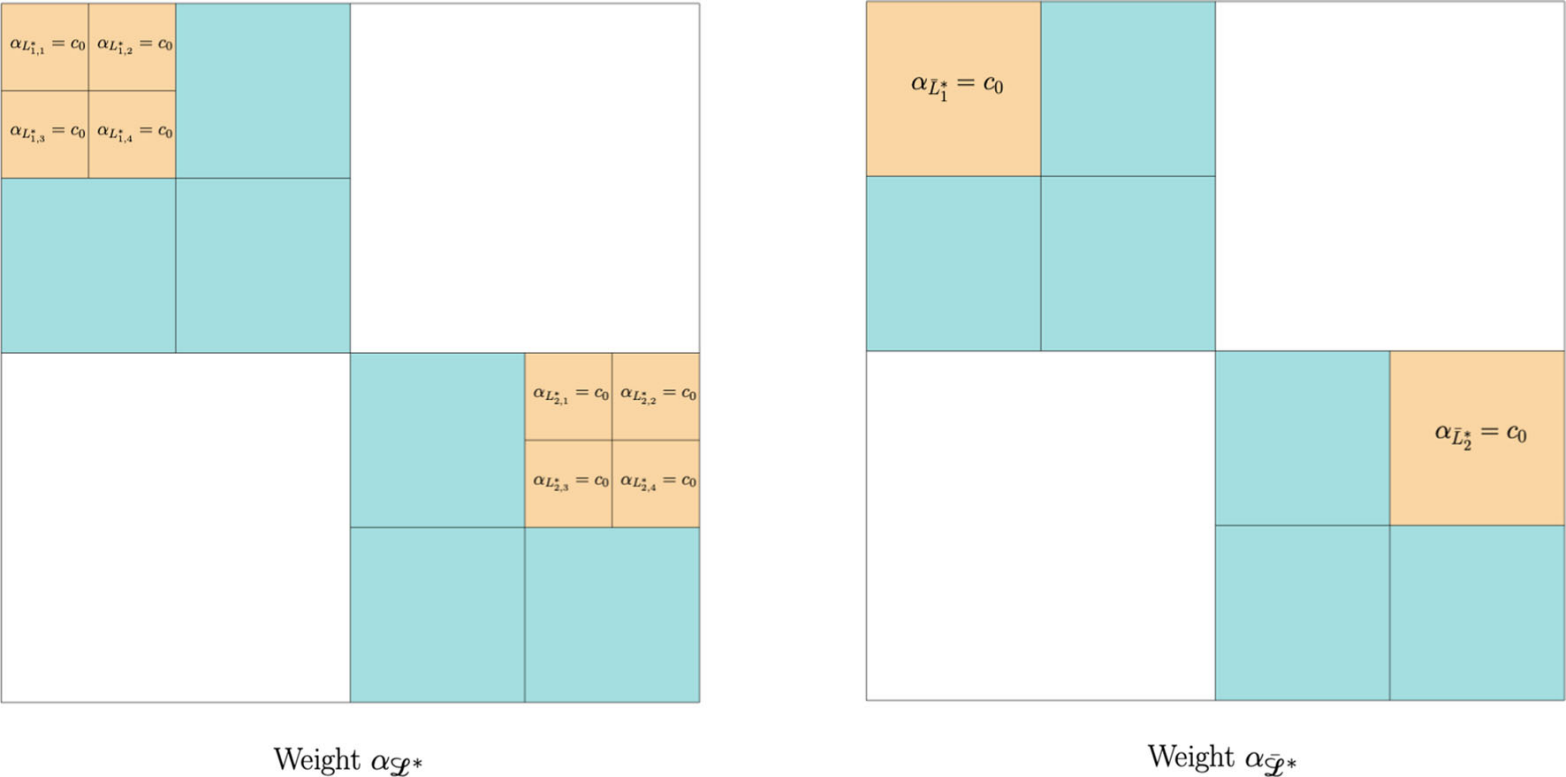
Example of two partitions, $\mathscr {L}^*$ and $\bar{\mathscr {L}}^*$, that yield the same solution at Level 2

Fix $\mathscr {L}^*\in \mathscr {P}^*$, and let

$$\begin{aligned} \begin{aligned} \mathscr {L}^*_-:=\{L^*\in \mathscr {L}^*\!:\, |{\tilde{L}}^*|\geqslant |L^*| \text { for all }{\tilde{L}}^*\in \mathscr {L}^*\} \end{aligned} \end{aligned}$$

be the collection of all dyadic squares with the smallest side length in $\mathscr {L}^*$. Then, there exists $k^*\in \mathbb {N}$, with $k^*> \bar{k}$, such that $|L^*| = \frac{1}{4^{k^*}}$ for all $L^*\in \mathscr {L}^*_-$. Moreover, by construction of our admissible partitions, we can writewhere, for each $j\in \{1,...,\ell \}$,Note that $k^*-1\geqslant \bar{k}$. Then, for any $\alpha \in [c_0,1/c_0]$, Proposition 3.5 and (3.10) yield

$$\begin{aligned} \begin{aligned}&\int _{L^*_{j,i}} |u_c- u_{\alpha ,L^*_{j,i}}|\;\text {d}x = \int _{L^*_{j,i}} |u_c- [ u_\eta ]_{L^*_{j,i}}|\;\text {d}x \quad \text {and} \quad \\&\int _{\bar{L}^*_{j}} |u_c- u_{\alpha ,\bar{L}^*_{j}}|\;\text {d}x = \int _{\bar{L}^*_{j}} |u_c- [ u_\eta ]_{\bar{L}^*_{j}}|\;\text {d}x \end{aligned} \end{aligned}$$

for all $j\in \{1,...,\ell \}$ and $i\in \{1,...,4\}$. Thus, by (1.11),

$$\begin{aligned} \begin{aligned} \alpha _{L^*_{j,i}} = \alpha _{\bar{L}^*_{j}}=c_0 \end{aligned} \end{aligned}$$

for all $j\in \{1,...,\ell \}$ and $i\in \{1,...,4\}$. Consequently (see Fig. 1), definingwe have $\bar{\mathscr {L}^*} \in \mathscr {P}$ and, recalling Level 2,

$$\begin{aligned} \begin{aligned} \omega _{\bar{\mathscr {L}^*}} \equiv \omega _{{\mathscr {L}^*}} \quad \text {and} \quad u_{\omega _{\bar{\mathscr {L}^*}}} \equiv u_{\omega _{{\mathscr {L}^*}}}. \end{aligned} \end{aligned}$$

Note also that $|\bar{L}^*|\geqslant \frac{1}{4^{k^*-1}}$ for all $\bar{L}^*\in \bar{\mathscr {L}^*}$. If $k^*-1 = \bar{k}$, we conclude that $\bar{\mathscr {L}^*} \in \bar{\mathscr {P}}$. Otherwise, if $k^*-1 > \bar{k}$, we repeat the construction above $k^*-1-\bar{k}$ times to obtain a partition $\hat{\mathscr {L}}^* \in \bar{\mathscr {P}}$ for which

$$\begin{aligned}u_{\omega _{\hat{\mathscr {L}}^*}} \equiv u_{\omega _{{\mathscr {L}^*}}}.\end{aligned}$$

Repeating this argument for each $\mathscr {L}^*\in \mathscr {P}^*$, and recalling that $\bar{\mathscr {P}}$ has finite cardinality, we deduce (3.9). $\square$

#### Remark 3.7

We have shown in the previous proof that the box-constraint condition yields a threshold on the minimum side length of the dyadic squares of the possible optimal partitions $\mathscr {L}$ of $Q$. In other words, the box-constraint condition yields the following stopping criterion for the refinement of the admissible partitions:

$(\mathscr {S})$ There exists $\kappa \in \mathbb {N}$ such that $|L|\geqslant \frac{1}{4^\kappa }$ for all $L\in \mathscr {L}$.

In the next subsection, we establish the converse of this implication (see the proof of Theorem 1.4).

We conclude this section by proving Theorem 1.5 that shows the existence of an optimal solution to the learning scheme $({\mathscr {L}}\!{\mathscr {S}})_{{TV\!}_\omega }$.

#### Proof of Theorem 1.5

This result is an immediate consequence of the results of Subsect. 3.1, Corollary 3.3, and Theorem 3.6.$\square$

### Stopping Criteria and Box Constraint

In this subsection, we provide different examples of stopping criteria for the refinement of the admissible partitions, which notion was introduced in Definition 1.2, and we prove Theorem 1.4. The latter is based on the following theorem that yields a natural box constraint for the optimal parameter $\alpha$ associated with the TV model, provided the training data satisfy some mild conditions. The proof of (3.11) in Theorem 3.8 uses arguments from [31] that are alternative to those in [34].

#### Theorem 3.8

Let $\Omega \subset \mathbb {R}^2$ be a bounded, Lipschitz domain and, for each $\alpha \in (0,+\infty )$, let $u_\alpha \in BV(\Omega )$ be given by (1.6) with $L$ replaced by $\Omega$. Assume that the two following conditions on the training data hold:

*i)*: $$TV(u_c,\Omega ) < TV(u_\eta ,\Omega )$$;
*ii)*: $$ \Vert u_\eta - u_c\Vert ^2_{L^2(\Omega )} <\Vert [ u_\eta ]_\Omega - u_c\Vert ^2_{L^2(\Omega )} $$.

Then, there exists $\alpha ^*_\Omega \in (0,+\infty )$ such that

3.11 $$\begin{aligned} \begin{aligned} I( \alpha ^*_\Omega )&=\min _{\alpha \in (0,+\infty )} I(\alpha ), \quad \hbox { where } \\  &I(\alpha ):=\int _\Omega |u_c-u_\alpha |^2\;\text {d}x \text { for } \alpha \in (0,+\infty ). \end{aligned} \end{aligned}$$

Moreover, there exist positive constants $c_\Omega$ and $C_\Omega$, such that any minimizer, $\alpha ^*_\Omega$, of $I$ over $(0,+\infty )$ satisfies $c_\Omega \leqslant \alpha ^*_\Omega < C_\Omega \Vert u_\eta \Vert _{L^2(\Omega )}$. Furthermore, if $\Omega =L$ with $L\subset Q$ a dyadic square, then there exists a positive constant $c_L$ such that any minimizer, $\alpha ^*_L$ of $I$ over $(0,+\infty )$ satisfies $c_L\leqslant \alpha ^*_L < C_Q\Vert u_\eta \Vert _{L^2(L)}$, where $C_Q$ is the constant given by Proposition 3.5. In particular, $\alpha ^*_L\rightarrow 0$ as $|L|\rightarrow 0$.

#### Remark 3.9

The constants $C_\Omega$ and $C_Q$ characterizing the upper bound for the optimal parameters in Theorem 3.8 depend only on the domains, $\Omega$ and $Q$, respectively (cf. Proposition 3.5). On the other hand, the constants $c_\Omega$ and $c_L$ providing a lower bound depend not only on the corresponding domain, but also on $u_c$ and $u_\eta$.

The proof of Theorem 3.8 is hinged on the next lemma of continuity with respect to the parameter in the ROF functional, including the limit cases where the parameter vanishes or tends to $+\infty$.

#### Lemma 3.10

Let $\Omega \subset \mathbb {R}^2$ be a bounded, Lipschitz domain and, for each $\alpha \in (0,+\infty )$, let $u_\alpha \in BV(\Omega )$ be given by (1.6) with $L$ replaced by $\Omega$. Consider the family of functionals $(F_{\bar{\alpha }})_{\bar{\alpha }\in [0,+\infty ]}$, where $F_{\bar{\alpha }}:L^2(\Omega )\rightarrow [0,+\infty ]$ is defined by

$$\begin{aligned}&F_\alpha [u]:={\left\{ \begin{array}{ll} \int _{\Omega }|u_\eta -u|^2\;\text {d}x +\alpha TV(u,\Omega )~\text {if } u\in BV(\Omega ),\\ +\infty \qquad \qquad \text {otherwise,} \end{array}\right. }\quad \\  &\text { for } \bar{\alpha }=\alpha \in (0,+\infty ),\\&F_0 [u]:= \int _{\Omega }|u_\eta -u|^2\;\text {d}x \text { for } \bar{\alpha }=0,\\&F_{\infty } [u]:={\left\{ \begin{array}{ll} \int _{\Omega }|u_\eta -c|^2\;\text {d}x & \qquad \text {if } u\equiv c\in \mathbb {R}, \\ +\infty & \qquad \text {otherwise,} \end{array}\right. }\quad \\  &\text { for } \bar{\alpha }=+\infty , \end{aligned}$$

and denote by $u_{\bar{\alpha }}:= {\text {argmin}}_{u\in L^2(\Omega )}F_{\bar{\alpha }}[u]$ their unique minimizers, given by

3.12 $$\begin{aligned} \begin{aligned} u_{\bar{\alpha }} = {\left\{ \begin{array}{ll} u_{\alpha } & \text {if } \bar{\alpha }=\alpha ,\\ u_{\eta } & \text {if } \bar{\alpha }=0,\\ {[} u_{\eta }]_{\Omega } & \text {if } \bar{\alpha }=+\infty .\\ \end{array}\right. } \end{aligned} \end{aligned}$$

Let $(\alpha _j)_{j\in \mathbb {N}} \subset (0,+\infty )$ and $\bar{\alpha }\in [0,\infty ]$ be such that $\alpha _j\rightarrow \bar{\alpha }$ in $[0,+\infty ]$. Then, we have that $u_{\alpha _j} \rightarrow u_{\bar{\alpha }}$ strongly in $L^2(\Omega )$.

#### Proof

We treat the cases $\bar{\alpha } \in (0, +\infty )$, $\bar{\alpha } = 0$, and $\bar{\alpha }= +\infty$ separately.

Let us first assume that $\bar{\alpha } \in (0,+\infty )$. The proof of this case essentially follows the computations in [47, Thm. 2.4.20], but since our notation and focus are different, we present a complete proof adapted to our setting. Being $u_{\alpha _j}$ a minimizer of $F_{\alpha _j}[u]$ and $u_{\bar{\alpha }}$ a minimizer of $F_{\bar{\alpha }}[u]$, we get that

$$\begin{aligned} u_\eta - u_{\bar{\alpha }}&= \bar{\alpha }p_{\bar{\alpha }} \quad \text { with } p_{\bar{\alpha }} \in \partial TV[u_{\bar{\alpha }}],\\ u_\eta - u_{\alpha _j}&= \alpha _j p_{\alpha _j} \quad \text { with } p_{\alpha _j} \in \partial TV[u_{\alpha _j}], \end{aligned}$$

where $\partial TV$ denotes the subdifferential in $L^2(\Omega )$ of *TV* (extended to be $+\infty$ on $L^2(\Omega ){\setminus } BV(\Omega )$). Multiplying the first equality by $\alpha _j / \bar{\alpha }$ and subtracting the second one from it, we obtain

$$\begin{aligned} \alpha _j (p_{\bar{\alpha }} - p_{\alpha _j})&= \frac{\alpha _j}{\bar{\alpha }} (u_\eta - u_{\bar{\alpha }}) - (u_\eta - u_{\alpha _j})\\&=\left( \frac{\alpha _j}{\bar{\alpha }}-1\right) (u_\eta - u_{\bar{\alpha }}) + u_{\alpha _j} - u_{\bar{\alpha }}. \end{aligned}$$

Multiplying the preceding identity by $u_{\bar{\alpha }} - u_{\alpha _j}$, integrating over $\Omega$, and using the monotonicity of $\partial TV$, we obtain

$$\begin{aligned} 0 \leqslant \left( \frac{\alpha _j}{\bar{\alpha }}-1\right) \int _\Omega (u_\eta - u_{\bar{\alpha }})(u_{\bar{\alpha }} {-} u_{\alpha _j})\;\text {d}x {-} \Vert u_{\bar{\alpha }} {-} u_{\alpha _j}\Vert ^2_{L^2(\Omega )}. \end{aligned}$$

Consequently, using Cauchy–Schwarz’s inequality, and reorganizing the terms, it follows that

$$\begin{aligned} \Vert u_{\bar{\alpha }} - u_{\alpha _j}\Vert _{L^2(\Omega )} \leqslant \frac{|\alpha _j - \bar{\alpha }|}{\bar{\alpha }} \Vert u_\eta - u_{\bar{\alpha }}\Vert _{L^2(\Omega )}. \end{aligned}$$

On the other hand, taking into account that $u_{\bar{\alpha }} = {\text {argmin}}_{L^2(\Omega )} F_{\bar{\alpha }}$, we have that

$$\begin{aligned} \Vert u_{\bar{\alpha }} - u_\eta \Vert ^2_{L^2(\Omega )}&\leqslant \Vert u_{\bar{\alpha }} - u_\eta \Vert ^2_{L^2(\Omega )} + \bar{\alpha } TV(u_{\bar{\alpha }},\Omega )\\&=F_{\bar{\alpha }}[u_{\bar{\alpha }}] \leqslant F_{\bar{\alpha }}[0] = \Vert u_\eta \Vert ^2_{L^2(\Omega )}, \end{aligned}$$

which, together with the preceding estimate, yields

$$\begin{aligned} \Vert u_{\bar{\alpha }} - u_{\alpha _j}\Vert _{L^2(\Omega )} \leqslant \frac{|\alpha _j - \bar{\alpha }|}{\bar{\alpha }} \Vert u_\eta \Vert _{L^2(\Omega )}. \end{aligned}$$

We now consider the $\bar{\alpha }=0$ case. Because $\Omega$ is a bounded, Lipschitz domain, we can find a sequence $(\hat{u}_\kappa )_{\kappa \in \mathbb {N}}\in C^\infty (\overline{\Omega })\subset BV(\Omega )$ such that $\hat{u}_\kappa \rightarrow u_\eta$ in $L^2(\Omega )$. Since $(\alpha _j)^{-\frac{1}{2}}\rightarrow \infty$, we can modify $(\hat{u}_\kappa )_{\kappa \in \mathbb {N}}$ by repeating each of its elements as (finitely) many times as necessary so that the resulting sequence, denoted by $(u_j)_{j\in \mathbb {N}}$, satisfies $TV(u_j,\Omega )\leqslant (\alpha _j)^{-\frac{1}{2}}$ for all $j\in \mathbb {N}$ large enough. Thus, $u_j\rightarrow u_\eta$ in $L^2(\Omega )$ and $\lim _{j\rightarrow \infty }\alpha _jTV(u_j,\Omega )=0$. Using this sequence in the minimality of $u_{\alpha _j}$ results in

$$\begin{aligned} \Vert u_{\alpha _j}- &   u_\eta \Vert ^2_{L^2(\Omega )} + \alpha _j TV(u_{\alpha _j}) \\  &   \quad \leqslant \Vert u_{j} - u_\eta \Vert ^2_{L^2(\Omega )} + \alpha _j TV(u_{j}), \end{aligned}$$

Because both terms on the right-hand side converge to zero, we conclude that $(u_{\alpha _j})_{j \in \mathbb {N}}$ converges to $u_\eta$ strongly in $L^2(\Omega )$, as well.

We are left to treat the $\bar{\alpha }= +\infty$ case. First, we claim that $[u_{\alpha _j}]_\Omega = [u_{\eta }]_\Omega$ for all $j\in \mathbb {N}$. To see this, we use $u_{\alpha _j} = {\text {argmin}}_{u \in BV(\Omega )} F_{\alpha _j}[u]$ to get for any $c \in \mathbb {R}$ that

$$\begin{aligned}  &   \Vert u_{\alpha _j} - u_\eta \Vert ^2_{L^2(\Omega )} + \alpha _j TV(u_{\alpha _j},\Omega )\\  &   \quad \leqslant \Vert u_{\alpha _j} - u_\eta - c\Vert ^2_{L^2(\Omega )} + \alpha _j TV(u_{\alpha _j},\Omega ). \end{aligned}$$

Thus, $\Vert u_{\alpha _j} - u_\eta \Vert ^2_{L^2(\Omega )} \leqslant \Vert u_{\alpha _j} - u_\eta - c\Vert ^2_{L^2(\Omega )}$. Moreover, we also know that

$$\begin{aligned} [u_{\alpha _j} - u_\eta ]_\Omega = {\text {argmin}}_{c \in \mathbb {R}} \Vert u_{\alpha _j} - u_\eta - c\Vert ^2_{L^2(\Omega )} \end{aligned}$$

with only one minimizer by strict convexity, which would lead to a contradiction with the previous inequality unless $[u_{\alpha _j} - u_\eta ]_\Omega = 0$. In other words, we must have $[u_{\alpha _j}] = [u_\eta ]_\Omega$ for all $j\in \mathbb {N}$. To conclude, we use the estimate $F_{\alpha _j}[u_{\alpha _j}] \leqslant \Vert u_\eta \Vert ^2_{L^2(\Omega )}$ as above, which by the definition of $F_{\alpha _j}$ implies that

$$\begin{aligned} \lim _{j \rightarrow \infty } TV(u_{\alpha _j},\Omega ) = 0. \end{aligned}$$

Moreover, by the Poincaré inequality, we have that

$$\begin{aligned} \Vert u_{\alpha _j} - [u_\eta ]_{\Omega }\Vert _{L^2(\Omega )}  &   = \Vert u_{\alpha _j} - [u_{\alpha _j}]_{\Omega }\Vert _{L^2(\Omega )}\\    &   \leqslant C \, TV(u_{\alpha _j},\Omega ). \end{aligned}$$

Thus, $(u_{\alpha _j})_{j \in \mathbb {N}}$ converges to $[u_\eta ]_{\Omega }$ strongly in $L^2(\Omega )$. $\square$

From the preceding lemma, we immediately deduce the following corollary.

#### Corollary 3.11

Let $\Omega \subset \mathbb {R}^2$ be a bounded, Lipschitz domain, and let $I:(0,+\infty )\rightarrow [0,+\infty )$ be the function defined in (3.11). Then, *I* can be extended continuously to a function $\widehat{I}:[0,+\infty ]\rightarrow [0,+\infty ]$ defined for $\bar{\alpha }\in [0,+\infty ]$ by

3.13 $$\begin{aligned} \begin{aligned} \widehat{I} (\bar{\alpha }) = {\left\{ \begin{array}{ll} I(\alpha )=\Vert u_\alpha - u_c\Vert ^2_{L^2(\Omega )} &  \text {if } \bar{\alpha }= \alpha \in (0,+\infty ),\\ \Vert u_\eta - u_c\Vert ^2_{L^2(\Omega )} &  \text {if } \bar{\alpha }=0,\\ \Vert [ u_\eta ]_\Omega - u_c\Vert ^2_{L^2(\Omega )} &  \text {if } \bar{\alpha }=+\infty . \end{array}\right. } \end{aligned} \end{aligned}$$

#### Remark 3.12

We observe that the only continuity condition on $\widehat{I}$ needed for our analysis to hold is that of lower semicontinuity of $\widehat{I}$, as given by (2.6). However, because it is not hard to prove continuity on the whole of $[0, +\infty ]$ in the TV case, we have done so in the results above, which we believe to be of interest on their own.

#### Proof of Theorem 3.8

We will proceed in three steps.

*Step 1.* We prove that if condition *i)* in the statement holds (i.e., $TV(u_\eta ,\Omega )- TV(u_c,\Omega ) >0$), then there exists $\alpha \in (0,+\infty )$ such that

3.14 $$\begin{aligned} \begin{aligned} \Vert u_\alpha - u_c\Vert ^2_{L^2(\Omega )}<\Vert u_\eta - u_c\Vert ^2_{L^2(\Omega )}. \end{aligned} \end{aligned}$$

To show (3.14), we first recall (see [18]) that for any $\alpha \in (0,+\infty )$, there exists a unique $u_\alpha \in BV(\Omega ) \subset L^2(\Omega )$ such that

3.15 $$\begin{aligned} \begin{aligned} u_\alpha = {\text {argmin}}_{u\in L^1(\Omega )} F_\alpha [u] = {\text {argmin}}_{u\in L^2(\Omega )} F_\alpha [u], \end{aligned} \end{aligned}$$

which allow us to regard $F_\alpha$ as a sum of two convex functionals on $L^2(\Omega )$ with values in $[0,+\infty ]$. Precisely,

$$\begin{aligned} \begin{aligned} F_\alpha [u]= F_\alpha ^1[u] + F_\alpha ^2[u], \end{aligned} \end{aligned}$$

where, for $$u\in L^2(\Omega )$$,

$$\begin{aligned} \begin{aligned}&F_\alpha ^1[u] := \Vert u-u_\eta \Vert ^2_{L^2(\Omega )} \\&\text { and }\\  &F_\alpha ^2 [u]:={\left\{ \begin{array}{ll} \alpha TV(u,\Omega ) & \text {if } u\in BV(\Omega ),\\ +\infty & \text {otherwise.} \end{array}\right. } \end{aligned} \end{aligned}$$

Denoting by $\partial F(v) \in (L^2(\Omega ))'\cong L^2(\Omega )$ the subdifferential of a convex functional $F:L^2(\Omega ) \rightarrow [0,+\infty ]$ at $v\in L^2(\Omega )$, we conclude from (3.15) that

$$\begin{aligned} \begin{aligned} 0\in \partial F_\alpha (u_\alpha ) \hspace{5.0pt}\text { or, equivalently, }\hspace{5.0pt}2(u_\eta - u_\alpha ) \in \partial F_\alpha ^2(u_\alpha ). \end{aligned} \end{aligned}$$

Consequently,

$$\begin{aligned} \begin{aligned} 0&\geqslant F^2_\alpha [ u_\alpha ] - F^2_\alpha [ u_c] +\int _\Omega 2(u_\eta - u_\alpha )(u_c - u_\alpha )\;\text {d}x\\&\geqslant F^2_\alpha [ u_\alpha ]- F^2_\alpha [ u_c]+\int _\Omega 2(u_\eta - u_\alpha )(u_c - u_\alpha )\;\text {d}x \\&\quad -\Vert u_\alpha - u_\eta \Vert ^2_{L^2(\Omega )} \\  &= \alpha \big ( TV(u_\alpha ,\Omega ) - TV(u_c,\Omega )\big )+\Vert u_\alpha - u_c\Vert ^2_{L^2(\Omega )}\\&\quad - \Vert u_\eta - u_c\Vert ^2_{L^2(\Omega )}. \end{aligned} \end{aligned}$$

Hence,

3.16 $$\begin{aligned} \begin{aligned}&\Vert u_\eta - u_c\Vert ^2_{L^2(\Omega )} -\Vert u_\alpha - u_c\Vert ^2_{L^2(\Omega )} \\&\quad \geqslant \alpha \big ( TV(u_\alpha ,\Omega ) - TV(u_c,\Omega )\big ). \end{aligned} \end{aligned}$$

We claim that

3.17 $$\begin{aligned} \begin{aligned} TV(u_\alpha ,\Omega )\nearrow \ TV(u_\eta ,\Omega ) \hspace{5.0pt}\text { as } \hspace{5.0pt}\alpha \searrow 0. \end{aligned} \end{aligned}$$

Assuming that the preceding claim holds, the condition $TV(u_\eta ,\Omega )- TV(u_c,\Omega ) >0$ allows us to find $\tilde{\alpha }\in (0,+\infty )$ for which the left-hand side of (3.16) with $\alpha =\tilde{\alpha }$ is strictly positive. Thus, $\Vert u_\eta - u_c\Vert ^2_{L^2(\Omega )} >\Vert u_{\tilde{\alpha }} - u_c\Vert ^2_{L^2(\Omega )}$, which proves (3.14).

To conclude Step  1, we are left to prove (3.17). Using (3.15), for all $\alpha$, $\beta \in (0,+\infty )$ with $\alpha <\beta$, we have that

$$\begin{aligned} \begin{aligned}&\beta TV(u_\beta ,\Omega ) \leqslant F_\beta [ u_\beta ] \leqslant F_\beta [ u_\eta ]= \beta TV(u_\eta ,\Omega ) \end{aligned} \end{aligned}$$

and

$$\begin{aligned} \begin{aligned}&\Vert u_\alpha -u_\eta \Vert ^2_{L^2(\Omega ) } + \alpha TV(u_\alpha ,\Omega ) \\&\quad \leqslant \Vert u_\beta -u_\eta \Vert ^2_{L^2(\Omega ) } + \alpha TV(u_\beta ,\Omega )\\&\quad {=} \Vert u_\beta -u_\eta \Vert ^2_{L^2(\Omega ) } {+} \beta TV(u_\beta ,\Omega ) + (\alpha - \beta )TV(u_\beta ,\Omega ) \\&\quad {\leqslant }\Vert u_\alpha -u_\eta \Vert ^2_{L^2(\Omega ) } {+} \beta TV(u_\alpha ,\Omega ) + (\alpha - \beta )TV(u_\beta ,\Omega ), \end{aligned} \end{aligned}$$

from which we get that

$$\begin{aligned} \begin{aligned}&\beta TV(u_\beta ,\Omega ) \leqslant \beta TV(u_\eta ,\Omega ) \\&\quad \quad \text {and} \\  &(\alpha - \beta ) TV(u_\alpha ,\Omega ) \leqslant (\alpha - \beta )TV(u_\beta ,\Omega ). \end{aligned} \end{aligned}$$

Hence, recalling that $\beta >0$ and $\alpha -\beta <0$, it follows that $TV(u_\beta ,\Omega )\leqslant TV(u_\eta ,\Omega )$ and $TV(u_\alpha ,\Omega ) \geqslant TV(u_\beta ,\Omega )$. Finally, using the first of these estimates and Lemma 3.10 with an arbitrary decreasing sequence $(\beta _j)_{j\in \mathbb {N}}$ converging to 0, the lower-semicontinuity of the total variation with respect to the strong convergence in $L^1$ yields

$$\begin{aligned} \begin{aligned} TV(u_\eta ,\Omega )&\geqslant \limsup _{j\rightarrow \infty } TV(u_{\beta _j},\Omega )\\&\geqslant \liminf _{j\rightarrow \infty } TV(u_{\beta _j},\Omega )\geqslant TV(u_\eta ,\Omega ). \end{aligned} \end{aligned}$$

This concludes the proof of (3.17).

*Step 2.* We prove that if condition *ii)* in the statement holds, (i.e., $\Vert u_\eta - u_c\Vert ^2_{L^2(\Omega )} <\Vert [ u_\eta ]_\Omega - u_c\Vert ^2_{L^2(\Omega )}$), then there exits $\alpha \in (0,+\infty )$ such that

3.18 $$\begin{aligned} \begin{aligned}&\Vert u_\alpha - u_c\Vert ^2_{L^2(\Omega )}<\Vert [ u_\eta ]_\Omega - u_c\Vert ^2_{L^2(\Omega )}. \end{aligned} \end{aligned}$$

Using Corollary 3.11 with $\bar{\alpha }=0$ together with *ii)*, we obtain

$$\begin{aligned} \begin{aligned}&\limsup _{j\rightarrow \infty } \Vert u_{\alpha _j} - u_c\Vert _{L^2(\Omega )}\\&\quad \leqslant \limsup _{j\rightarrow \infty }\Big ( \Vert u_{\alpha _j} - u_\eta \Vert _{L^2(\Omega )} +\Vert u_\eta - u_c\Vert _{L^2(\Omega )} \Big ) \\&\quad = \Vert u_\eta - u_c\Vert _{L^2(\Omega )} <\Vert [ u_\eta ]_\Omega - u_c\Vert _{L^2(\Omega )}, \end{aligned} \end{aligned}$$

from which (3.18) follows.

*Step 3.* We conclude the proof of Theorem 3.8.

We first show (3.11). Because $\widehat{I}$ is a lower- semicontinuous function on the compact set $[0,+\infty ]$, $\widehat{I}$ attains a minimum on $[0,+\infty ]$. By (3.13), (3.14), and (3.18), we conclude that $\widehat{I}$ attains its minimum at some $\alpha ^*\in (0,+\infty )$. Thus, using (3.13) once more,

3.19 $$\begin{aligned} \begin{aligned} I(\alpha ^*)&=\widehat{I} (\alpha ^*)=\min _{\bar{\alpha } \in [0,+\infty ]} \widehat{I} (\bar{\alpha })\\&=\min _{\alpha \in (0,+\infty )} \widehat{I} (\alpha )=\min _{\alpha \in (0,+\infty )} I(\alpha ), \end{aligned} \end{aligned}$$

which yields (3.11).

Next, to prove the existence of $c_\Omega$ as stated, assume that there exist $(\alpha ^*_j)_{j\in \mathbb {N}}\subset (0,+\infty )$ such that $\alpha ^*_j\rightarrow 0$ and (3.19) holds with $\alpha ^*=\alpha ^*_j$. Then, using the lower semi-continuity of $\widehat{I}$ on $[0,+\infty ]$,

$$\begin{aligned} \begin{aligned} \min _{\bar{\alpha } \in [0,+\infty ]} \widehat{I} (\bar{\alpha }) \leqslant \widehat{I} (0)&\leqslant \liminf _{j\rightarrow \infty } \widehat{I} ( \alpha ^*_j)=\min _{\bar{\alpha } \in [0,+\infty ]} \widehat{I} (\bar{\alpha }), \end{aligned} \end{aligned}$$

which is false by (3.14). This establishes the existence of the constant $c_\Omega$.

On the other hand, as mentioned in the proof of Proposition 3.5, [47, Proposition 2.5.7] yields a positive constant, $C_\Omega$, such that $u_\alpha \equiv [ u_\eta ]_\Omega$ for all $\alpha \geqslant C_\Omega \Vert u_\eta \Vert _{L^2(\Omega )}$. This fact, (3.18), and (3.19) show that we must have $\alpha ^*_\Omega < C_\Omega \Vert u_\eta \Vert _{L^2(\Omega )}$. Finally, the $\Omega =L$ case follows from Proposition 3.5.$\square$

Next, we prove Theorem 1.4.

#### Proof of Theorem 1.4

In view of Theorem 3.6 (also see Remark 3.7), the statement in (a) follows. Conversely, the statement in (b) can be proved arguing as in Subsect. 3.1 and defining

$$\begin{aligned} c_0:= \min \bigg \{\min _{L\in \mathscr {L}\in \bar{\mathscr {P}} } c_L, \big (c_Q\Vert u_\eta \Vert _{L^2(Q)}^2\big )^{-1}\bigg \}, \end{aligned}$$

where $c_L$ and $C_Q$ are the constants given by Theorem 3.8.$\square$

We conclude this section with some examples of stopping criteria for the refinement of the admissible partitions as defined in Definition 1.2.

#### Example 3.13

Here, we give an example of a stopping criterion that, heuristically, means that we only refine a given dyadic square $L$, if the distance of the restored image in $L$ to the clean image is greater than or equal to the sum of the distances of the restored images in each of the subdivisions of $L$ to the clean image, modulo a threshold that is determined by the user.

To make this idea precise, we introduce some notation. Given a dyadic square $$ L^{(1)}\subset Q$$ of side length $$\frac{1}{2^{k+1}}$$, we can find three other dyadic squares, which we denote by $$ L^{(2)}$$, $$L^{(3)}$$, and $$L^{(4)}$$, of side length $$\frac{1}{2^{k+1}}$$ and such that  is a dyadic square of side length $$\frac{1}{2^k}$$. We observe further that $$ L^{(2)}$$, $$ L^{(3)}$$, and $$L^{(4)}$$ are uniquely determined by the requirement that $$L$$ is a dyadic square. Using this notation, and setting $$u_L=u_{\alpha _L}$$ (see (1.5)), we fix $$\delta >0$$ and set up an admissible criteria as follows:3.20As we prove next,

$$\begin{aligned} \begin{aligned} \bar{\mathscr {P}}:=\big \{\mathscr {L}\in \mathscr {P}\!:\, L \text { satisfies }(\mathscr {S}) \text {for all }L\in \mathscr {L}\big \} \end{aligned} \end{aligned}$$

has finite cardinality, which shows that $(\mathscr {S})$ as above provides a stopping criteria for the refinement of the admissible partition.

To show that $$\bar{\mathscr {P}}$$ has finite cardinality, we first observe that if $$L$$ satisfies $$(\mathscr {S})$$, then we can find $$k$$ dyadic squares, $$L_1,..., L_k $$, where $$k\in \mathbb {N}$$ is such that $$|L|=\frac{1}{4^{k}}$$, satisfying

$$\begin{aligned} \begin{aligned} Q=L_1\supset .... \supset L_k \supset L, \,\,|L_k|=\frac{1}{4^{k-1}}, \,\, L_k \text { satisfies }(\mathscr {S}). \end{aligned} \end{aligned}$$

Then, using (3.20), we conclude that

$$\begin{aligned} \begin{aligned} \Vert u_c - u_{Q}\Vert _{L^2(Q)}^2 = \Vert u_c - u_{ L_1}\Vert _{L^2( L_1)}^2 \geqslant c_k + k\delta \end{aligned} \end{aligned}$$

for some positive constant $c_k$, which can only hold true if $k$ is small enough. In other words, there exists $k_\delta \in \mathbb {N}$ such that if $L$ satisfies $(\mathscr {S})$, then $|L|\geqslant \frac{1}{4^{k_\delta }}$. Hence, $\bar{\mathscr {P}}$ has finite cardinality.

## Analysis of the Regularized Weighted-TV and Weighted-Fidelity Learning Schemes $({\mathscr {L}}\!{\mathscr {S}})_{{TV\!}_{\omega _\epsilon }}$ and $({\mathscr {L}}\!{\mathscr {S}})_{{TV-Fid}_\omega }$

The results proved in the preceding section for the weighted-TV learning scheme can be easily adapted to the case of the regularized weighted-TV and the weighted-fidelity learning schemes, $({\mathscr {L}}\!{\mathscr {S}})_{{TV\!}_{\omega _\epsilon }}$ and $({\mathscr {L}}\!{\mathscr {S}})_{{TV-Fid}_\omega }$. For the former, we prove here only Proposition 1.6 and provide an example of a sequence of regularized weights satisfying the conditions assumed in this result. Moreover, we highlight a question that is intimately related to the convergence of the solutions to $({\mathscr {L}}\!{\mathscr {S}})_{{TV\!}_{\omega _\epsilon }}$ as $\epsilon \rightarrow 0^+$ (see Subsect. 4.1). Regarding $({\mathscr {L}}\!{\mathscr {S}})_{{TV-Fid}_\omega }$, and for completeness, we state the analogue existence and equivalence results for the weighted-fidelity learning scheme (see Subsect. 4.2).

### The $({\mathscr {L}}\!{\mathscr {S}})_{{TV\!}_{\omega _\epsilon }}$ Learning Scheme

Next, we prove Proposition 1.6 and provide an example of a sequence $(\omega _\mathscr {L}^\epsilon )_\epsilon$ as in (1.14).

#### Proof of Proposition 1.6

We show that

4.1 $$\begin{aligned} E_\mathscr {L}^\epsilon [u] \leqslant E_\mathscr {L}[u] \end{aligned}$$

for all $u\in L^1(Q)$, from which (1.15) follows.

Let $u\in L^1(Q)$ be such that $E_\mathscr {L}[u]< \infty$. Then, $u\in BV_{\omega _\mathscr {L}}(Q)$ and recalling the definition and properties of the space of weighted $BV$-function discussed in Sect. 3.2, we have that $u\in BV_{\omega _\mathscr {L}^\epsilon }(Q)$ with $TV_{\omega _\mathscr {L}^\epsilon }(u,Q) \leqslant TV_{\omega _\mathscr {L}}(u,Q)$, using the estimate $\omega _\mathscr {L}^\epsilon \leqslant {\omega _\mathscr {L}}$ a.e. in $Q$ in (1.14). Thus, (4.1) holds. $\square$

#### Example 4.1

An example of a sequence $(\omega _\mathscr {L}^\epsilon )_\epsilon$ as in (1.14) can be constructed combining a diagonalization argument with a mollification of a Moreau–Yosida type approximation of $\omega ^{sc^-}_\mathscr {L}$. Precisely, for each $k\in \mathbb {N}$, let $\omega _k:Q\rightarrow (0,\infty )$ be given by

4.2 $$\begin{aligned} \begin{aligned} \omega _k(x):=\inf \big \{\omega ^{sc^-}_\mathscr {L}(y) + k|x-y|\!: \, y\in Q\big \}\quad \text {for }x\in Q. \end{aligned}\nonumber \\ \end{aligned}$$

We recall that each $\omega _k$ is a $k$-Lipschitz function, and we have (see [15, Theorem 2.1.2] for instance)

4.3 $$\begin{aligned} \begin{aligned} \omega _k\nearrow \omega ^{sc^-}_\mathscr {L}\quad \text {pointwise everywhere in }Q. \end{aligned} \end{aligned}$$

Moreover, as we show next,

4.4 $$\begin{aligned} \begin{aligned}&\lim _{k\rightarrow \infty } \Vert \omega _k -\omega ^{sc^-}_\mathscr {L}\Vert _{L^\infty (K)} = 0 \end{aligned} \end{aligned}$$

for any compact set $K$ such that $K\subset {{\,\textrm{int}\,}}(L)$, where $L\in \mathscr {L}$ is arbitrary.

In fact, let $L\in \mathscr {L}$ and let $K$ be a compact set such that $K\subset {{\,\textrm{int}\,}}(L)$. Fix $\tau >0$ and set $\delta :=\frac{{{\,\textrm{dist}\,}}(K,\partial L)}{2}$. Note that $\delta >0$ and

4.5 $$\begin{aligned} \omega ^{sc^-}_\mathscr {L}(x) = \alpha _L\quad \text {for all }x\in {{\,\textrm{int}\,}}(L) \end{aligned}$$

because $\omega _\mathscr {L}(x) = \alpha _L$ for all $x\in L$. Moreover, using (4.2), given $\bar{x}\in K$ we can find $y_k\in Q$ such that

4.6 $$\begin{aligned} \omega _k(\bar{x}) + \tau \geqslant \omega ^{sc^-}_\mathscr {L}( y_k) +\ k|\bar{x}-y_k|. \end{aligned}$$

Hence, using (4.3) and nonnegativity of $\omega ^{sc^-}$, we obtain

$$\begin{aligned} |\bar{x}-y_k|\leqslant \frac{\omega _k(\bar{x}) + \tau - \omega ^{sc^-}_\mathscr {L}(y_k) }{k} \leqslant \frac{\Vert \omega ^{sc^-}_\mathscr {L}\Vert _{L^\infty (Q)} + \tau }{k} <\delta \end{aligned}$$

for all $k\geqslant k_0$ and for some $k_0\in \mathbb {N}$ that is independent of $\bar{x}$. Then, $y_k\in {{\,\textrm{int}\,}}(L)$ for all $k\geqslant k_0$. Consequently, (4.5)–(4.6) then yield

$$\begin{aligned} \omega _k(\bar{x}) + \tau \geqslant \omega ^{sc^-}_\mathscr {L}( y_k) = \alpha _L =\omega ^{sc^-}_\mathscr {L}(\bar{x}) \end{aligned}$$

for all $k\geqslant k_0$. Hence,

$$\begin{aligned} 0\leqslant \omega ^{sc^-}_\mathscr {L}(\bar{x})- \omega _k(\bar{x}) \leqslant \tau \end{aligned}$$

for all $k\geqslant k_0$. Taking the supremum on $\bar{x} \in K$ in the preceding estimate yields (4.4).

On the other hand, for each $k\in \mathbb {N}$, a standard mollification argument yields a sequence $(\omega ^{(k)}_\epsilon )_\epsilon \subset C^\infty (\overline{Q})$ such that

4.7 $$\begin{aligned} \begin{aligned}&\lim _{\epsilon \rightarrow 0^+} \Vert \omega ^{(k)}_\epsilon -\omega _k\Vert _{L^\infty (Q)} = 0. \end{aligned} \end{aligned}$$

Finally, denoting by $Q(x,\delta )$ the open square centered at $x\in \mathbb {R}^2$ and side-length $\delta$, we can write  with ${{\,\textrm{int}\,}}(L_i)=Q(x_i,\delta _i)$, for some $\ell \in \mathbb {N}$, $x_i\in L_i$, and $\delta _i >0$. Then, exploiting the countability of the family4.8and a diagonalization argument together with (4.4) and (4.7), we can find a sequence $(\omega _\mathscr {L}^\epsilon )_\epsilon$ such that

4.9 $$\begin{aligned} \begin{aligned}&\lim _{\epsilon \rightarrow 0^+} \Vert \omega _\mathscr {L}^\epsilon -\omega ^{sc^-}_\mathscr {L}\Vert _{L^\infty (K)} = 0 \end{aligned} \end{aligned}$$

for all compact sets $K\in \mathcal {K}$. From the definition of $\mathcal {K}$ in (4.8), we get that (4.9) also holds for all compact sets $K\subset {{\,\textrm{int}\,}}(L)$ and for any $L\in \mathscr {L}$. Furthermore, using the fact that mollification preserves monotonicity, we deduce from (4.3) and (4.7) that $\omega _\mathscr {L}^\epsilon \nearrow \omega ^{sc^-}_\mathscr {L}$ everywhere in $Q$.

To conclude that (1.14) also holds, it suffices to observe that $\omega ^{sc^-}_\mathscr {L}\leqslant \omega _\mathscr {L}$ in *Q*, $\omega ^{sc^-}_\mathscr {L}\equiv \omega _\mathscr {L}$ in , .

#### Remark 4.2

For fixed $\epsilon$, we can apply the results proved in Sect. 3. In particular, there exists an optimal solution $u^*_\epsilon$ to the learning scheme $({\mathscr {L}}\!{\mathscr {S}})_{{TV\!}_{\omega _\epsilon }}$ in (1.12) with (1.13) replaced by (1.11) (cf. Theorem 1.5).

#### Remark 4.3

An interesting question is whether condition (1.14) yields the convergence

4.10 $$\begin{aligned} \begin{aligned} \lim _{\epsilon \rightarrow 0^+} TV_{\omega _\mathscr {L}^\epsilon }(u,Q) = TV_{\omega _\mathscr {L}}(u,Q) \end{aligned} \end{aligned}$$

for all $u\in BV_{\omega _\mathscr {L}}(Q)$. Because sets of zero Lebesgue measure may not have zero |*Du*| measure, we do not expect (4.10) to hold unless the almost everywhere pointwise convergence in (1.14) is replaced by everywhere pointwise convergence.

To the best of our knowledge, the closest result in this direction is [15, Lemma 2.1.4], which shows the following. If $\tilde{\omega }\geqslant 0$ is lower semi-continuous in $Q$ and $u:Q\rightarrow \mathbb {R}$ is measurable, then we can find a sequence of Lipschitz weights, $(\tilde{\omega }_k^{(u)})_{k\in \mathbb {N}}$, depending on $u$, such that $\tilde{\omega }^{(u)}_k\nearrow \tilde{\omega }$ pointwise everywhere in $Q$ and (4.10) holds (with $\omega _\mathscr {L}^\epsilon$ and $\omega _\mathscr {L}$ replaced by $\tilde{\omega }^{(u)}_k$ and $\tilde{\omega }$, respectively).

### The $({\mathscr {L}}\!{\mathscr {S}})_{{TV-Fid}_\omega }$ Learning Scheme

Given a dyadic square $L\subset Q$ and $\alpha \in (0,\infty )$, we have

$$\begin{aligned} \begin{aligned}&{\text {argmin}}\left\{ \frac{1}{\alpha }\int _{L}|u_\eta -u|^2\;\text {d}x+ TV(u,L)\!:\,u\in BV(L)\right\} \\  &\qquad = {\text {argmin}}\left\{ \int _{L}|u_\eta -u|^2\;\text {d}x+ \alpha TV(u,L)\!:\,u\in BV(L)\right\} . \end{aligned} \end{aligned}$$

Consequently, Proposition 3.5 and Theorem 3.8 remain unchanged if we replace (1.6) by (1.18). These two results are the main tools to prove Theorems 1.4 and 1.5. Using this observation, the arguments used in Sect. 3 can be reproduced here for the weighted-fidelity learning scheme to conclude the two following theorems.

#### Theorem 4.4

(Existence of solutions to $({\mathscr {L}}\!{\mathscr {S}})_{{TV-Fid}_\omega }$) There exists an optimal solution $u^*$ to the learning scheme $({\mathscr {L}}\!{\mathscr {S}})_{{TV-Fid}_\omega }$ in (1.16) with (1.17) replaced by (1.11).

As before, the previous existence theorem holds true under any stopping criterion for the refinement of the admissible partitions provided that the training data satisfies suitable conditions, as stated in the next result.

#### Theorem 4.5

(Equivalence between box constraint and stopping criterion) Consider the learning scheme $({\mathscr {L}}\!{\mathscr {S}})_{{TV-Fid}_\omega }$ in (1.16). The two following conditions hold:

- If we replace (1.17) by (1.11), then there exists a stopping criterion $(\mathscr {S})$ for the refinement of the admissible partitions as in Definition 1.2.
- Assume that there exists a stopping criterion $(\mathscr {S})$ for the refinement of the admissible partitions as in Definition 1.2 such that the training data satisfies for all , with $\bar{\mathscr {P}}$ as in Definition 1.2, the conditions
  (i) $$TV(u_c,L) < TV(u_\eta ,L)$$;
  (ii) $$\displaystyle \Vert u_\eta - u_c\Vert ^2_{L^2(L)} <\Vert [ u_\eta ]_L - u_c\Vert ^2_{L^2(L)} $$.
  Then, there exists $c_0\in \mathbb {R}^+$ such that the optimal solution $u^*$ provided by $({\mathscr {L}}\!{\mathscr {S}})_{{TV-Fid}_\omega }$ with $\mathscr {P}$ replaced by $\bar{\mathscr {P}}$ coincides with the optimal solution $u^*$ provided by $({\mathscr {L}}\!{\mathscr {S}})_{{TV-Fid}_\omega }$ with (1.17) replaced by (1.11).

## Analysis of the Weighted-TGV Learning Scheme $({\mathscr {L}}\!{\mathscr {S}})_{{TGV\!}_\omega }$

This section is devoted to proving the existence of solutions to the training scheme $({\mathscr {L}}\!{\mathscr {S}})_{{TGV\!}_\omega }$ described in (1.20). We begin by providing the precise definition of the quantities $\mathscr {V}_{\omega _{\mathscr {L}}^0}$ and $\mathscr {V}_{\omega _{\mathscr {L}}^1}$ in (1.24), which are particular instances of the general definition of the weighted variation of a Radon measure introduced in Sect. 2 (see (2.4)).

### Definition 5.1

Let $\Omega$ be an open set in $\mathbb {R}^n$ and $\omega :\Omega \rightarrow [0,+\infty )$ a locally integrable function. Given $u\in L^1_{\omega ,\mathrm loc}(\Omega )$ and $v\in L^1_{\omega ,\mathrm loc}(\Omega ;\mathbb {R}^n)$ (see (3.2)), we set

5.1 $$\begin{aligned} \begin{aligned} \mathscr {V}_{\omega }(Du-v,\Omega )&:=\sup \left\{ \int _\Omega (u\, {\text {div}}\,\varphi +v\cdot \varphi )\;\text {d}x\right. \\&\qquad \qquad \left. :\,\varphi \in {\text {Lip}}_c(\Omega ;\mathbb {R}^n),\,|\varphi |\leqslant \omega \right\} \end{aligned} \end{aligned}$$

and

5.2 $$\begin{aligned} \begin{aligned}&\mathscr {V}_{\omega }(\mathcal {E}v,\Omega ):=\sup \left\{ \int _\Omega (v\cdot {\text {div}}\, \xi )\;\text {d}x\right. \\&\left. :\,\xi \in {\text {Lip}}_c(\Omega ;\mathbb {R}^{n\!\times \! n}_{\text {s}ym}),\,|\xi |\!\leqslant \! \omega \right\} , \end{aligned} \end{aligned}$$

where $({\text {div}}\, \xi )_j = \sum _{k=1}^n \frac{\partial \xi _{jk}}{\partial x_k}$ for each $j\in \{1,...,n\}$.

### Remark 5.2

Recalling (2.4), we are using an abuse of notation in the preceding definition as we are not requiring $Du$ nor $\mathcal {E}v$ to be Radon measures. However, if $u\in BV(\Omega )$, then (5.1) is the $\omega$-weighted variation of the Radon measure $Du-v:= Du-v\mathcal {L}^n\lfloor \Omega \in \mathcal {M}(\Omega ;\mathbb {R}^n)$ in the sense of (2.4). Similarly, if $v\in BD(\Omega )$, then (5.2) is the $\omega$-weighted variation of the Radon measure $\mathcal {E}v\in \mathcal {M}(\Omega ;\mathbb {R}^{n\times n}_{\text {s}ym})$ in the sense of (2.4).

Analogously to the $({\mathscr {L}}\!{\mathscr {S}})_{{TV\!}_\omega }$ case, we analyze each level of $({\mathscr {L}}\!{\mathscr {S}})_{{TGV\!}_\omega }$ in a dedicated subsection.

To prove existence of a solution to the learning scheme $({\mathscr {L}}\!{\mathscr {S}})_{{TGV\!}_\omega }$ in (1.20), we argue by a box-constraint approach in which we replace the requirement $\alpha =(\alpha _0,\alpha _1)\in \mathbb {R}^+\times \mathbb {R}^+$ by the stricter condition (1.29). In this case, we replace (1.21) by

5.3 $$\begin{aligned} \bar{\alpha }_L  &   =\inf \Bigg \{{\text {argmin}}\Bigg \{\int _L |u_c- u_{\alpha ,L}|^2\;\text {d}x \nonumber \\  &   \qquad \qquad :\,\alpha \in \big [c_0,\tfrac{1}{c_0}\big ] \times \big [c_1,\tfrac{1}{c_1}\big ]\Bigg \}\Bigg \}. \end{aligned}$$

Throughout this section, for $u\in L^2(\Omega )$, we denote by $\langle u\rangle _\Omega$ the affine projection of *u* given by the unique solution to the minimization problem

5.4 $$\begin{aligned} \begin{aligned} \min \left\{ \int _\Omega |u-v|^2\;\text {d}x:\,v\text { is affine in }\Omega \right\} , \end{aligned} \end{aligned}$$

which will play an analogous role to the average $[u]_\Omega$ in the *TV* case treated in Sect. 3. Note that we have the orthogonality property

5.5 $$\begin{aligned} \int _{\Omega } (u-\langle u\rangle _\Omega )\langle u\rangle _\Omega \;\text {d}x=0 \end{aligned}$$

for every $u\in L^2(\Omega )$, since $\langle u\rangle _\Omega$ is the Hilbert projection of *u* onto a finite dimensional subspace of $L^2(\Omega )$.

### On Level 3

We provide here an analysis of Level 3, and minor variants thereof, of the learning scheme $({\mathscr {L}}\!{\mathscr {S}})_{{TGV\!}_\omega }$ in (1.20).

As in the weighted *TV*-scheme case, the parameter $\alpha _L$ in Level 3 of $({\mathscr {L}}\!{\mathscr {S}})_{{TGV\!}_\omega }$ (see (1.21)) is uniquely determined by definition, and it satisfies $\alpha _L\in [0,+\infty ]^2$. In view of Theorem 5.13 (see Subsect. 5.4), if $L\in \mathscr {L}$ is such that

5.6 $$\begin{aligned} \begin{aligned}&TGV_{\hat{\alpha }_0,\hat{\alpha }_1}(u_c,L)< TGV_{\hat{\alpha }_0,\hat{\alpha }_1}(u_\eta ,L) \\&\quad \quad \text { and }\\  &\quad \Vert u_\eta - u_c\Vert ^2_{L^2(L)} <\Vert \langle u_\eta \rangle _L - u_c\Vert ^2_{L^2(L)} \end{aligned} \end{aligned}$$

for some $\hat{\alpha } = (\hat{\alpha }_0,\hat{\alpha }_1)$, then

$$\begin{aligned} \begin{aligned}&{{\,\textrm{arginf}\,}}\left\{ \int _L |u_c- u_{\alpha ,L}|^2\,\;\text {d}x\!:\,\alpha \in \mathbb {R}^+ \times \mathbb {R}^+\right\} \\  &\quad ={\text {argmin}}\Bigg \{\int _L |u_c- u_{\alpha ,L}|^2\,\;\text {d}x\!:\, \alpha \in \mathbb {R}^+ \times \mathbb {R}^+ \\  &\quad \qquad \qquad \qquad \qquad \quad \text { is s.t. } c_L\leqslant \min \{\alpha _0,\alpha _1\} < C_Q\Vert u_\eta \Vert _{L^2(L)} \Bigg \}, \end{aligned} \end{aligned}$$

where $c_L$ and $C_Q$ are positive constants, with $c_Q$ depending only on $Q$. Furthermore, because each partition $\mathscr {L}\in \mathscr {P}$ is finite, it follows that if (5.6) holds for all $L\in \mathscr {L}$, then

5.7 $$\begin{aligned} \displaystyle \alpha _L\in \Big [\min _{L\in \mathscr {L}} c_L, +\infty \Big ]\times \Big [\min _{L\in \mathscr {L}} c_L, +\infty \Big ] \setminus \{(+\infty ,+ \infty )\}.\nonumber \\ \end{aligned}$$

Moreover, if we consider Level 3 with (1.21) replaced by (5.3), then the minimum

$$\begin{aligned} \begin{aligned} \min _{\alpha \in [c_0,\frac{1}{c_0}] \times [c_1,\frac{1}{c_1}]} \int _L |u_c- u_{\alpha ,L}|^2\,\;\text {d}x \end{aligned} \end{aligned}$$

exists as the minimum of a lower semicontinuous function (see Lemma 5.18 in Subsect. 5.4) on a compact set. In particular, $\bar{\alpha }_L$ in (5.3) is uniquely determined and belongs to the set in (1.29).

### On Level 2

In this subsection, we discuss the existence of solutions to (1.23). In what follows, let $\Omega \subset \mathbb {R}^n$ be an open set and $\omega :\Omega \rightarrow [0,\infty )$ a locally integrable function. Recalling the definition of $L^1_{\omega , \textrm{loc}}(\Omega )$ and $\Vert \cdot \Vert _{L^1_\omega (\Omega )}$ in Subsect. 3.2, as well as (5.2), we define the space $BD_\omega (\Omega )$ of $\omega$-weighted *BD* functions in $\Omega$ by

$$\begin{aligned} BD_\omega (\Omega ):=\big \{v\in L^1_{\omega }(\Omega ;\mathbb {R}^n):\,\mathscr {V}_{\omega }(\mathcal {E}v,\Omega )<\infty \big \}, \end{aligned}$$

and we endow it with the semi-norm

$$\begin{aligned} \Vert v\Vert _{BD_\omega (\Omega )}:=\Vert v\Vert _{L^1_\omega (\Omega ;\mathbb {R}^n)}+\mathscr {V}_\omega (\mathcal {E}v,\Omega ). \end{aligned}$$

Note that if ${{\,\mathrm{ess\,inf}\,}}_\Omega \omega >0$, the semi-norm above is actually a norm, and that $BD_\omega$ with $\omega \equiv 1$ coincides with the classical space of functions with bounded deformation, cf. [62] for instance. The instrumental properties of $BD_\omega$ for our analysis are collected in the ensuing result.

#### Theorem 5.3

Let $\Omega \subset \mathbb {R}^n$ be an open set and $\omega :\Omega \rightarrow [0,\infty )$ a locally integrable function. Then, the following statements hold:

(i) If $\inf _\Omega \omega >0$, then the map $v\mapsto \mathscr {V}_{\omega }(\mathcal {E}v,\Omega )$ is lower-semicontinuous with respect to the (strong) convergence in $L^1_{\omega ,\text {loc}}(\Omega ;\mathbb {R}^n)$.
(ii) Given $v\in L^1_{\omega ,\text {loc}}(\Omega ;\mathbb {R}^n)$, we have $\mathscr {V}_{\omega }(\mathcal {E}v,\Omega )=\mathscr {V}_{\omega ^{sc^-}}(\mathcal {E}v,\Omega )$, where $\omega ^{sc^-}$ denotes the lower-semicontinuous envelope of $\omega$.
(iii) Assume $\omega$ is lower-semicontinuous and strictly positive. Then, we have $v\in L^1_{\text {loc}}(\Omega ;\mathbb {R}^n)$ and $\mathscr {V}_{\omega }(\mathcal {E}v,\Omega )<\infty$ if and only if $v\in BD_{\textrm{loc}}(\Omega )$ and $\omega \in L^1(\Omega ;\vert \mathcal {E}v\vert )$. If any of these two equivalent conditions hold, we have
  $$\begin{aligned} \begin{aligned} \mathscr {V}_{\omega }(\mathcal {E}v,B)= \int _B \omega (x)\;\text {d}|\mathcal {E}v|(x) \end{aligned} \end{aligned}$$
  for every Borel set $B\subset \Omega$.
(iv) If $\omega \in L^\infty _{\text {loc}}(\Omega )$ is lower-semicontinuous and strictly positive, then all bounded sequences in $BD_{\omega }(\Omega )$ are precompact in the strong $L^1_{\omega ,\textrm{loc}}$-topology.

#### Proof

Accounting for the fact that test functions here take values in $\mathbb {R}^{n\times n}_{\textrm{sym}}$, the proof of $(i)$, $(ii)$, and $(iii)$ may be obtained by mimicking that of [15, Proposition 1.3.1], [15, Proposition 2.1.1], and [15, Theorem 2.1.5], respectively.

To prove (*iv*), we observe that for each compact set $K\subset \Omega$, there exists a positive constant $c_K$ such that 0< $\tfrac{1}{c_K} \leqslant \omega \leqslant c_K$ in *K* because $\omega \in L^\infty _{\text {loc}}(\Omega )$ and strictly positive lower-semicontinuous functions are locally bounded away from zero. Then, using (*iii*), we have for every $v\in BD_\omega (\Omega )$ that

$$\begin{aligned}&\mathscr {V}_{\omega }(\mathcal {E}v,K)=\int _K \omega (x)\;\text {d}|\mathcal {E}v|(x) {\left\{ \begin{array}{ll} \geqslant \tfrac{1}{c_K} |\mathcal {E}v|(K),\\ \leqslant c_K |\mathcal {E}v|(K), \end{array}\right. }\\&\Vert v\Vert _{L^1_{\omega }(K;\mathbb {R}^n)}=\int _\Omega |v(x)|\,\omega (x)\;\text {d}x {\left\{ \begin{array}{ll} \geqslant \tfrac{1}{c_K} \Vert v\Vert _{L^1(K;\mathbb {R}^n)},\\ \leqslant c_K \Vert v\Vert _{L^1(K;\mathbb {R}^n)}. \end{array}\right. } \end{aligned}$$

The preceding estimates and the compact embedding of *BD*(*K*) into $L^1(K;\mathbb {R}^n)$ (cf. [62]) yield $(iv)$. $\square$

#### Remark 5.4

If $\omega :\Omega \rightarrow (0,\infty )$ is a lower-semicontinuous function satisfying $0<c\leqslant \inf _\Omega \omega \leqslant \sup _\Omega \omega \leqslant c^{-1}$ for some positive constant $c$, then the arguments in the preceding proof show that Theorem 5.3 $(iv)$ holds globally in $\Omega$. In other words, bounded sequences in $BD_{\omega }(\Omega )$ are precompact in the strong $L^1_{\omega }(\Omega ;\mathbb {R}^n)$-topology.

#### Remark 5.5

Differently from the weighted-TV case (cf. Theorem 3.1), we need the weights $\omega$ in Theorem 5.3 to be bounded from below away from zero for item (*i*) to hold. This is because one cannot resort to arguments based on coarea formulas in the symmetrized gradient case, which prevents us to adapt the arguments in [15, Remark 1.3.2 and Theorem 3.1.13] to this framework.

The next result collects some basic properties of the quantity $\mathscr {V}_{\omega }(Du - v,\Omega )$ given by (5.1).

#### Theorem 5.6

Let $\Omega \subset \mathbb {R}^n$ be an open set and $\omega :\Omega \rightarrow [0,\infty )$ a locally integrable function. Let $u\in BV_\omega (\Omega )$. Then, the following statements hold:

(i) The map $v\rightarrow \mathscr {V}_{\omega }(Du-v,\Omega )$ is lower semicontinuous with respect to the strong convergence in $L^1_{\omega ,\textrm{loc}} (\Omega ;\mathbb {R}^n)$.
(ii) Given $v\in L^1_{\omega ,\text {loc}}(\Omega ;\mathbb {R}^n)$, we have $\mathscr {V}_{\omega }(Du- v,\Omega )=\mathscr {V}_{\omega ^{sc^-}}(Du- v,\Omega )$, where $\omega ^{sc^-}$ denotes the lower-semicontinuous envelope of $\omega$.
(iii) If $v\in L^1_{\omega ,\textrm{loc}} (\Omega ;\mathbb {R}^n)$ and $\omega \in L^1(\Omega ; |Du-v|)$ is lower-semicontinuous and strictly positive, then
  5.8 $$\begin{aligned} \begin{aligned} \mathscr {V}_\omega (Du-v,B)=\int _B \omega (x)\;\text {d}|Du-v|(x) \end{aligned} \end{aligned}$$
  for every Borel set $B\subset \Omega$.

#### Proof

To prove (*i*), let $(v_k)_{k\in \mathbb {N}}\subset L^1_{\omega , \textrm{loc}}(\Omega ;\mathbb {R}^n)$ be a sequence such that $v_k\rightarrow v$ strongly in $L^1_{\omega ,\textrm{loc}}(\Omega ;\mathbb {R}^n)$. Then, by Definition 5.1,

$$\begin{aligned} \mathscr {V}_{\omega }(Du-v_k,\Omega )\geqslant \int _\Omega \big (u\,\textrm{div}\,\varphi +v_k \cdot \varphi \big ) \;\text {d}{x} \end{aligned}$$

for every $\varphi \in \textrm{Lip}_c(\Omega ;\mathbb {R}^n)$ with $|\varphi |\leqslant \omega$ in $\Omega$. Moreover, for all such $\varphi$,

$$\begin{aligned} \begin{aligned} \int _\Omega |v_k {-} v||\varphi |\;\text {d}x\leqslant \int _{{{\,\textrm{supp}\,}}\varphi } |v_k - v|\,\omega \;\text {d}x \rightarrow 0 \text { as } k\rightarrow {+}\infty . \end{aligned} \end{aligned}$$

Hence,

$$\begin{aligned} \liminf _{k\rightarrow +\infty }\mathscr {V}_{\omega }(Du-v_k,\Omega )\geqslant \int _\Omega \big (u\,\textrm{div}\,\varphi +v\cdot \varphi \big ) \;\text {d}{x}, \end{aligned}$$

from which the conclusion follows by taking the supremum over all test functions $\varphi \in \textrm{Lip}_c(\Omega ;\mathbb {R}^n)$ with $|\varphi |\leqslant \omega$ in $\Omega$.

The proof of (*ii*) follows by Definition 5.1, observing that every map $\varphi \in \textrm{Lip}_c(\Omega ;\mathbb {R}^n)$ with $|\varphi |\leqslant \omega$ in $\Omega$ also satisfies $|\varphi |\leqslant (\omega ^{\textrm{sc}})^-$ in $\Omega$.

As we discuss next, the proof of (*iii*) is an adaptation of [15, Theorem 2.1.5]. In fact, because $u\in BV_\omega (\Omega )$ and strictly positive lower-semicontinuous functions are locally bounded away from zero, we have $u\in BV_\textrm{loc}(\Omega )$. Then, for every $\varphi \in \textrm{Lip}_c(\Omega ;\mathbb {R}^n)$ with $|\varphi |\leqslant \omega$ in $\Omega$, we have that

$$\begin{aligned} \int _\Omega \big (u\,\textrm{div}\,\varphi +v\cdot \varphi \big ) \;\text {d}{x}\leqslant \int _\Omega \omega \;\text {d}|Du-v|; \end{aligned}$$

hence, $\mathscr {V}_\omega (Du-v,\Omega )\leqslant \int _\Omega \omega \;\text {d}|Du-v|$. Conversely, since $\omega \in L^1(\Omega ;|Du-v|)$, we infer that

5.9 $$\begin{aligned} \begin{aligned}&\int _{\Omega }\omega \;\text {d}|Du-v|=|\omega (Du-v)|(\Omega )\\&{=}\sup \left\{ \int _\Omega \omega \psi \cdot \;\text {d}(Du-v):\,\psi {\in } \textrm{Lip}_c(\Omega ;\mathbb {R}^n),\,|\psi |{\leqslant } 1\right\} . \end{aligned}\nonumber \\ \end{aligned}$$

Let $(\omega _k)_{k\in \mathbb {N}}$ be an increasing sequence of $k$-Lipschitz functions converging to $\omega$ in $\Omega$ as in Example 4.1 (see also [15, Theorem 2.1.2]). Then, for every $\psi \in \textrm{Lip}_c(\Omega ;\mathbb {R}^n)$ with $|\psi |\leqslant 1$ in $\Omega$, we have $\omega _k \, \psi \in \textrm{Lip}_c(\Omega ;\mathbb {R}^n)$ with $|\omega _k \, \psi |\leqslant \omega _k \leqslant \omega$ in $\Omega$; thus, using the Lebesgue dominated convergence theorem and recalling (5.1), we find that

$$\begin{aligned}&\int _{\Omega } \omega \,\psi \cdot \;\text {d}(Du-v)\\&\quad =\lim _{k\rightarrow \infty } \int _{\Omega } \omega _k\,\psi \cdot \;\text {d}(Du-v)\\&\quad =-\lim _{k\rightarrow \infty }\int _{\Omega } \big (u\,\textrm{div}\,(\omega _k\,\psi )+\omega _k\,\psi \cdot v\big )\,\;\text {d}x \\  &\quad \leqslant \mathscr {V}_\omega (Du-v,\Omega ). \end{aligned}$$

From this estimate and (5.9), we deduce that $\int _\Omega \omega \;\text {d}|Du-v| \leqslant \mathscr {V}_\omega (Du-v,\Omega )$, which concludes the proof of (5.8) when $B=\Omega$. The proof that this identity holds for every Borel set $B\subset \Omega$ can be done exactly as in [15, Theorem 2.1.5]. $\square$

We proceed by showing that the infimum in

5.10 $$\begin{aligned} TGV_{\omega _0,\omega _1}(u,Q):=\inf _{v\in BD_{\omega _1}(Q)}\big \{\mathscr {V}_{\omega _0}  &   (Du-v,Q)\nonumber \\    &   +{\mathscr {V}_{\omega _1}}(\mathcal {E}v,Q)\big \},\nonumber \\ \end{aligned}$$

where $\omega _0,\, \omega _1:Q\rightarrow (0,+\infty )$ are bounded functions and $u \in L^1_{\omega _0}(Q)$, is actually a minimum, and that the contributions due to $\mathscr {V}_{\omega _0}$ and $\mathscr {V}_{\omega _1}$ can be expressed in a simplified way in terms of the lower semicontinuous envelopes of the weights $\omega _0$ and $\omega _1$. We begin with a technical lemma.

#### Lemma 5.7

Let $c_0>0$ be a positive constant. For $i\in \{0,1\},$ let $\omega _i:Q\rightarrow (0,+\infty )$ be such that $c_0<\inf _Q \omega _i<\sup _Q \omega _i <\frac{1}{c_0}$, and let $u\in L^1_{\omega _0,\textrm{loc}}(Q)$. Then, for every $v\in L^1_{\omega _1}(Q;\mathbb {R}^n)$, we have

5.11 $$\begin{aligned} \Vert v\Vert _{L^1_{\omega _1}(Q;\mathbb {R}^n)}\leqslant \frac{1}{c_0^2} \big (\mathscr {V}_{\omega _0}(Du-v,Q)+TV_{\omega _0}(u,Q)\big ).\nonumber \\ \end{aligned}$$

#### Proof

Fix $v\in L^1_{\omega _1}(Q;\mathbb {R}^2)$. Note that the uniform bounds on $\omega _1$ yield

5.12 $$\begin{aligned} \begin{aligned} c_0\int _Q |v(x)| \;\text {d}x&\leqslant \int _Q \omega _1(x) |v(x)| \;\text {d}x = \Vert v\Vert _{L^1_{\omega _1}(Q;\mathbb {R}^n)} \\&\leqslant \frac{1}{c_0} \int _Q |v(x)| \;\text {d}x. \end{aligned}\nonumber \\ \end{aligned}$$

In particular, $v \in L^1(Q;\mathbb {R}^2)$; thus,

5.13 $$\begin{aligned} \begin{aligned}&c_0\int _Q |v(x)| \;\text {d}x \\&= c_0\sup \bigg \{ \int _Q \psi (x) \cdot v(x)\;\text {d}x: \psi \in \textrm{Lip}_{\textrm{c}}(Q;\mathbb {R}^2), \\  &\Vert \psi \Vert _{L^\infty (Q;\mathbb {R}^2)} \leqslant 1 \bigg \} \\&= \sup \bigg \{ \int _Q \tilde{\psi }(x) \cdot v(x)\;\text {d}x: \tilde{\psi }\in \textrm{Lip}_{\textrm{c}}(Q;\mathbb {R}^2), \\  &\Vert \tilde{\psi }\Vert _{L^\infty (Q;\mathbb {R}^2)} \leqslant c_0 \bigg \} \\&\leqslant \sup \bigg \{\int _Q \varphi (x) \cdot v(x)\;\text {d}x: \\  &\varphi \in \textrm{Lip}_{\textrm{c}}(Q;\mathbb {R}^2),\,\, |\varphi |\leqslant \omega _0\bigg \}\\&\leqslant \mathscr {V}_{\omega _0}(Du-v,Q)+TV_{\omega _0}(u,Q), \end{aligned} \end{aligned}$$

where we used Definition 5.1 together with the subadditivity of the supremum in the last estimate, and the bound $c_0 \leqslant \inf _Q \omega _0$ in the preceding one. We then obtain (5.11) by combining (5.12) and (5.13). $\square$

Under the same assumptions of Lemma 5.7, the infimum problem in (1.24) is actually a minimum.

#### Lemma 5.8

Let $c_0>0$ be a positive constant. For $i\in \{0,1\},$ let $\omega _i:Q\rightarrow (0,+\infty )$ be such that $c_0<\inf _Q \omega _i<\sup _Q \omega _i <\frac{1}{c_0}$, and let $u\in L^1(Q)$. Then, there exists $u^*\in BD_{\omega _1}(Q)$ such that

5.14 $$\begin{aligned} \begin{aligned} TGV_{\omega _0,\omega _1}(u,Q)=\mathscr {V}_{\omega _0}(Du-u^*, Q)+\mathscr {V}_{\omega _1}(\mathcal {E}u^*, Q). \end{aligned}\nonumber \\ \end{aligned}$$

#### Proof

We claim that $TGV_{\omega _0,\omega _1}(u,Q)$ is finite if and only if $u\in BV_{\omega _0}(Q)$. In fact, choosing $v=0$ as a competitor in (5.10), we infer that $TGV_{\omega _0,\omega _1}(u,Q)\leqslant TV_{\omega _0}(u,Q)$. On the other hand, recalling  (3.3), we have for any $v\in BD_{\omega _1}(Q)$ that

$$\begin{aligned} TV_{\omega _0}(u,Q)&=\sup \Bigg \{\int _Q (u \, {\text {div}}\,\varphi +v \cdot \varphi -v\cdot \varphi ) \;\text {d}x \\  &:\,\varphi \in {\text {Lip}}_c(\Omega ;\mathbb {R}^2),\,|\varphi |\leqslant \omega _0\Bigg \}\\&\leqslant \mathscr {V}_{\omega _0}(Du-v, Q)+\Vert v\Vert _{L^1_{\omega _0}(Q;\mathbb {R}^2)}\\&\leqslant \mathscr {V}_{\omega _0}(Du-v, Q)+\frac{1}{c_0^2}\Vert v\Vert _{ L^1_{\omega _1}(Q;\mathbb {R}^2)}, \end{aligned}$$

where we used the subadditivity of the supremum combined with Definition 5.1 in the first inequality, and the bounds on the two weights in the second inequality. Thus, $TV_{\omega _0}(u,Q) \leqslant \max \{1, c_0^{-2}\} \, TGV_{\omega _0,\omega _1}(u,Q)$, which concludes the proof of the claim.

To show (5.14), we may assume without loss of generality that $TGV_{\omega _0,\omega _1}(u,Q)<\infty$, in which case $u\in BV_{\omega _0}(Q)$. Moreover, we may find a sequence $(v_n) \subset BD_{\omega _1}(Q)$ such that

5.15 $$\begin{aligned} TGV_{\omega _0,\omega _1}(u,Q)=\lim _{n\rightarrow +\infty }  &   \big \{\mathscr {V}_{\omega _0}(Du-v_n,Q)\nonumber \\  &   \quad +\mathscr {V}_{\omega _1}(\mathcal {E}v_n,Q)\big \} \leqslant C \end{aligned}$$

for some positive constant $C$. From Lemma 5.7 and (5.15) we infer that $\sup _{n\in \mathbb {N}}\Vert v_n\Vert _{BD_{\omega _1}(Q)}<+\infty$. Using the uniform bounds on $\omega _1$, which are inherited by its lower semicontinuous envelope $(\omega _1)^{\text {sc}^-}$, and Theorem 5.3 $(ii)$, also

$$\begin{aligned} \begin{aligned} \sup _{n\in \mathbb {N}}\Vert v_n\Vert _{BD_{(\omega _1)^{\text {sc}^-}}(Q)}<+\infty . \end{aligned} \end{aligned}$$

Moreover, by Theorem 5.3 $(i)$, $(ii)$, and $(iv)$ (also see Remark 5.4), there exists $u^*\in BD_{\omega _1}(Q) \cap BD_{(\omega _1)^{\text {sc}^-}}(Q)$ such that

5.16 $$\begin{aligned} \begin{aligned}&v_n\rightarrow u^*\quad \text {strongly in }L^1_{(\omega _1)^{\text {sc}^-}}(Q;\mathbb {R}^2),\\&\mathscr {V}_{\omega _1}(\mathcal {E}u^*,\Omega )= \mathscr {V}_{(\omega _1)^{\text {sc}^-}}(\mathcal {E}u^*,\Omega )\\&\quad \leqslant \liminf _{n\rightarrow +\infty } \mathscr {V}_{(\omega _1)^{\text {sc}^-}} (\mathcal {E}v_n,\Omega ) = \liminf _{n\rightarrow +\infty }\mathscr {V}_{\omega _1}(\mathcal {E}v_n,\Omega ). \end{aligned}\nonumber \\ \end{aligned}$$

Using the uniform bounds on both weights once more, we also have $v_n \rightarrow u^*$ strongly in $L^1_{\omega _0}(Q;\mathbb {R}^2)$. The minimality of $u^*$ is then a direct consequence of Theorem 5.6 $(i)$, (5.16), and (5.15). $\square$

The next result provides a characterization of the infimum problem in Level 2 of our learning scheme.

#### Proposition 5.9

Let $\phi \in L^2(Q)$, and let $c_0>0$ be a positive constant. For $i\in \{0,1\}$, let $\omega _i:Q\rightarrow [0,+\infty )$ be such that $c_0<\inf _Q \omega _i<\sup _Q \omega _i <\frac{1}{c_0}$. Then, there exists a unique $\bar{u}\in BV_{\omega _0}(Q)$ such that

$$\begin{aligned}&\int _Q |\phi -\bar{u}|\,\;\text {d}x+ TGV_{\omega _0,\omega _1}(\bar{u},Q)\\&\quad =\min _{u\in BV_{\omega _0}(Q)}\left\{ \int _Q |\phi -u|^2\,\;\text {d}x+TGV_{\omega _0,\omega _1}(u,Q)\right\} . \end{aligned}$$

Moreover, denoting by $(\omega _i)^{\text {sc}^-}$ the lower semicontinuous envelope of $\omega _i$, $i\in \{0,1\}$, we have $\bar{u}\in BV(Q)\cap BV_{(\omega _0)^{\text {sc}^-}}(Q)$, and

$$\begin{aligned} TGV_{\omega _0,\omega _1}(\bar{u})&=\int _Q (\omega _0)^{\text {sc}^-}\;\text {d}|D\bar{u}-u^*|+\int _Q (\omega _1)^{\text {sc}^-}\;\text {d}|\mathcal {E}u^*|, \end{aligned}$$

where $u^*\in BD_{\omega _1}(Q)\cap BD_{(\omega _1)^{\text {sc}^-}}(Q)$ is a minimizer of (5.10) associated to $\bar{u}$.

#### Proof

For $u\in BV_{\omega _0}(Q)$, we define

$$\begin{aligned} H[u]:= \int _Q |\phi -u|^2\;\text {d}x+TGV_{\omega _0,\omega _1}(u,Q), \end{aligned}$$

and we set

$$\begin{aligned} \mu :=\inf _{u\in BV_{\omega _0}(Q)}H[u]. \end{aligned}$$

We have $0\leqslant \mu \leqslant F[0]=\Vert \phi \Vert _{L^2(Q)}^2,$ and we may take a sequence $(u_n)_{n\in \mathbb {N}}\subset BV_{\omega _0}(Q)$ such that

$$\begin{aligned} \mu =\lim _{n\rightarrow +\infty }H[u_n]. \end{aligned}$$

Moreover, the boundedness assumptions on the weights $\omega _i$, $i\in \{0,1\}$, yield for all $x\in Q$ that

$$\begin{aligned} c_0\leqslant (\omega _i)^{\textrm{sc}^{-}}(x)\leqslant \frac{1}{c_0}. \end{aligned}$$

Thus, by Lemma 5.8 and Theorems 5.3 and 5.6, we find for all $n\in \mathbb {N}$ large enough that

$$\begin{aligned}&\mu +1\geqslant H[u_n]=\int _Q |\phi -u_n|^2\;\text {d}x+\mathscr {V}_{\omega _0}(Du_n-u_n^*,Q)\\  &\quad +\mathscr {V}_{\omega _1}(\mathcal {E}u_n^*,Q)\\&=\int _Q |\phi -u_n|^2\;\text {d}x+\mathscr {V}_{(\omega _0)^{\textrm{sc}^-}}(Du_n-u_n^*,Q)+\mathscr {V}_{(\omega _1)^{\textrm{sc}^-}}(\mathcal {E}u_n^*,Q)\\&=\int _Q |\phi -u_n|^2\;\text {d}x+\int _Q (\omega _0)^{\textrm{sc}^-}\;\text {d}|Du_n-u_n^*|\\  &~\quad {+}\int _Q (\omega _1)^{\textrm{sc}^{-}}\;\text {d}|\mathcal {E}u_n^*|\\&{\geqslant } \int _Q |\phi {-}u_n|^2\;\text {d}x+{c_0}|Du_n-u_n^*|(Q)+{c_0}|\mathcal {E}u_n^*|(Q). \end{aligned}$$

An argument by contradiction as in the classical *TGV* case and variants thereof (see, e.g., [29, Proposition 5.3]) yields that the sequences $(u_n^*)_{n\in \mathbb {N}}$ and $(u_n)_{n\in \mathbb {N}}$ are uniformly bounded in *BD*(*Q*) and *BV*(*Q*), respectively. Thus, there exist $\bar{u}^*\in BD(Q)$ and $u\in BV(Q)$ such that, up to extracting a not relabelled subsequence,

$$\begin{aligned}&u_n\overset{*}{\rightharpoonup }\bar{u}\quad \text {weakly* in }BV(Q),\\&u_n^*\overset{*}{\rightharpoonup }\bar{u}^*\quad \text {weakly* in }BD(Q). \end{aligned}$$

By the bounds on the weights, and their lower-semicontinuous envelopes, and Theorems 5.3 and 5.6, we deduce that $\bar{u}\in BV_{(\omega _0)^{\textrm{sc}^-}}(Q) \cap BV_{\omega _0}(Q) \cap BV(Q)$ and $\bar{u}^*\in BD_{(\omega _1)^{\textrm{sc}^-}}(Q) \cap BD_{\omega _1}(Q) \cap BD(Q)$, with

5.17 $$\begin{aligned} \mu \leqslant H[\bar{u}]&\leqslant \int _Q |\phi -\bar{u}|^2\;\text {d}x+\mathscr {V}_{(\omega _0)^{\textrm{sc}^-}}(D\bar{u}-\bar{u}^*,Q)\nonumber \\&\quad +\mathscr {V}_{(\omega _1)^{\textrm{sc}^-}}(\mathcal {E}\bar{u}^*,Q) \leqslant \lim _{n\rightarrow +\infty }H[u_n]=\mu . \end{aligned}$$

Because of the strict convexity of the $L^2$-norm, we infer the uniqueness of $\bar{u}$. Finally, by (5.17),

$$\begin{aligned} TGV_{\omega _0,\omega _1}(\bar{u}, Q)&=\mathscr {V}_{(\omega _0)^{\textrm{sc}^-}}(D\bar{u}-\bar{u}^*,Q)\\  &\qquad +\mathscr {V}_{(\omega _1)^{\textrm{sc}^-}}(\mathcal {E}\bar{u}^*,Q). \end{aligned}$$

The last part of the statement is then a consequence of Theorems 5.3 and 5.6. $\square$

### On Level $1$

As we address next, and similarly to the $({\mathscr {L}}\!{\mathscr {S}})_{{TV\!}_{\omega }}$ case, the box constraint provides a stopping criterion for the $TGV$-learning scheme.

To proceed as in Theorem 3.6, we need an analog to Proposition 3.5, which we now prove. Recalling that *L* represents a cell in a dyadic partition of *Q*, we will use the Sobolev inequality in *BV*(*L*) yielding for every $u\in BV(L)$ that

5.18 $$\begin{aligned} \Vert u - [u]_L\Vert _{L^2(L)} \leqslant C^{BV}_Q |Du|(L), \end{aligned}$$

where $[u]_L \in \mathbb {R}$ is the average of *u* in *L*, and the constant $C^{BV}_Q$ depends only on the shape of *Q* because of scale invariance of the embedding $BV$ in $L^2$ in dimension $d=2$. Moreover, we also have for any $w \in BD(L)$ that

5.19 $$\begin{aligned} \Vert w - R_{M_w} - v_w\Vert _{L^2(L)} \leqslant C^{BD}_Q |\mathcal {E}w|(L), \end{aligned}$$

where $v_w \in \mathbb {R}^2$, $M_w$ is a skew-symmetric matrix (that is, with $M^\top + M=0$, the set of which we denote by $\mathbb {R}^{2 \times 2}_{\text {skew}}$), and $R_{M_w}$ denotes the function defined for $M_w \in \mathbb {R}^{2 \times 2}$ by $R_{M_w}(x) = M_w x$.

#### Lemma 5.10

Let $L\subset Q$ be a dyadic square. Then, there is a constant $C_Q^{rot} > 0$ such that for every $u \in BV(L)$ and for every skew-symmetric matrix $M\in \mathbb {R}^{2\times 2}_{\text {skew}}$, we have

5.20 $$\begin{aligned} C_Q^{rot} |Du|(L) \leqslant |Du - R_M|(L). \end{aligned}$$

#### Proof

Suppose that (5.20) does not hold; then, we may find functions $u_n \in BV(L)$ with $|Du_n|(L) = 1$ and skew-symmetric matrices $M_n \in \mathbb {R}^{2\times 2}_{\text {skew}}$ for which

5.21 $$\begin{aligned} \frac{1}{n}=\frac{1}{n}|Du_n|(L) > |Du_n - R_{M_n}|(L). \end{aligned}$$

Then, in particular, $\Vert R_{M_n}\Vert _{L^1(L)}\leqslant 2$; consequently, since $\mathbb {R}^{2 \times 2}_{\text {skew}}$ is a finite-dimensional set, we can assume that $R_{M_n} \rightarrow R_{M_\infty }$ for some skew-symmetric matrix $M_\infty$, up to taking a not relabelled subsequence.

On the other hand, recalling (5.18), there are constants $c_n \in \mathbb {R}$ satisfying

$$\begin{aligned} \Vert u_n - c_n\Vert _{L^2(L)} \leqslant C^{BV}_Q |Du_n|(L); \end{aligned}$$

thus, up to taking a not relabelled further subsequence, we have that $u_n - c_n {\mathop {\rightharpoonup }\limits ^{*}} u_\infty \in BV(L)$ for some $u_\infty \in BV(L)$. Using (5.21) once more, we must have $Du_\infty = R_{M_\infty }$. At this point, we can distinguish two cases, $M_\infty = 0$ or $M_\infty \not =0$.

If $$M_\infty = 0$$, then

$$\begin{aligned} \frac{1}{n}=\frac{1}{n}|Du_n|(L) > |Du_n - R_{M_n}|(L) \rightarrow 1, \end{aligned}$$

which cannot be.

If $M_\infty \ne 0$, then, using the antisymmetry of $DR_{M_\infty }=M_{\infty }$, we again arrive at a contradiction, since

$$\begin{aligned} {{\,\textrm{curl}\,}}Du_\infty = 0 \ \text { but }\ |{{\,\textrm{curl}\,}}R_{M_\infty }| = \sqrt{2} |M_\infty | >0. \end{aligned}$$

To see that the last equality holds, just notice that in the two dimensional case under consideration we must have

$$\begin{aligned}  &   M_\infty = \begin{pmatrix}0 &  a \\ -a &  0\end{pmatrix}\text { for some }a \ne 0, \\  &   \text {which implies } {{\,\textrm{curl}\,}}R_{M_\infty } = -2a. \end{aligned}$$

Thus, we have proved that there is a constant $C_L$, possibly depending on *L*, such that

$$\begin{aligned} C_L |Du|(L) \leqslant |Du - R_M|(L) \quad \text { for all }M \in \mathbb {R}^{2 \times 2}_{\text {skew}}. \end{aligned}$$

To see that $C_L$ is independent of the size of *L*, we just notice that this inequality holds for all *M* and that upon rescaling $x \mapsto r x$ it is enough to replace *M* by *M*/*r* to maintain the inequality. $\square$

The next proposition guarantees that if a dyadic square $L\subset Q$ is small enough, then a solution $u_{\alpha _0,\alpha _1}$ of Level 3 of our $TGV$ learning scheme in (1.20) is affine for every $(\alpha _0,\alpha _1) \in \big [c_0,\frac{1}{c_0}\big ]\times \big [c_1,\frac{1}{c_1}\big ]$. Let us remark that a related result is contained in [57, Proposition 6], which we make quantitative and with a scaling that enables us to draw conclusions on the cell size.

#### Proposition 5.11

Fix $c_0$, $c_1>0$ and $L\subset Q$ a dyadic square. Let $\bar{\alpha }_L$ be the optimal parameter given by (5.3), where $u_{\alpha ,L}$ is defined by (1.22) and (1.19) (with $Q$ replaced by $L$), and let $C_Q^{BV}$, $C_Q^{BD}$, and $C_Q^{rot}$ be the constants in (5.18), (5.19), and (5.20), respectively. If

5.22 $$\begin{aligned} \Vert u_\eta \Vert _{L^2(L)} < \min \left( c_0, \frac{c_1}{C_Q^{BD}|L|^{1/2}}\right) \frac{C_Q^{rot}}{C_Q^{BV}}, \end{aligned}$$

then $\bar{\alpha }_L:=(\overline{\alpha }_0, \overline{\alpha }_1 ) = (c_0, c_1)$ and $u_{\bar{\alpha }_L }:= u_{(\overline{\alpha }_0, \overline{\alpha }_1),L}$ is affine on *L*, with $u_{\bar{\alpha }_L } = \langle u_\eta \rangle _L$.

#### Proof

To simplify the notation in the proof, we omit the dependence of $TGV_{\alpha _0,\alpha _1}$ and $u_{{\alpha }_0, {\alpha }_1}$ on $L$ by writing $TGV_{\alpha _0,\alpha _1}(\cdot )$ in place of $TGV_{\alpha _0,\alpha _1}(\cdot ,L)$ and $u_{({\alpha }_0, {\alpha }_1),L}$, respectively.

Fix $(\alpha _0,\alpha _1) \in \big [c_0,\frac{1}{c_0}\big ]\times \big [c_1,\frac{1}{c_1}\big ]$. The optimality condition for (1.22) reads as

$$\begin{aligned} u_\eta - u_{\alpha _0, \alpha _1} \in \partial TGV_{\alpha _0, \alpha _1}(u_{\alpha _0, \alpha _1}). \end{aligned}$$

Since $TGV_{\alpha _0,\alpha _1}$ is positively one-homogeneous, we have that

$$\begin{aligned}  &   z \in \partial TGV_{\alpha _0,\alpha _1}(u_{\alpha _0, \alpha _1}) \text { if and only if }z \in \partial TGV_{\alpha _0,\alpha _1}(0)\\  &   \text { and }\int _L z \,u_{\alpha _0, \alpha _1} \;\text {d}x = TGV_{\alpha _0, \alpha _1}(u_{\alpha _0, \alpha _1}). \end{aligned}$$

Furthermore, by the definition of subgradient,

$$\begin{aligned}  &   *z \in \partial TGV_{\alpha _0, \alpha _1}(0)\\    &   \text { if and only}\\  &   \text {if} \int _L z\, \overline{u} \;\text {d}x \leqslant TGV_{\alpha _0, \alpha _1}(\overline{u}) \text { for all }\overline{u} \in L^2(L). \end{aligned}$$

Now, given $v \in \mathbb {R}^2$ and $c \in \mathbb {R}$, we denote by $A_{v,c}$ the affine function given by $A_{v,c}(x)=v\cdot x + c$. Because

5.23 $$\begin{aligned} \begin{aligned} TGV_{\alpha _0, \alpha _1}(A_{v,c})=0, \end{aligned} \end{aligned}$$

we deduce from the above with $z=u_\eta - u_{\alpha _0, \alpha _1}$ and $\overline{u}=\pm A_{v,c}$ that $\int _L(u_\eta - u_{\alpha _0, \alpha _1}) A_{v,c} \;\text {d}x=0$ for any $v \in \mathbb {R}^2$ and $c \in \mathbb {R}$; moreover,

$$\begin{aligned} \begin{aligned}&TGV_{\alpha _0,\alpha _1}(u_{\alpha _0, \alpha _1}) \\&= \int _L (u_\eta - u_{\alpha _0, \alpha _1}) u_{\alpha _0, \alpha _1} \;\text {d}x \\  &\quad = \int _L (u_\eta - u_{\alpha _0, \alpha _1}) ( u_{\alpha _0, \alpha _1}\\  &\quad - A_{v,c} ) \;\text {d}x \leqslant \Vert u_\eta - u_{\alpha _0, \alpha _1}\Vert _{L^2(L)} \Vert u_{\alpha _0, \alpha _1} - A_{v,c}\Vert _{L^2(L)}. \end{aligned} \end{aligned}$$

Thus, taking the infimum over $v \in \mathbb {R}^2$ and $c \in \mathbb {R}$ and recalling (5.4), we conclude that

5.24 $$\begin{aligned} \!TGV_{\alpha _0,\alpha _1}(u_{\alpha _0, \alpha _1}) \!\leqslant \! \Vert u_\eta \!-\! u_{\alpha _0, \alpha _1}\Vert _{L^2(L)} \Vert u_{\alpha _0, \alpha _1}\!-\!\langle u_{\alpha _0, \alpha _1}\rangle _L\Vert _{L^2(L)}.\nonumber \\ \end{aligned}$$

On the other hand, since the infimum in the definition of $TGV_{\alpha _0, \alpha _1}$ is attained, there is a $w_u \in BD(L)$ for which

$$\begin{aligned} \begin{aligned}&TGV_{\alpha _0,\alpha _1}(u_{\alpha _0, \alpha _1}) \\&= \inf _{w\in BD(L)} \Big \{\alpha _0 |Du_{\alpha _0, \alpha _1}-w|(L) + \alpha _1 |\mathcal {E}w|(L)\Big \} \\&= \alpha _0 |Du_{\alpha _0, \alpha _1}-w_u|(L) + \alpha _1 |\mathcal {E}w_u|(L)\\&\geqslant \alpha _0 |Du_{\alpha _0, \alpha _1}-w_u|(L) + \frac{\alpha _1}{C^{BD}_Q} \Vert w_u - R_{M_{w_u}} {-} v_{w_u}\Vert _{L^2(L)}, \end{aligned} \end{aligned}$$

where we have used the inequality (5.19) for some skew-symmetric matrix $M_{w_u}\in \mathbb {R}^{2\times 2}$ and vector $v_{w_u}\in \mathbb {R}^2$. Setting $R_u:= R_{M_{w_u}}$ and $v_u:= v_{w_u}$, we get that

$$\begin{aligned} \begin{aligned}&TGV_{\alpha _0,\alpha _1}(u_{\alpha _0, \alpha _1}) \\&\geqslant \alpha _0 |Du_{\alpha _0, \alpha _1}-w_u|(L) + \frac{\alpha _1}{C^{BD}_Q} \Vert w_u - R_u - v_u\Vert _{L^2(L)}\\&\geqslant \alpha _0 |Du_{\alpha _0, \alpha _1}\!-\!w_u|(L) \!+\! \frac{\alpha _1}{C^{BD}_Q |L|^{1/2}} \Vert w_u \!-\! R_u \!-\! v_u\Vert _{L^1(L)} \\&\geqslant \min \left( c_0, \frac{c_1}{C_Q^{BD}|L|^{1/2}}\right) \Big [ |Du_{\alpha _0, \alpha _1}-w_u|(L) \\  &\quad +\Vert w_u - R_u - v_u\Vert _{L^1(L)}\Big ]\\&\geqslant \min \left( c_0, \frac{c_1}{C_Q^{BD}|L|^{1/2}}\right) |Du_{\alpha _0, \alpha _1}-R_u - v_u|(L)\\&= \min \left( c_0, \frac{c_1}{C_Q^{BD}|L|^{1/2}}\right) |D(u_{\alpha _0, \alpha _1}-A_{v_u, 0})-R_u|(L). \end{aligned} \end{aligned}$$

Now, we can apply Lemma 5.10 to $u_{\alpha _0, \alpha _1}-A_{v_u, 0}$ and the Sobolev inequality (5.18) to obtain for some $c_u \in \mathbb {R}$ that

5.25 $$\begin{aligned} \begin{aligned}&TGV_{\alpha _0,\alpha _1}(u_{\alpha _0, \alpha _1}) \\&\geqslant \min \left( c_0, \frac{c_1}{C_Q^{BD}|L|^{1/2}}\right) C^{rot}_Q |D(u_{\alpha _0, \alpha _1}-A_{v_u, 0})|(L)\\&\geqslant \min \left( c_0, \frac{c_1}{C_Q^{BD}|L|^{1/2}}\right) \frac{C^{rot}_Q}{C_Q^{BV}} \Vert u_{\alpha _0, \alpha _1}-A_{v_u, c_u}\Vert _{L^2(L)} \\&\geqslant \min \left( c_0, \frac{c_1}{C_Q^{BD}|L|^{1/2}}\right) \frac{C^{rot}_Q}{C_Q^{BV}} \Vert u_{\alpha _0, \alpha _1}-\langle u_{\alpha _0, \alpha _1}\rangle _L\Vert _{L^2(L)}, \end{aligned}\nonumber \\ \end{aligned}$$

where we used (5.4) once more. Then, if $u_{\alpha _0, \alpha _1}$ was not affine, then $\Vert u_{\alpha _0, \alpha _1}-\langle u_{\alpha _0, \alpha _1}\rangle _L\Vert _{L^2(L)}>0$, so we could combine (5.25) with the upper bound (5.24) and minimality of $u_{\alpha _0, \alpha _1}$ in (1.22) to obtain

$$\begin{aligned}  &   \min \left( c_0, \frac{c_1}{C_Q^{BD}|L|^{1/2}}\right) \frac{C^{rot}_Q}{C_Q^{BV}} \\  &   \quad \leqslant \Vert u_\eta - u_{\alpha _0, \alpha _1}\Vert _{L^2(L)} \leqslant \Vert u_\eta \Vert _{L^2(L)}, \end{aligned}$$

which contradicts (5.22). Thus, $u_{\alpha _0, \alpha _1}$ must be affine.

Finally, using (5.23), (5.4), and $\langle u_\eta \rangle _L$ as a competitor in (1.22), we conclude that $u_{\alpha _0, \alpha _1} =\langle u_{\alpha _0, \alpha _1}\rangle _L = \langle u_\eta \rangle _L$. Hence, $\bar{\alpha }_L = (c_0,c_1)$, and this concludes the proof. $\square$

Owing to Proposition 5.11, we are now in a position to reduce the minimum problem in Level 1 of our training scheme to a minimization over a finite set of admissible partitions.

#### Theorem 5.12

Consider the learning scheme $({\mathscr {L}}\!{\mathscr {S}})_{{TGV\!}_{\omega }}$ in (1.20) with (1.21) restricted by (1.29) (see (5.3)). Then, there exist $\kappa \in \mathbb {N}$ and $\mathscr {L}_1,..., \mathscr {L}_\kappa \in \mathscr {P}$ such that

$$\begin{aligned} \begin{aligned}&{\text {argmin}}\left\{ \int _Q|u_c-u_{\mathscr {L}}|^2\;\text {d}x:\,\mathscr {L}\in \mathscr {P}\right\} \\&\quad = {\text {argmin}}\left\{ \int _Q|u_c-u_{\mathscr {L}_i}|^2\;\text {d}x:\,i\in \{1,...,\kappa \}\right\} . \end{aligned} \end{aligned}$$

#### Proof

The proof is analogous to that of Theorem 3.6, so we only provide a sketch of the argument. The only difference here is that instead of being a constant, the solution $u_{\alpha ,L}$ of Level 1 is affine for any $\alpha :=(\alpha _0,\alpha _1)\in \big [c_0,\frac{1}{c_0}\big ]\times \big [c_1,\frac{1}{c_1}\big ]$ on squares *L* on which (5.22) holds, due to Proposition 5.11. Moreover, $TGV_{\alpha _0,\alpha _1}( u_{\alpha ,L},L)=0$ and, recalling (5.3), the optimal parameter given by (5.3) is $\bar{\alpha }_L=(c_0,c_1)$. As in the proof of Theorem 3.6, this observation allows us to replace any partition $\mathscr {L}^*$ containing such small dyadic squares with another partition $\overline{\mathscr {L}}^*$ whose dyadic squares have all side length above the threshold provided by (5.22) without affecting the minimizer of Level 2. We refer to Fig. 1 for a graphical idea of the argument and to Theorem 3.6 for the details of the proof.$\square$

We conclude this section by proving existence of an optimal solution to the learning scheme $({\mathscr {L}}\!{\mathscr {S}})_{{TGV\!}_\omega }$.

#### Proof of Theorem 1.7

The result follows directly by combining the analysis in Subsect. 5.1, Proposition 5.9, and Theorem  5.12.$\square$

### Stopping Criteria and Box Constraint for TGV

In this subsection, we prove a *TGV*-counterpart to Theorem 3.8. Our result reads as follows.

#### Theorem 5.13

Let $\Omega \subset \mathbb {R}^2$ be a bounded, Lipschitz domain and, for each $\alpha \in (0,+\infty )^2$, let $u_\alpha \in BV(\Omega )$ be given by (1.22) with $L$ replaced by $\Omega$. Assume that the two following conditions on the training data hold:

*i)*: There exists $\hat{\alpha }\in (0,+\infty )^2$ such that $TGV_{\hat{\alpha }_0,\hat{\alpha }_1}(u_c,\Omega ) < TGV_{\hat{\alpha }_0,\hat{\alpha }_1}(u_\eta ,\Omega )$;
*ii)*: $$\displaystyle \Vert u_\eta - u_c\Vert ^2_{L^2(\Omega )} <\Vert \langle u_\eta \rangle - u_c\Vert ^2_{L^2(\Omega )} $$.

Then, there exists

5.26 $$\begin{aligned} \alpha ^*_\Omega \in (0,\!+\!\infty )^2 \cup \big (\{\!+\!\infty \} \times (0, \!+\!\infty )\big ) \cup \big ((0, \!+\!\infty ) \times \{\!+\!\infty \}\big )\nonumber \\ \end{aligned}$$

such that

5.27 $$\begin{aligned} \widehat{J} ( \alpha ^*_\Omega )=\min _{\alpha \in [0,+\infty ]^2} \widehat{J} (\alpha ), \end{aligned}$$

where $\widehat{J}$ is a (lower semicontinuous) extension on $[0, +\infty ]^2$ (see (5.36) in Lemma 5.18) of the function $J:(0,+\infty )^2\rightarrow [0,+\infty )$ defined by

5.28 $$\begin{aligned} J(\alpha ):=\int _\Omega |u_c-u_\alpha |^2\;\text {d}x \quad \text {for } \alpha \in (0,+\infty )^2. \end{aligned}$$

Additionally, there exist positive constants, $c_\Omega$ and $C_\Omega$, such that any minimizer, $\alpha ^*_\Omega$, of $\widehat{J}$ over $[0,+\infty ]^2$ satisfies $c_\Omega \leqslant \min \{(\alpha ^*_\Omega )_0,(\alpha ^*_\Omega )_1\} < C_\Omega \Vert u_\eta \Vert _{L^2(\Omega )}$.

In particular, if $\Omega =L$ with $L\subset Q$ is a dyadic square, then there exists a positive constant, $c_L$, such that any minimizer, $\alpha ^*_L$, of $\widehat{J}$ over $[0,+\infty ]^2$ satisfies $c_L\leqslant \min \{(\alpha ^*_L)_0,(\alpha ^*_L)_1\} < C_Q\Vert u_\eta \Vert _{L^2(L)}$, where $C_Q$ is a constant given by Proposition 5.11.

Owing to the orthogonality property (5.5), condition *ii*) in the statement of the theorem is equivalent to requiring that $\Vert u_c-\langle u_c \rangle -u_\eta +\langle u_\eta \rangle \Vert _{L^2(\Omega )}^2\leqslant \Vert u_c-\langle u_c \rangle \Vert _{L^2(\Omega )}^2.$ In other words, *ii*) is satisfied provided that the perturbation which the noise causes on the non-affine portion of $u_c$ is small in the $L^2$-sense compared to the original non-affine component of $u_c$. This is the case, for example, if $\eta =u_\eta -u_c$ and $\eta -\langle \eta \rangle$ has a small $L^2$-norm, regardless of the $L^2$-norm of $\langle \eta \rangle$.

We remark that the conclusion of the theorem in the general case is slightly weaker than the corresponding result for the *TV*-setting. Indeed, while we can show that both entries of optimal parameters must be uniformly bounded away from zero, we can only prove that their minimum is uniformly bounded from above but cannot prevent that just one of the entries blows up to infinity. This is due to the fact that, without additional conditions, the maps $u_\alpha$ are not necessarily affine if just one of the entries of $\alpha$ becomes infinity, cf. also [57, Proposition 6] for comparison.

However, as a direct consequence of our result, we find a complete characterization for the case in which the analysis of *TGV* reduces to a one-dimensional problem.

#### Corollary 5.14

Under the same assumption and with the same notation of Theorem 5.13, setting $u_{\lambda }:=u_{\lambda (\hat{\alpha }_0,\hat{\alpha }_1)}$ for every $\lambda \in [0,+\infty ]$, there exists $\lambda ^*_\Omega \in (0,+\infty )$ such that

$$\begin{aligned} \begin{aligned} J( \lambda ^*_\Omega (\hat{\alpha }_0,\hat{\alpha }_1))=\min _{\lambda \in (0,+\infty )} J(\lambda (\hat{\alpha }_0,\hat{\alpha }_1)). \end{aligned} \end{aligned}$$

Additionally, there exist positive constants, $c_\Omega$ and $C_\Omega$, such that any minimizer $\lambda ^*_\Omega$ satisfies $c_\Omega \leqslant \lambda ^*_\Omega < C_\Omega \Vert u_\eta \Vert _{L^2(\Omega )}$.

In particular, if $\Omega =L$ with $L\subset Q$ is a dyadic square, then there exists a positive constant, $c_L$, such that any minimizer $\lambda ^*_L$ satisfies $c_L\leqslant \lambda ^*_L < C_Q\Vert u_\eta \Vert _{L^2(L)}$, where $C_Q$ is a constant given by Proposition 5.11.

As in the case of the total variation, we proceed by first studying the limiting behavior of the sum of fidelity and *TGV*-seminorm in the sense of $\Gamma$-convergence. To describe the situation in which the tuning coefficients approach $+\infty$, it is useful to recall that $\mathcal {M}_b(\Omega ;\mathbb {R}^d)$ denotes the set of bounded Radon measures on $\Omega$ with values in $\mathbb {R}^d$ and ${\text {Ker}}\,\mathcal {E}\,(\Omega ;\mathbb {R}^d)$ is the set of all maps $\Phi :\Omega \rightarrow \mathbb {R}^d$ such that $\mathcal {E}\Phi =0$. In particular, $\Phi \in {\text {Ker}}\,\mathcal {E}\,(\Omega ;\mathbb {R}^d)$ if and only if there exists $M\in \mathbb {R}^{d\times d}_{\text {skew}}$ and $m\in \mathbb {R}^d$ such that $\Phi (x)=Mx+m$ for every $x\in \Omega$.

We also recall the function

$$\begin{aligned} m_{\mathcal {E}}:\mathcal {M}_b(\Omega ;\mathbb {R}^d)\rightarrow {\text {Ker}}\,\mathcal {E}\,(\Omega ;\mathbb {R}^d), \end{aligned}$$

introduced in [57, Proposition 3], and defined as the solution to the minimum problem

5.29 $$\begin{aligned} |\mu {-}m_\mathcal {E}(\mu )|(\Omega ){=}\min \{ |\mu {-}\phi |(\Omega ):\,\phi {\in } {\text {Ker}}\,\mathcal {E}\,(\Omega ;\mathbb {R}^d)\},\nonumber \\ \end{aligned}$$

for every $\mu \in \mathcal {M}_b(\Omega ;\mathbb {R}^d)$. Recall that $BH(\Omega )$ denotes the space of functions with bounded Hessian on $\Omega$, namely maps $u\in BV(\Omega )$ such that $D^2 u\in \mathcal {M}_b(\Omega ;\mathbb {R}^{d\times d})$.

#### Lemma 5.15

Let $\Omega \subset \mathbb {R}^2$ be a bounded, Lipschitz domain and, for each $\alpha \in (0,+\infty )^2$, let $u_\alpha \in BV(\Omega )$ be given by (1.22) and (1.19), with $L$ and $Q$ replaced by $\Omega$. Consider the family of functionals $(G_{\bar{\alpha }})_{\bar{\alpha }\in [0,+\infty ]^2}$, where $G_{\bar{\alpha }}:L^1(\Omega )\rightarrow [0,+\infty ]$ is defined by

$$\begin{aligned}&G_\alpha [u]:={\left\{ \begin{array}{ll} \int _{\Omega }|u_\eta -u|^2\;\text {d}x + TGV_{\alpha _0,\alpha _1}(u,\Omega ) & \text {if } u\in BV(\Omega ),\\ +\infty & \text {otherwise,} \end{array}\right. }\quad \text { for } \bar{\alpha }=:\alpha =(\alpha _0,\alpha _1)\in (0,+\infty )^2,\\&G_{0,\bar{\alpha }_1} [u]:={\left\{ \begin{array}{ll} \int _{\Omega }|u_\eta -u|^2\;\text {d}x & \text {if } u\in L^2(\Omega ),\\ +\infty & \text {otherwise,} \end{array}\right. }\quad \text { for } \bar{\alpha }_0=0\text { and }\bar{\alpha }_1\in [0,+\infty ],\\&G_{\infty ,\alpha _1} [u]:={\left\{ \begin{array}{ll} \int _{\Omega }|u_\eta -u|^2\;\text {d}x+\alpha _1|D^2 u|(\Omega ) & \text {if } u\in BH(\Omega ),\\ +\infty & \text {otherwise,} \end{array}\right. }\quad \text { for }\bar{\alpha }_0=+\infty , \bar{\alpha }_1=:\alpha _1\in (0,+\infty ),\\&G_{\bar{\alpha }_0,0} [u]:={\left\{ \begin{array}{ll} \int _{\Omega }|u_\eta -u|^2\;\text {d}x & \text {if } u\in BV(\Omega ),\\ +\infty & \text {otherwise,} \end{array}\right. }\quad \text { for } \bar{\alpha }_0\in (0,+\infty ]\text { and }\bar{\alpha }_1=0,\\&G_{\alpha _0,\infty } [u]:={\left\{ \begin{array}{ll} \int _{\Omega }|u_\eta -u|^2\;\text {d}x +\alpha _0 |Du-m_\mathcal {E}(Du)|(\Omega )& \text {if } u\in BV(\Omega ),\\ +\infty & \text {otherwise,} \end{array}\right. }\quad \text { for }\bar{\alpha }_0=:\alpha _0\in (0,\infty ), \bar{\alpha }_1=+\infty ,\\&G_{\infty ,\infty } [u]:={\left\{ \begin{array}{ll} \int _{\Omega }|u_\eta -u|^2\;\text {d}x & \text {if } Du\in {\text {Ker}}\,\mathcal {E}(\Omega ;\mathbb {R}^d),\\ +\infty & \text {otherwise,} \end{array}\right. }\quad \text { for } \bar{\alpha }_0=\bar{\alpha }_1=+\infty . \end{aligned}$$

Let $(\alpha _j)_{j\in \mathbb {N}} \subset (0,+\infty )^2$ and $\bar{\alpha }\in [0,\infty ]^2$ be such that $\alpha _j\rightarrow \bar{\alpha }$ in $[0,+\infty ]^2$. Then, $(G_{\alpha _j})_{j\in \mathbb {N}}$ $\Gamma$-converges to $G_{\bar{\alpha }}$ in $L^1(\Omega )$.

#### Proof

We first prove that if $(u_j)_{j\in \mathbb {N}}\subset L^1(\Omega )$ and $u\in L^1(\Omega )$ are such that $u_j\rightarrow u$ in $L^1(\Omega )$, then

5.30 $$\begin{aligned} \begin{aligned} G_{\bar{\alpha }} [u] \leqslant \liminf _{j\rightarrow \infty } G_{\alpha _j} [u_j]. \end{aligned} \end{aligned}$$

Without loss of generality, we work under the assumptions that

$$\begin{aligned} \begin{aligned} \liminf _{j\rightarrow \infty } G_{\alpha _j} [u_j]&= \lim _{j\rightarrow \infty } G_{\alpha _j} [u_j]<+\infty \\&\quad \text {and} \quad \\  &\quad \sup _{j\in \mathbb {N}} G_{\alpha _j} [u_j] <+\infty . \end{aligned} \end{aligned}$$

Then, $u_j\in BV(\Omega )$ for all $j\in \mathbb {N}$, $\sup _{j\in \mathbb {N}} \int _{\Omega }|u_\eta -u_j|^2\;\text {d}x <+\infty$ and $\sup _{j\in \mathbb {N}} TGV_{(\alpha _j)_0,(\alpha _j)_1}(u_j,\Omega ) <+\infty$. Hence, $u\in L^2(\Omega )$ and $u_j \rightharpoonup u$ weakly in $L^2(\Omega )$. For each $j\in \mathbb {N}$, let $u^*_j\in BD(\Omega )$ be such that

5.31 $$\begin{aligned} TGV_{(\alpha _j)_0,(\alpha _j)_1}(u_j)\!=\!(\alpha _j)_0|Du_j\!-\!u_j^*|(\Omega )\!+\!(\alpha _j)_1|\mathcal {E}u_j^*|(\Omega ).\nonumber \\ \end{aligned}$$

We now consider each limiting behavior of the sequence $(\alpha _j)_{j\in \mathbb {N}}$ separately.

(i) If $\bar{\alpha }=\alpha \in (0,+\infty )^2$, then an argument by contradiction as the classical *TGV* case and variants thereof (see, e.g., [29, Proposition 5.3]) yields uniform bounds for sequences $(u_j)_{j\in \mathbb {N}}$ and $(u_j^*)_{j\in \mathbb {N}}$ in $BV(\Omega )$ and $BD(\Omega )$, respectively. Thus, $u\in BV(\Omega )$ and $u_j\rightharpoonup u$ weakly-$\star$ in $BV(\Omega )$. Additionally, there exists $u^*\in BD(\Omega )$ such that, up to extracting a further subsequence, $u_j^*\rightharpoonup u^*$ weakly-$\star$ in $BD(\Omega )$, from which (5.30) follows.
(ii) If $\bar{\alpha }_0=0$, then (5.30) holds by the lower-semicontinuity of the $L^2$-norm with respect to the weak convergence in $L^2(\Omega )$.
(iii) If $\bar{\alpha }_0=+\infty$ and $\bar{\alpha }_1\in (0,+\infty )$, then $(u_j^*)_{j\in \mathbb {N}}$ is bounded in $BD(\Omega )$. Thus, there exists $u^*\in BD(\Omega )$ such that, up to extracting a further subsequence, $u_j^*\rightharpoonup u^*$ weakly-$\star$ in $BD(\Omega )$. Additionally, $\lim _{j\rightarrow \infty } |Du_j-u_j^*|(\Omega )=0$. Thus, $u_j\rightarrow u$ strongly in $BV(\Omega )$, $u\in BH(\Omega )$, and (5.30) holds by the lower-semicontinuity of the $L^2$-norm with respect to the strong convergence in $BV(\Omega )$.
(iv) If $\bar{\alpha }_0\in (0,\infty ]$ and $\bar{\alpha }_1=0$, then the situation is analogous to (ii).
(v) If $\bar{\alpha }_1=+\infty$ and $\bar{\alpha }_0\in (0,+\infty )$, then there exists $u^*$ affine and such that $u^*_j\rightarrow u^*$ strongly in $BD(\Omega )$ and $(u_j)_{j\in \mathbb {N}}$ is uniformly bounded in $BV(\Omega )$, so that $u_j\overset{*}{\rightharpoonup }u$ weakly-$\star$ in $BV(\Omega )$. The statement follows from the lower semicontinuity of the total variation with respect to the weak-$\star$ convergence of measures, as well as from (5.29).
(vi) If $\bar{\alpha }_0=\bar{\alpha }_1=+\infty$, then there exists $u^*\in {\text {Ker}}\,\mathcal {E}(\Omega ;\mathbb {R}^d)$ such that $u^*_j\rightarrow u^*$ strongly in $BD(\Omega )$ and $Du_j\rightarrow u^*$ strongly in $\mathcal {M}_b(\Omega ;\mathbb {R}^d)$. Thus, $Du=u^*$ and the statement follows.

Next, we show that for any $u\in L^1(\Omega )$, there exists $(u_j)_{j\in \mathbb {N}}\subset L^1(\Omega )$ such that $u_j\rightarrow u$ in $L^1(\Omega )$ and

5.32 $$\begin{aligned} \begin{aligned} G_{\bar{\alpha }} [u] \geqslant \limsup _{j\rightarrow \infty } G_{\alpha _j} [u_j]. \end{aligned} \end{aligned}$$

Again, we detail the argument in each case separately.

(i) If $\bar{\alpha }=\alpha \in (0,+\infty )^2$, then we can assume, without loss of generality, that $u\in BV(\Omega )$. The conclusion follows then by a classical argument relying on the continuity of *TGV* with respect to its tuning parameters (see, e.g., [29, Theorem 4.2]).
(ii) If $\bar{\alpha }_0=0$, then we consider for every $u\in L^2(\Omega )$ an approximating sequence $(u_k)_{k\in \mathbb {N}}\subset C^\infty _c(\Omega )$ such that $u_k\rightarrow u$ strongly in $L^2(\Omega )$. Choosing the null function as a competitor in the definition of *TGV*, we find that
  $$\begin{aligned} TGV_{(\alpha _j)_0,(\alpha _j)_1}(u_k)\leqslant (\alpha _j)_0 |D u_k|(\Omega ). \end{aligned}$$
  Thus,
  $$\begin{aligned} \lim _{j\rightarrow +\infty }G_{\alpha _j}[u_k] = G_{0,\bar{\alpha }_1}[u_k] \end{aligned}$$
  for every $\bar{\alpha }_1\in [0,+\infty ]$ and every $k\in \mathbb {N}$. The thesis follows then by a classical diagonalization argument.
(iii) If $\bar{\alpha }_0=+\infty \text { and }\bar{\alpha }_1=\alpha _1\in (0,+\infty )$, then we can assume, without loss of generality, that $u\in BH(\Omega )$. In particular, $\nabla u \in BD(\Omega )$ which we can then use as a competitor in the definition of *TGV* to infer that
  $$\begin{aligned} TGV_{(\alpha _j)_0,(\alpha _j)_1}(u)\leqslant (\alpha _j)_1 |D^2u|(\Omega ). \end{aligned}$$
  Thus,
  $$\begin{aligned} \limsup _{j\rightarrow +\infty }G_{\alpha _j}[u]  &   \leqslant \limsup _{j\rightarrow +\infty }\bigg (\int _\Omega |u_\eta -u|^2\;\text {d}x\\    &   \quad +(\alpha _j)_1|D^2 u|(\Omega )\bigg )=G_{\infty ,\alpha _1} [u]. \end{aligned}$$
(iv) If $\bar{\alpha }_0\in (0,+\infty ]$ and $\bar{\alpha }_1=0$, arguing by approximation as in case (ii), we can assume without loss of generality that $u\in C^\infty _c(\Omega )$. Then, choosing $\nabla u$ as a competitor in the definition of *TGV*, we find that
  $$\begin{aligned} TGV_{(\alpha _j)_0,(\alpha _j)_1}(u)\leqslant (\alpha _j)_1 |\nabla ^2 u|(\Omega ). \end{aligned}$$
  Hence, arguing as in case (ii) once more, yields (5.32).
(v) If $\bar{\alpha }_0=\alpha _0\in (0,+\infty )$ and $\bar{\alpha }_1=+\infty$, then we can assume that $u\in BV(\Omega )$. Choosing $m_\mathcal {E}(Du)$ in the definition of *TGV*, yields
  $$\begin{aligned} TGV_{(\alpha _j)_0,(\alpha _j)_1}(u)\leqslant (\alpha _j)_0 |D u-m_\mathcal E(Du)|(\Omega ). \end{aligned}$$
  Hence, arguing as in case (ii), we infer (5.32).
(vi) If $\bar{\alpha }_0=\bar{\alpha }_1=+\infty$, then, without loss of generality, we can assume that $Du\in {\text {Ker}}\,\mathcal {E}(\Omega ;\mathbb {R}^d)$. Choosing *Du* as a competitor in the definition of *TGV* shows that $TGV_{(\alpha _j)_0,(\alpha _j)_1}(u)=0$ for every $j\in \mathbb {N}$, from which (5.32) follows.

The $\Gamma$-convergence of $(G_{\alpha _j})_{j\in \mathbb {N}}$ to $G_{\bar{\alpha }}$ in $L^1(\Omega )$ is then a direct consequence of (5.30) and (5.32). $\square$

As a consequence of the previous result, we provide a characterization of the unique minimizer $u_{\bar{\alpha }}$ of $G_{\bar{\alpha }}$.

#### Corollary 5.16

Under the same assumptions of Lemma 5.15, let $u_{\bar{\alpha }}:={\text {argmin}}_{u\in L^1(\Omega )} G_{\bar{\alpha }}[u]$ for $\bar{\alpha }\in [0,+\infty ]^2$. Then,

5.33 $$\begin{aligned} u_{\bar{\alpha }}={\left\{ \begin{array}{ll} u_{\alpha }& \text {if }\bar{\alpha }=\alpha \in (0,+\infty )^2,\\ u_\eta & \text {if }\bar{\alpha }_0=0\text { or }\bar{\alpha }_1=0,\\ \langle u_\eta \rangle _\Omega & \text {if }\bar{\alpha }_0=\bar{\alpha }_1=+\infty . \end{array}\right. } \end{aligned}$$

Additionally, when just one among $\bar{\alpha }_0$ and $\bar{\alpha }_1$ is infinite, then $\langle u_{\bar{\alpha }}\rangle _\Omega =\langle u_{\eta }\rangle _\Omega$. In these regimes, if additionally $u_\eta =\langle u_{\eta }\rangle _\Omega$, then $u_{\bar{\alpha }}=\langle u_{\bar{\alpha }}\rangle _\Omega$.

#### Proof

The first claim follows directly from Lemma 5.15. We show the second statement only in the case in which $\bar{\alpha }_0=\infty$ and $\bar{\alpha }_1$ is finite, being the case in which $\bar{\alpha }_1=\infty$ analogous. The characterization of minimizers is then a consequence of the orthogonality property in (5.5) which, in turn, yields

$$\begin{aligned} G_{\infty ,\bar{\alpha }_1}(u)  &   =\int _\Omega |\langle u-u_\eta \rangle _\Omega |^2 \;\text {d}x \\  &   \quad +\int _\Omega \left[ \left( u-\langle u\rangle _\Omega \right) -\left( u_\eta -\langle u_\eta \rangle _\Omega \right) \right] ^2 \;\text {d}x\\    &   \quad \, +\, \bar{\alpha }_1| D^2 \left( u-\langle u\rangle _\Omega \right) | \end{aligned}$$

for every $$u\in BH(\Omega )$$. $$\square $$

#### Lemma 5.17

Let $\Omega \subset \mathbb {R}^2$ be a bounded, Lipschitz domain and let $(G_{\bar{\alpha }})_{\bar{\alpha }\in [0,+\infty ]}$ be the family of functionals introduced in Lemma 5.15. Given $\bar{\alpha }\in [0,\infty ]^2$, set $u_{\bar{\alpha }}:= {\text {argmin}}_{u\in L^1(\Omega )} G_{\bar{\alpha }}[u]$. Then, there exists a sequence of pairs of positive numbers, $(\alpha _j)_{j\in \mathbb {N}} \subset (0,+\infty )^2$, such that $\alpha _j\rightarrow \bar{\alpha }$ in $[0,+\infty ]^2$ as $j\rightarrow \infty$ and

5.34 $$\begin{aligned} \begin{aligned} \lim _{j\rightarrow \infty } \int _\Omega |u_{\alpha _j} - u_{\bar{\alpha }}|^2\;\text {d}x=0, \end{aligned} \end{aligned}$$

where $u_{\alpha _j}:= {\text {argmin}}_{u\in L^1(\Omega )} G_{ \alpha _j}[u]$ for all $j\in \mathbb {N}$.

#### Proof

With the same notation as in the proof of Lemma 5.15, we detail the argument for each case separately.

(i) If $\bar{\alpha }=\alpha \in (0,+\infty )^2$, then the statement follows directly by choosing $\alpha _j=\alpha$ for every *j*.
(ii) If $\bar{\alpha }_0=0$, then $u_{\bar{\alpha }}=u_\eta$ and $G_{\bar{\alpha }}[u_{\bar{\alpha }}]=G_{\bar{\alpha }}[u_{\eta }]=0$. In view of Lemma 5.15, there exists a sequence $(u_\eta ^j)_{j\in \mathbb {N}}\subset L^1(\Omega )$ such that
  $$\begin{aligned} \limsup _{j\rightarrow +\infty }G_{\alpha _j}[u^j_\eta ]\leqslant G_{\bar{\alpha }}[u_\eta ]. \end{aligned}$$
  Hence, for any sequence $(\alpha _j)_{j\in \mathbb {N}}\subset (0,+\infty )^2$ satisfying $\alpha _j\rightarrow \bar{\alpha }$, the minimality of $u_{\alpha _j}$ yields
  $$\begin{aligned} \begin{aligned}&\limsup _{j\rightarrow \infty } \bigg ( \int _\Omega |u_{\alpha _j} - u_{\eta }|^2\;\text {d}x+ TGV_{(\alpha _j)_0,(\alpha _j)_1}(u_{\alpha _j},\Omega )\bigg )\\&=\limsup _{j\rightarrow \infty } G_{\alpha _j}[u_{\alpha _j}]\\  &\leqslant \limsup _{j\rightarrow \infty } G_{\alpha _j}[u^j_\eta ]\leqslant G_{\bar{\alpha }}[u_\eta ]=0. \end{aligned} \end{aligned}$$
  Thus, we infer (5.34).
(iii) If $\bar{\alpha }_0=+\infty$ and $\bar{\alpha }_1=\alpha _1\in (0,+\infty )$, then $u_{\bar{\alpha }}\in BH(\Omega )$. For every sequence $(\alpha _0^j)_{j\in \mathbb {N}}$ such that $\alpha _0^j\rightarrow +\infty$ as $j\rightarrow +\infty$, setting $\alpha _j:=(\alpha _0^j,\alpha _1)$, from the minimality of $u_{\alpha _j}$ and choosing $\nabla u_{\bar{\alpha }}$ as a competitor in the definition of *TGV*, we find
  $$\begin{aligned} G_{\alpha _j}[u_{\alpha _j}]&\leqslant G_{\alpha _j}[u_{\bar{\alpha }}]\\&\leqslant \int _\Omega |u_{\bar{\alpha }}-u_\eta |^2\;\text {d}x\\  &+\alpha _1|D^2 u_{\bar{\alpha }}|(\Omega )=G_{\bar{\alpha }}[u_{\bar{\alpha }}]. \end{aligned}$$
  By the fundamental theorem of $\Gamma$-convergence (see [27, Corollary 7.20 and Theorem 7.8]), the equi-coerciveness of the functionals $G_{\alpha _j}$ together with the uniqueness of minimizers yields that $u_{\alpha _j}\rightharpoonup u_{\bar{\alpha }}$ weakly in $L^2(\Omega )$. Property (5.34) follows then by arguing as in item (*iii*) in the first part of the proof of Lemma 5.15 and using the continuous embedding $BV(\Omega ) \subset L^2(\Omega )$.
(iv) If $\bar{\alpha }_0\in (0,+\infty ]$ and $\bar{\alpha }_1=0$, then $u_{\bar{\alpha }}=u_\eta$. Let $(u_\eta ^k)_{k\in \mathbb {N}}\subset C^\infty _c(\Omega )$ be such that $u_\eta ^k\rightarrow u_\eta$ strongly in $L^2(\Omega )$. For every sequence $(\alpha _j)_{j\in \mathbb {N}} \subset (0,+\infty )^2$ satisfying $\alpha _j\rightarrow \bar{\alpha }$, we obtain from the minimality of $u_{\alpha _j}$ that
  $$\begin{aligned} G_{\alpha _j}[u_{\alpha _j}]&\leqslant G_{\alpha _j}[u_\eta ^k]\leqslant \int _\Omega |u_\eta ^k-u_\eta |^2\,\;\text {d}x+(\alpha _1)_j\\  &\quad \int _\Omega |\nabla ^2 u_\eta ^k|\,\;\text {d}x, \end{aligned}$$
  where the latter inequality follows by choosing $\nabla u_\eta ^k$ as a competitor in the definition of *TGV*. Thus,
  $$\begin{aligned} \limsup _{j\rightarrow +\infty }G_{\alpha _j}[u_{\alpha _j}]\leqslant \int _{\Omega }|u_\eta -u_\eta ^k|^2\,\;\text {d}x \end{aligned}$$
  for every $k\in \mathbb {N}$. Passing to the limit as $k\rightarrow +\infty$, we infer that
  $$\begin{aligned} \limsup _{j\rightarrow +\infty }G_{\alpha _j}[u_{\alpha _j}]=0. \end{aligned}$$
  In turn, this implies (5.34).
(v) If $\bar{\alpha }_0=\alpha _0\in (0,+\infty )$ and $\bar{\alpha }_1=+\infty$, then $u_{\bar{\alpha }}\in BV(\Omega )$. For $(\alpha _1^j)_{j\in \mathbb {N}}\subset (0,+\infty )$ such that $\alpha _1^j\rightarrow +\infty$, and setting $\alpha _j:=(\alpha _0,\alpha _1^j)$, we deduce that
  $$\begin{aligned} G_{\alpha _j}[u_{\alpha _j}]&\leqslant G_{\alpha _j}[u_{\bar{\alpha }}]\leqslant \int _{\Omega }|u_{\bar{\alpha }}-u_\eta |^2\;\text {d}x\\  &\quad +\alpha _0|D u_{\bar{\alpha }}-m_{\mathcal {E}}(Du_{\bar{\alpha }})|(\Omega )=G_{\bar{\alpha }}[u_{\bar{\alpha }}]. \end{aligned}$$
  By the fundamental theorem of $\Gamma$-convergence, we infer that $u_{\alpha _j}\rightarrow u_{\bar{\alpha }}$ strongly in $L^1(\Omega )$ and that $G_{\alpha _j}[u_{\alpha _j}]\rightarrow G_{\bar{\alpha }}[u_{\bar{\alpha }}].$ On the other hand, letting $u^*_{\alpha _j}$ be defined as in (5.31) with $u_j$ replaced by $u_{\alpha _j}$, the same argument as in item (*v*) in the first part of the proof of Lemma 5.15 yields $u_{\alpha _j}\overset{*}{\rightharpoonup }u$ weakly-$\star$ in $BV(\Omega )$, and $u^*_{\alpha _j}\rightarrow u^*$ strongly in $BD(\Omega )$ with $u^*$ affine. By combining the above convergences, we deduce
  $$\begin{aligned} G_{\bar{\alpha }}[u_{\bar{\alpha }}]&\leqslant \int _\Omega |u_{\bar{\alpha }}-u_\eta |^2\;\text {d}x\\&+\alpha _0|Du_{\bar{\alpha }}-u^*|(\Omega ) \leqslant \liminf _{j\rightarrow +\infty }\int _\Omega |u_{\alpha _j}-u_\eta |^2\;\text {d}x \\  &\quad +\alpha _0| Du_{\alpha _j}-u^*_{\alpha _j}|(\Omega )\leqslant \lim _{j\rightarrow +\infty }G_{\alpha _j}[u_{ \alpha _j }]\\&=G_{\bar{\alpha }}[u_{\bar{\alpha }}], \end{aligned}$$
  where the first inequality follows by the definition of $m_{\mathcal E}$, cf. (5.29), whereas the second one is a consequence of the lower semicontinuity of the $L^2$-norm with respect to the weak $L^2$-convergence, as well as of the lower semicontinuity of the total variation with respect to the weak-$\star$ convergence of measures.
(vi) If $\bar{\alpha }_0=\bar{\alpha }_1=+\infty$, then $u_{\bar{\alpha }}$ is affine. Thus, for every sequence $(\alpha _j)_{j\in \mathbb {N}} \subset (0,+\infty )^2$ satisfying $\alpha _j\rightarrow \bar{\alpha }$,
  $$\begin{aligned} G_{\alpha _j}[u_{\alpha _j}]\leqslant G_{\alpha _j}[u_{\bar{\alpha }}]=\int _\Omega |u_{\bar{\alpha }}-u_\eta |^2\;\text {d}x=G_{\bar{\alpha }}[u_{\bar{\alpha }}]. \end{aligned}$$
  Property (5.34) is once again obtained arguing by the fundamental theorem of $\Gamma$-convergence, as in (iii).$\square$

In view of the lemmas above, we obtain the following characterization of the lower semicontinuous envelope of *J*.

#### Lemma 5.18

Let $\Omega \subset \mathbb {R}^2$ be a bounded, Lipschitz domain, and let $J:(0,+\infty )^2\rightarrow [0,+\infty )$ be the function defined in (5.28). Then, the extension $\widehat{J}:[0,+\infty ]^2\rightarrow [0,+\infty ]$ of $J$ to the closed interval $[0,+\infty ]^2$ defined for $\bar{\alpha }\in [0,+\infty ]^2$ by

5.35 $$\begin{aligned} \begin{aligned} \widehat{J} (\bar{\alpha }):= \inf \Big \{\liminf _{j\rightarrow \infty } J(\alpha _j)\!&:\, (\alpha _j)_{j\in \mathbb {N}}\subset (0,+\infty )^2, \,\\  &\quad \alpha _j \rightarrow \bar{\alpha } \text { in } [0,+\infty ]^2\Big \}, \end{aligned} \end{aligned}$$

satisfies

5.36 $$\begin{aligned} \begin{aligned} \widehat{J} (\bar{\alpha }) = {\left\{ \begin{array}{ll} J(\alpha )=\Vert u_\alpha - u_c\Vert ^2_{L^2(\Omega )} \,\, \text {if } \bar{\alpha }= \alpha \in (0,+\infty )^2,\\ \Vert u_\eta - u_c\Vert ^2_{L^2(\Omega )} \,\, \text {if } \bar{\alpha }_0=0 \text { or }\bar{\alpha }_1=0,\\ \Vert \langle u_\eta \rangle -u_c\Vert ^2_{L^2(\Omega )} \,\, \text {if }\bar{\alpha }_0=\bar{\alpha }_1=+\infty ,\\ \Vert u_{\bar{\alpha }}-u_c\Vert ^2_{L^2(\Omega )}\text { with }\langle u_{\bar{\alpha }}\rangle = \langle u_{\eta }\rangle \, \text {otherwise}, \end{array}\right. } \end{aligned}\nonumber \\ \end{aligned}$$

where $u_{\bar{\alpha }}$ is the unique minimizer of $G_{\bar{\alpha }}$, cf. Corollary 5.16.

#### Proof

We first note that the function $\widehat{J}$ in (5.35) is lower-semicontinuous on $[0,+\infty ]^2$ and $\widehat{J} \leqslant J$ in $(0,+\infty )^2$. Next, we denote by ${\widetilde{J}}$ the function on $[0,+\infty ]^2$ defined by the right-hand side of (5.36), and observe that

$$\begin{aligned} \begin{aligned} {\widetilde{J}} (\bar{\alpha }) = \Vert u_{\bar{\alpha }} - u_c\Vert ^2_{L^2(\Omega )}, \end{aligned} \end{aligned}$$

where $u_{\bar{\alpha }}:= {\text {argmin}}_{u\in L^1(\Omega )} G_{\bar{\alpha }}(u)$ is given by (5.33). We want to show that $\widehat{J} \equiv {\widetilde{J}}$. By Lemma 5.17, for all $\bar{\alpha }\in [0,+\infty ]^2$ there exists a sequence $(\alpha _j)_{j\in \mathbb {N}}\subset (0,+\infty )$ such that $\alpha _j \rightarrow \bar{\alpha }$ and for which we have

5.37 $$\begin{aligned} \begin{aligned} {\widetilde{J}} (\bar{\alpha })&=\Vert u_{\bar{\alpha }} - u_c\Vert ^2_{L^2(\Omega )} = \lim _{j\rightarrow \infty } \Vert u_{\alpha _j} - u_c\Vert ^2_{L^2(\Omega )} \\  &= \lim _{j\rightarrow \infty } J(\alpha _j). \end{aligned} \end{aligned}$$

Thus, ${\widetilde{J}}(\bar{\alpha })\geqslant \widehat{J} (\bar{\alpha })$ for all $\bar{\alpha }\in [0,+\infty ]^2$. It remains to prove the opposite inequality. For this, we distinguish several cases as in the proofs of Lemma 5.17:

(i) If $\bar{\alpha }=\alpha \in (0,+\infty )^2$, let $(\alpha _j)_{j\in \mathbb {N}}\subset (0,+\infty )$ be any sequence such that $\alpha _j \rightarrow \alpha$. As argued before, we observe that the uniform bounds in $BV(\Omega )$ proved in Lemma 5.15 assert that $(G_{\alpha _j})_{j\in \mathbb {N}}$ is an equi-coercive sequence in $L^1(\Omega )$. Thus, as before, by well-known properties of $\Gamma$-convergence on the convergence of minimizing sequences and energies (see [27, Corollary 7.20 and Theorem 7.8]), together with the uniqueness of minimizers of $G_{\alpha _j}$ and $G_\alpha$, we have that $u_{\alpha _j} \rightharpoonup u_\alpha$ weakly-$\star$ in $BV(\Omega )$ and $\lim _{j\rightarrow \infty } G_{\alpha _j} [u_{\alpha _j}] = G_{\alpha } [u_{\alpha }]$. In particular, $u_{\alpha _j} \rightharpoonup u_{\alpha }$ weakly in $L^2(\Omega )$. Hence,
  $$\begin{aligned} \begin{aligned} {\widetilde{J}}(\alpha )&=\Vert u_{\alpha } - u_c\Vert ^2_{L^2(\Omega )} \leqslant \liminf _{j\rightarrow \infty } \Vert u_{\alpha _j} - u_c\Vert ^2_{L^2(\Omega )}\\  &= \liminf _{j\rightarrow \infty } J(\alpha _j). \end{aligned} \end{aligned}$$
  Taking the infimum of all such sequences $(\alpha _j)_{j\in \mathbb {N}}\subset (0,+\infty )$, we conclude that ${\widetilde{J}}(\alpha )\leqslant \widehat{J} (\alpha )$.
(ii) If $\bar{\alpha }_0 = 0$, we obtain by the corresponding case of Lemma 5.17 that for any sequence $(\alpha _j)_{j\in \mathbb {N}}\subset (0,+\infty )^2$ such that $\alpha _j \rightarrow \bar{\alpha }$, we have
  5.38 $$\begin{aligned} 0 \leqslant \limsup _{j\rightarrow +\infty }G_{\alpha _j}[u_{\alpha _j}]=0,\end{aligned}$$
  which implies $u_{\alpha _j} \rightarrow u_\eta$ strongly in $L^2(\Omega )$, and in turn $\lim _{j \rightarrow \infty } J(\alpha _j) = {\widetilde{J}}(\bar{\alpha })$. Thus, taking the infimum over all such sequences, we conclude that $\widehat{J} (\bar{\alpha }) = \widetilde{J}(\bar{\alpha })$.
(iii) If $\bar{\alpha }_0=+\infty$ and $\bar{\alpha }_1=\alpha _1\in (0,+\infty )$, the thesis follows by observing that the same argument as in (iii) of Lemma 5.17 still holds for any sequence $(\alpha _0^j,\alpha _1^j)_{j\in \mathbb {N}}$ with $\alpha _0^j\rightarrow +\infty$ and $\alpha _1^j\rightarrow \alpha _1$ as $j\rightarrow +\infty$.
(iv) If $\bar{\alpha }_0\in (0,+\infty ]$ and $\bar{\alpha }_1=0$, we can proceed exactly as in (ii) to conclude that for any sequence $(\alpha _j)_{j\in \mathbb {N}}\subset (0,+\infty )^2$ such that $\alpha _j \rightarrow \bar{\alpha }$, we again have (5.38) by the corresponding case of Lemma 5.17.
(v) Analogously to (iii), if $\bar{\alpha }_0=\alpha _0\in (0,+\infty )$ and $\bar{\alpha }_1=+\infty$, the statement is a consequence of the fact that the same argument as in (v) of Lemma 5.17 still holds for any sequence $(\alpha _0^j,\alpha _1^j)_{j\in \mathbb {N}}$ with $\alpha _0^j\rightarrow \alpha _0$ and $\alpha _1^j\rightarrow +\infty$ as $j\rightarrow +\infty$.
(vi) If $\bar{\alpha }_0=\bar{\alpha }_1=+\infty$, by the proof item (vi) of Lemma 5.17, we have for any sequence $(\alpha _j)_{j\in \mathbb {N}}\subset (0,+\infty )^2$ with $\alpha _j \rightarrow \bar{\alpha }$ that $G_{\alpha _j}[u_{\alpha _j}] \leqslant G_{\bar{\alpha }}[u_{\bar{\alpha }}]$, which, analogously to item (iii) of Lemma 5.17, provides that $u_{\alpha _j} \rightharpoonup u_{\alpha }$ weakly in $L^2(\Omega )$, and this in turn allows us to conclude as in item (i).$\square$

We are now in a position to prove Theorem 5.13.

#### Proof of Theorem  5.13

The proof is subdivided into three steps.

*Step 1.* We prove that if condition *i)* in the statement holds, namely

$$\begin{aligned} TGV_{\hat{\alpha }_0,\hat{\alpha }_1}(u_\eta ,\Omega )- TGV_{\hat{\alpha }_0,\hat{\alpha }_1}(u_c,\Omega ) >0 \end{aligned}$$

for some $\hat{\alpha }\in (0,+\infty )^2$, then there exists $\bar{\alpha }\in (0,+\infty )^2$ such that

5.39 $$\begin{aligned} \begin{aligned} \Vert u_{\bar{\alpha }} - u_c\Vert ^2_{L^2(\Omega )}<\Vert u_\eta - u_c\Vert ^2_{L^2(\Omega )}. \end{aligned} \end{aligned}$$

From the convexity of the *TGV*-seminorm, arguing as in the proof of (3.14), we infer that

$$\begin{aligned} \Vert u_\eta -u_c\Vert _{L^2(\Omega )}^2-\Vert u_{\alpha }-u_c\Vert _{L^2(\Omega )}^2&\leqslant TGV_{\alpha _0,\alpha _1}(u_\alpha ,\Omega )\\  &\quad -TGV_{\hat{\alpha }_0,\hat{\alpha }_1}(u_c,\Omega ) \end{aligned}$$

for every $\alpha \in (0,+\infty )^2$. Choosing $\alpha =\lambda \hat{\alpha }$, and denoting $u_{\lambda (\hat{\alpha })}$ by $u_\lambda$, for simplicity, we find that

$$\begin{aligned} \Vert u_\eta -u_c\Vert _{L^2(\Omega )}^2-\Vert u_{\lambda }-u_c\Vert _{L^2(\Omega )}^2&\leqslant \lambda \left( TGV_{\hat{\alpha }_0,\hat{\alpha }_1}(u_\lambda ,\Omega )\right. \\  &\quad \left. -TGV_{\hat{\alpha }_0,\hat{\alpha }_1}(u_c,\Omega )\right) \end{aligned}$$

for every $\lambda \in (0,+\infty )$. By the proof of case (ii) of Lemma 5.17 and by Corollary 5.16, it follows that, up to (non-relabelled) subsequences, $u_\lambda \rightarrow u_\eta$ strongly in $L^2(\Omega )$ as $\lambda \rightarrow 0$. Fix $\varepsilon >0$; by the lower-semicontinuity of the *TGV*-seminorms with respect to the strong $L^2$-convergence, we conclude that

$$\begin{aligned} TGV_{\hat{\alpha }_0,\hat{\alpha }_1}(u_\lambda ,\Omega )&\geqslant TGV_{\hat{\alpha }_0,\hat{\alpha }_1}(u_\eta ,\Omega )-\varepsilon (TGV_{\hat{\alpha }_0,\hat{\alpha }_1(u_\eta ,\Omega )}\\  &\quad -TGV_{\hat{\alpha }_0,\hat{\alpha }_1}(u_c,\Omega )) \end{aligned}$$

for $\lambda$ small enough. Thus,

$$\begin{aligned}&|u_\eta -u_c\Vert _{L^2(\Omega )}^2-\Vert u_\lambda -u_c\Vert _{L^2(\Omega )}^2 \\&\geqslant \lambda (TGV_{\hat{\alpha }_0,\hat{\alpha }_1}(u_\eta ,\Omega )\\  &\quad -TGV_{\hat{\alpha }_0,\hat{\alpha }_1}(u_c,\Omega ))(1-\varepsilon ) \end{aligned}$$

for $\lambda$ small enough. This implies that there exists $\bar{\lambda }\in (0,+\infty )$ for which

$$\begin{aligned} \Vert u_\eta -u_c\Vert _{L^2(\Omega )}^2>\Vert u_\lambda -u_c\Vert _{L^2(\Omega )}^2. \end{aligned}$$

The preceding estimate yields the thesis by choosing $\bar{\alpha }=\bar{\lambda }(\hat{\alpha }_0,\hat{\alpha }_1)$.

*Step 2.* We prove that if condition *ii)* in the statement holds, (i.e., $\Vert u_\eta - u_c\Vert ^2_{L^2(\Omega )} <\Vert \langle u_\eta \rangle - u_c\Vert ^2_{L^2(\Omega )}$), then there exits $\bar{\alpha }\in (0,+\infty )^2$ such that

5.40 $$\begin{aligned} \begin{aligned} \Vert u_{\bar{\alpha }} - u_c\Vert ^2_{L^2(\Omega )}<\Vert \langle u_\eta \rangle - u_c\Vert ^2_{L^2(\Omega )}. \end{aligned} \end{aligned}$$

In view of Step 1,

$$\begin{aligned} \lim _{\lambda \rightarrow 0}\Vert u_\lambda -u_c\Vert _{L^2(\Omega )}=\Vert u_\eta -u_c\Vert _{L^2(\Omega )}< \Vert \langle u_\eta \rangle -u_c\Vert _{L^2(\Omega )}. \end{aligned}$$

By the proof of case (vi) of Lemma 5.17 and by Corollary 5.16, we obtain the existence of $\bar{\lambda }\in (0,+\infty )$ for which

$$\begin{aligned} \Vert u_{\bar{\lambda }}-u_c\Vert _{L^2(\Omega )}<\Vert \langle u_\eta \rangle -u_c\Vert _{L^2(\Omega )}. \end{aligned}$$

The claim follows by choosing $\bar{\alpha }=\bar{\lambda }(\hat{\alpha }_0,\hat{\alpha }_1)$.

*Step 3.* We conclude the proof by establishing the bounds on the parameters stated in Theorem  5.13. From the lower semicontinuity of $\widehat{J}$, we infer that there exists $\alpha ^*\in [0,+\infty ]^2$ where the minimum value is attained. By Corollary 5.16 and by the previous steps, $\alpha ^*$ satisfies (5.26) and

5.41 $$\begin{aligned} \begin{aligned} \widehat{J} (\alpha ^*)=\min _{\bar{\alpha } \in [0,+\infty ]^2} \widehat{J} (\bar{\alpha }). \end{aligned} \end{aligned}$$

To prove the existence of the lower bound $c_\Omega$, we argue by contradiction. We first assume that there exists a sequence $(\alpha ^*_j)_{j\in \mathbb {N}}\subset (0,+\infty )^2$ such that $\alpha ^*_j\rightarrow 0$ as $j\rightarrow +\infty$, and (5.41) holds for $\alpha ^*=\alpha ^*_j$ for all $j\in \mathbb {N}$. In view of the lower semi-continuity of $\widehat{J}$ on $[0,+\infty ]^2$,

$$\begin{aligned} \begin{aligned} \min _{\bar{\alpha } \in [0,+\infty ]^2} \widehat{J} (\bar{\alpha }) \leqslant \widehat{J} (0)&\leqslant \liminf _{j\rightarrow \infty } \widehat{J} ( \alpha ^*_j) =\min _{\bar{\alpha } \in [0,+\infty ]^2} \widehat{J} (\bar{\alpha }), \end{aligned} \end{aligned}$$

which is false by (5.39). This proves the existence of a constant $\hat{c}_\Omega$ such that $|\alpha ^*|\geqslant \hat{c}_\Omega$ for every minimizer $\alpha ^*$ of $\widehat{J}$. The existence of the constant $c_\Omega$ as in the statement of the theorem follows by observing that the above argument can be repeated by considering sequences $(\alpha _j^*)_{j\in \mathbb {N}}$ for which just one of the entries converges to zero.

The bound from above on $\min \{\alpha ^*_0,\alpha ^*_1\}$ follows directly by Proposition 5.11. In fact, from (5.22), we infer the existence of a constant $C_\Omega$ such that $u_{\alpha ^*}$ is affine if $C_\Omega \Vert u_\eta \Vert _{L^2(\Omega )}<\min \{\alpha ^*_0,\alpha ^*_1\}$. Now, assume by contradiction that there exists a sequence $(\alpha ^*_j)_{j\in \mathbb {N}}\subset (0,+\infty )^2$ such that both entries of $\alpha ^*_j$ blow up to infinity as $j\rightarrow +\infty$, and (5.41) holds for $\alpha ^*=\alpha ^*_j$ for all $j\in \mathbb {N}$. Using, once again, the lower semi-continuity of $\widehat{J}$ on $[0,+\infty ]^2$, we find that

$$\begin{aligned} \begin{aligned} \min _{\bar{\alpha } \in [0,+\infty ]^2} \widehat{J} (\bar{\alpha })&\leqslant \widehat{J} (+\infty ,+\infty ) \leqslant \liminf _{j\rightarrow \infty } \widehat{J} ( \alpha ^*_j) \\  &=\min _{\bar{\alpha } \in [0,+\infty ]^2} \widehat{J} (\bar{\alpha }), \end{aligned} \end{aligned}$$

which is false by Corollary 5.16 and (5.40).$\square$

### The $({\mathscr {L}}\!{\mathscr {S}})_{{TGV-Fid}_\omega }$ Learning Scheme

Given a dyadic square $L\subset Q$ and $\lambda \in (0,\infty )$, we have

$$\begin{aligned} \begin{aligned}&{\text {argmin}}\left\{ \lambda \int _{L}|u_\eta -u|^2\;\text {d}x+ TGV_{1,1}(u,L)\!:\,u\in BV(L)\right\} \\  &\qquad = {\text {argmin}}\left\{ \int _{L}|u_\eta -u|^2\;\text {d}x+ TGV_{\frac{1}{\lambda },\frac{1}{\lambda }}(u,L)\!:\,u\in BV(L)\right\} . \end{aligned} \end{aligned}$$

The analysis in Subsects. 5.1–5.3 applies also to the weighted-fidelity learning scheme and yields Theorem 1.8. As before, the previous existence theorem holds true under any stopping criterion for the refinement of the admissible partitions provided that the training data satisfies suitable conditions. We summarize the situation in the next result, which follows directly by the discussions in the previous subsection, in particular Corollary 5.14.

#### Theorem 5.19

(Equivalence between box constraint and stopping criterion) Consider the learning scheme $({\mathscr {L}}\!{\mathscr {S}})_{{TGV-Fid}_\omega }$ in (1.27). The two following conditions hold:

- If we replace (1.21) by (5.3), then there exists a stopping criterion $(\mathscr {S})$ for the refinement of the admissible partitions as in Definition 1.2.
- Assume that there exists a stopping criterion $(\mathscr {S})$ for the refinement of the admissible partitions as in Definition 1.2 such that the training data satisfies for all , with $\bar{\mathscr {P}}$ as in Definition 1.2, the conditions
  (i) $$TGV_{\alpha _0, \alpha _1}(u_c,L) < TGV_{\alpha _0, \alpha _1}(u_\eta ,L)$$,
  (ii) $$\displaystyle \Vert u_\eta - u_c\Vert ^2_{L^2(L)} <\Vert \langle u_\eta \rangle _L - u_c\Vert ^2_{L^2(L)} $$.
  Then, there exist $c_0, c_1\in \mathbb {R}^+$ such that the optimal solution $u^*$ provided by $({\mathscr {L}}\!{\mathscr {S}})_{{TGV-Fid}_\omega }$ with $\mathscr {P}$ replaced by $\bar{\mathscr {P}}$ coincides with the optimal solution $u^*$ provided by $({\mathscr {L}}\!{\mathscr {S}})_{{TGV-Fid}_\omega }$ with (1.21) replaced by (5.3).

## Numerical Treatment and Comparison of the Learning Schemes $({\mathscr {L}}\!{\mathscr {S}})_{{TV\!}_{\omega }}$, $({\mathscr {L}}\!{\mathscr {S}})_{{TV\!}_{\omega _\epsilon }}$, $({\mathscr {L}}\!{\mathscr {S}})_{{TV-Fid}_\omega }$, and $({\mathscr {L}}\!{\mathscr {S}})_{{TGV-Fid}_\omega }$

### Common Numerical Framework for all Schemes

The focus of our article is on the use of space-dependent weights and, from the numerical point of view, our schemes require addressing weights that are piecewise constant on dyadic partitions. This stands in contrast to most previous approaches for optimizing space-dependent parameters, which in most cases hinge on $H^1$-type penalizations of the weights, as done in [25, 45] for TV, [43] for TGV and [55] for some more general convex regularizers. The piecewise constant setting makes it possible to work in a modular fashion, building upon any numerical methods that are able to compute solutions to denoising with a weight (Level 2) and finding constant optimal regularization parameters (Level 3).

**Fig. 2:**
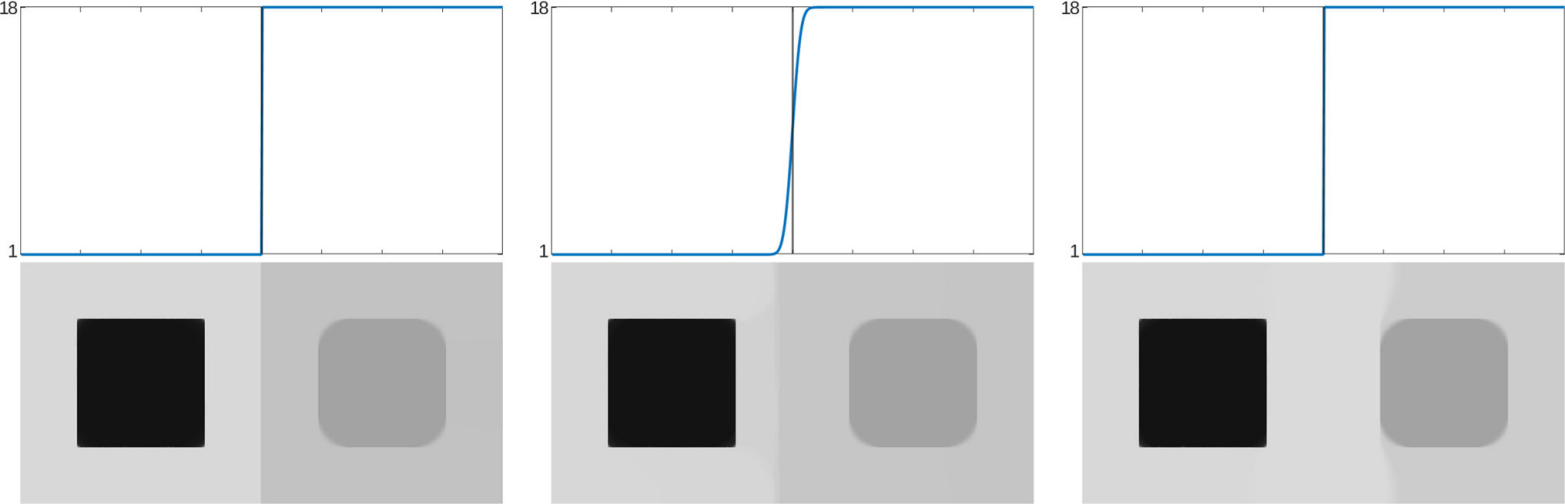
Oversmoothed denoising with a sharp change of weight and schemes corresponding to Level 2 of $({\mathscr {L}}\!{\mathscr {S}})_{{TV\!}_{\omega }}$, $({\mathscr {L}}\!{\mathscr {S}})_{{TV\!}_{\omega _\epsilon }}$, and $({\mathscr {L}}\!{\mathscr {S}})_{{TV-Fid}_\omega }$, from left to right. Top row: weights $\omega (x)=\omega (x_1)$. Bottom row: results with each denoising scheme and the corresponding (not optimal) weight

**Fig. 3:**
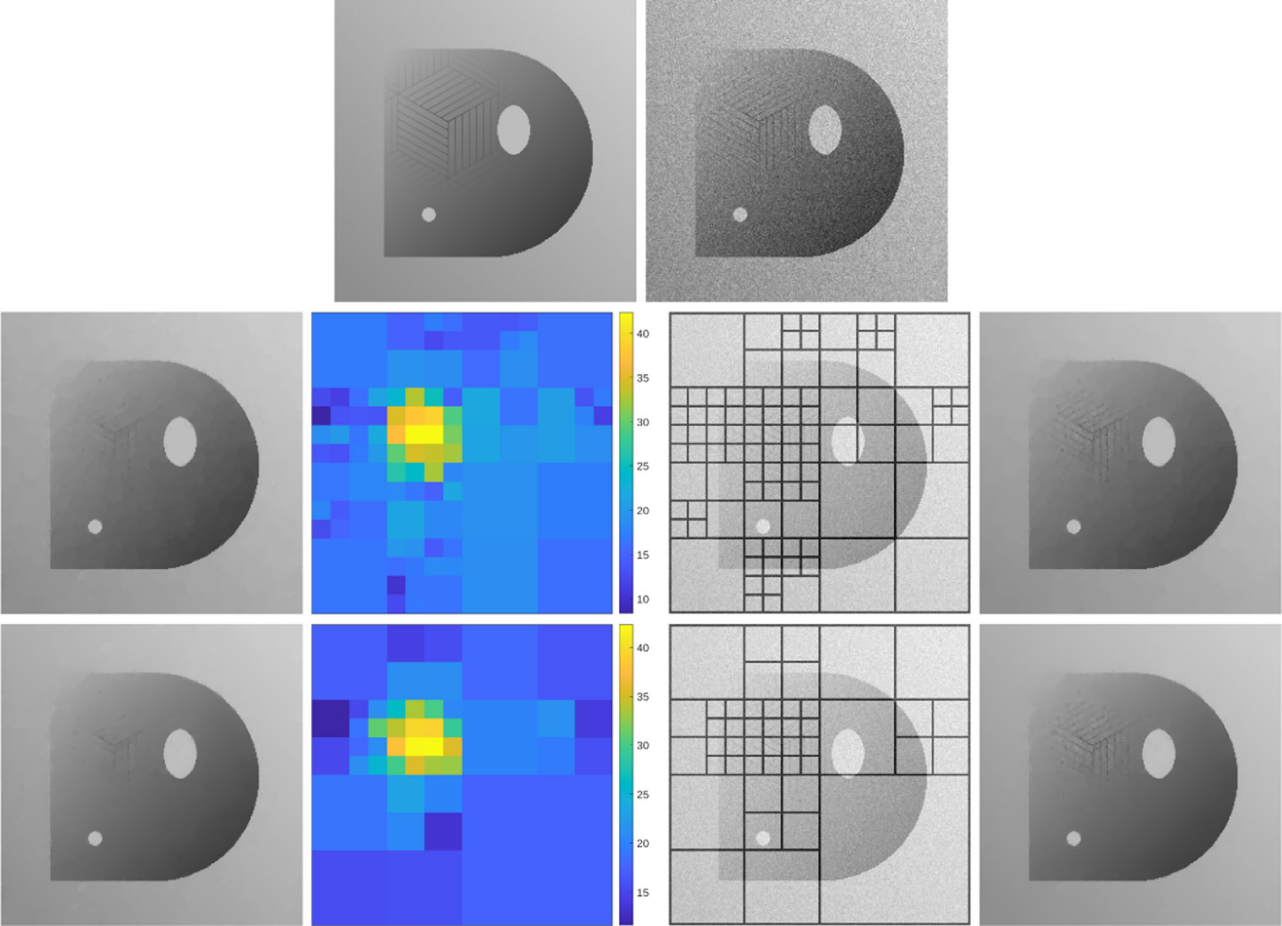
Synthetic example. Top row: Clean and noisy images $u_c, u_\eta$. Middle row, left to right: TV result with global parameter, partition and spatially-dependent $\lambda$ arising from Algorithm 2, and corresponding result with weighted fidelity. Bottom row: TGV results, same order as in the middle row and with $\alpha _0=1, \alpha _1 = 10$

**Fig. 4:**
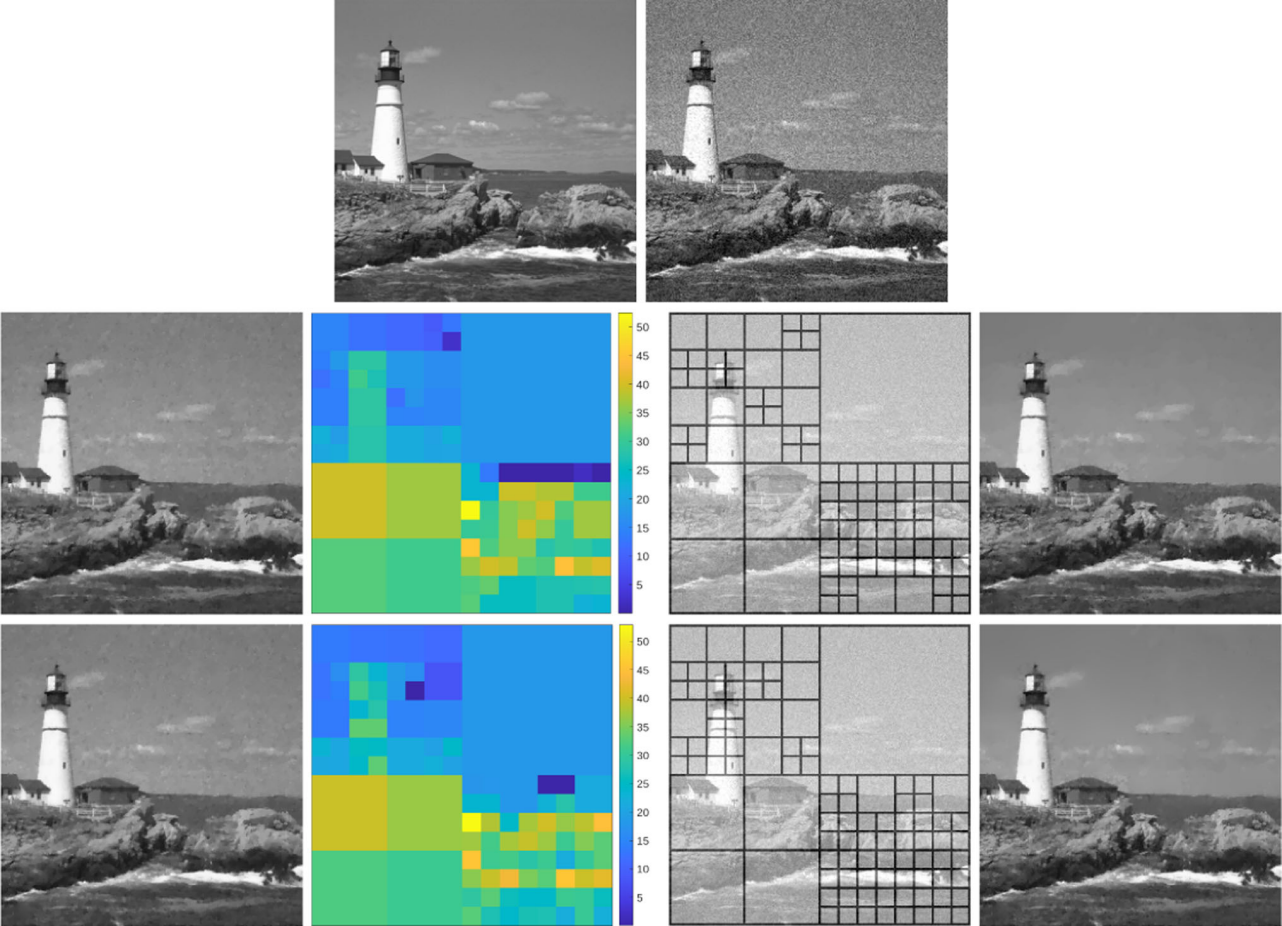
Lighthouse example. Top row: Clean and noisy images $u_c, u_\eta$. Middle row, left to right: TV result with global parameter, partition and spatially-dependent $\lambda$ arising from Algorithm 2, and corresponding result with weighted fidelity. Bottom row: TGV results, same order as in the middle row and with $\alpha _0=1, \alpha _1 = 2$

**Fig. 5:**
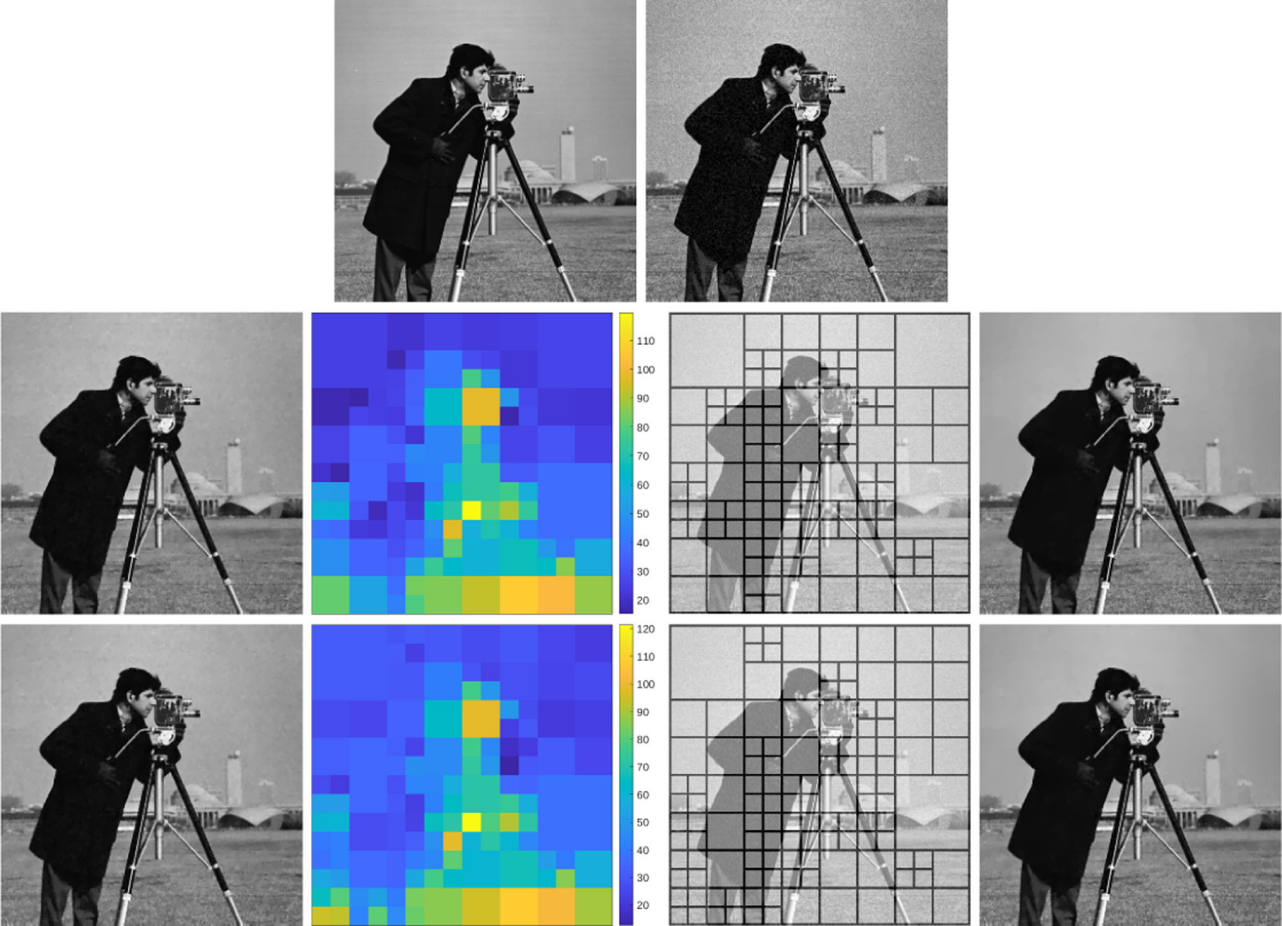
Cameraman example. Top row: Clean and noisy images $u_c, u_\eta$. Middle row, left to right: TV result with global parameter, partition and spatially-dependent $\lambda$ arising from Algorithm 2, and corresponding result with weighted fidelity. Bottom row: TGV results, same order as in the middle row and with $\alpha _0=1, \alpha _1 = 10$

In our numerical examples, we have used a basic first-order finite difference discretization of the gradient and symmetrized gradient, on the regular grid arising from the discrete input images. For solving *TV* regularized denoising, either with constant or varying weights, we have opted for the standard primal-dual hybrid gradient (PDHG) descent scheme of [19]. The optimization for optimal constant parameters $\alpha$ of Level 3 is done with the ‘piggyback’ version of the same algorithm, which has been proposed in [20] to learn finite difference discretizations of *TV* with a high degree of isotropy, and further analyzed under smoothness assumptions on the energies in [6]. Essentially, it consists in evolving an adjoint state along with the main variables, to keep track of the sensitivity of the solution with respect to parameters. We remark that such sensitivity analysis in principle requires not just first but second derivatives of the lower-level cost functions involved, in our case TV or TGV denoising involving weighted $\ell ^1$ norms and their Fenchel conjugates, which are only componentwise piecewise smooth. In any case, as already observed in [20, Appendix A], we do achieve an adequate performance in practice. It is worth mentioning that other methods to handle the bilevel optimization problems of Level 3 in a nonsmooth setting have been introduced in [8, 32, 36]. One could also use these in our subdivision scheme within Algorithm 2, and in fact the authors of the cited papers optimize for adaptive weights on regular dyadic grids refined uniformly. In contrast, our focus here is on the adaptive subdivision scheme.

These PDHG methods are based on considering the discrete optimization problems

$$\begin{aligned} \min _{x \in \mathcal {X}}\, \mathcal {G}(x) + \mathcal {F}(Ky) \end{aligned}$$

through their corresponding saddle point formulation

$$\begin{aligned} \max _{y \in \mathcal {Y}} \min _{x \in \mathcal {X}}\, \langle y, Kx \rangle _{\ell ^2} + \mathcal {G}(x) - \mathcal {F}^*(y), \end{aligned}$$

with $\mathcal {G}$ representing the differentiable fidelity term and $\mathcal {F}^*$ being the projection onto a convex set, arising as the Fenchel conjugate of an $\ell ^1$-type norm. Denoting by $W=\mathbb {R}^{nm}$ the space of discrete scalar-valued functions, these read in the *TV* case as

$$\begin{aligned}  &   \mathcal {X}=W,\ \mathcal {Y}=W^2,\ K=\nabla ,\ \mathcal {G}(u)=\lambda \sum _{ij} \big ( u^{ij} - u^{ij}_\eta \big )^2,\\    &   \text { and }\mathcal {F}^*(p){=}\mathcal {I}_{Q_{TV}} \text { with } \\    &   Q_{TV} {=} \big \{ p \in \mathcal {Y}\,|\, (p^{ij}_1)^2 + (p^{ij}_2)^2 \leqslant \alpha \text { for all }i,j\big \}. \end{aligned}$$

For the TGV case, following the approach used in [7] and [9], we have used

$$\begin{aligned}  &   \mathcal {X}=W \times W^2,\ \mathcal {Y}=W^2 \times W^3,\ K=\begin{pmatrix} \nabla u &  - \text {Id} \\ 0 &  \mathcal {E}\end{pmatrix},\\    &   \mathcal {G}(u,v){=}\lambda \sum _{ij} \big ( u^{ij} {-} u^{ij}_\eta \big )^2,\ \text { and } \mathcal {F}^*(p){s=}\mathcal {I}_{Q_{TGV}}, \text { where } \\    &   Q_{TGV} = \big \{ (p,q) \in \mathcal {Y}\,|\, (p^{ij}_1)^2 + (p^{ij}_2)^2 \leqslant \alpha _0,\\  &   \quad \qquad \qquad (q^{ij}_{11})^2 + 2(q^{ij}_{12})^2 + (q^{ij}_{22})^2 \leqslant \alpha _1\\  &   \quad \qquad \qquad \text { for all }i,j\big \}. \end{aligned}$$

With this notation and denoting the subgradient by $\partial$, the PDHG algorithm [19, Algorithm 1] can be written as

6.1 $$\begin{aligned} {\left\{ \begin{array}{ll}y^{k+1}= (\text {Id} + \sigma \partial \mathcal {F}^*)^{-1}(y^k + \sigma K \bar{x}^{k}),\\ x^{k+1}=(\text {Id} + \tau \partial \mathcal {G})^{-1}(x^k - \tau K^*y^{k+1}),\\ \bar{x}^{k+1} = x^{k+1} + \theta ( x^{k+1} - x^k ),\end{array}\right. } \end{aligned}$$

**Fig. 6:**
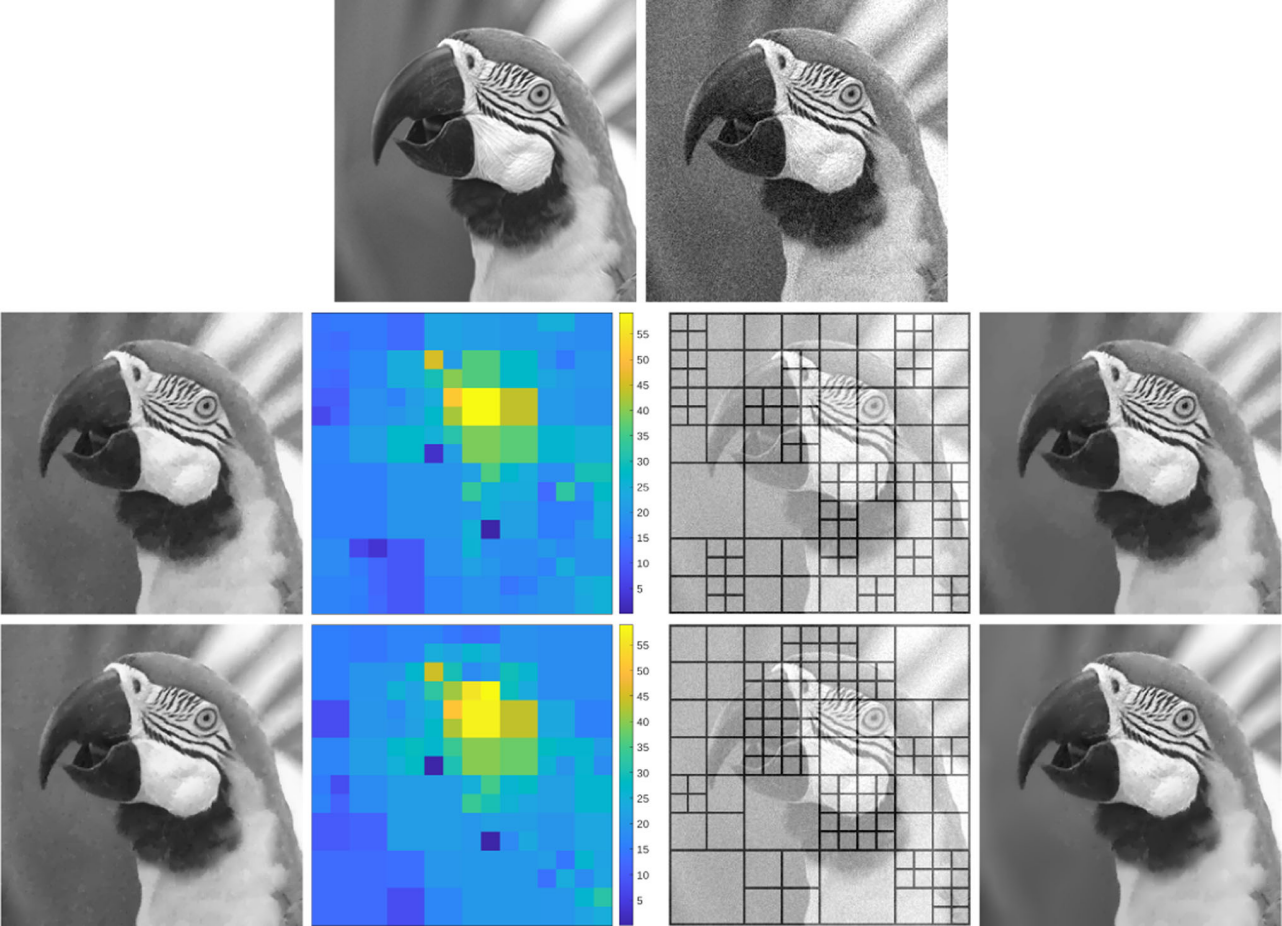
Parrot example. Top row: Clean and noisy images $u_c, u_\eta$. Middle row, left to right: TV result with global parameter, partition and spatially-dependent $\lambda$ arising from Algorithm 2, and corresponding result with weighted fidelity. Bottom row: TGV results, same order as in the middle row and with $\alpha _0=1, \alpha _1 = 2$

where the descent parameters satisfy $\sigma \tau \Vert K\Vert \leqslant 1$. In the *TV* case, this operator norm of $\nabla$ can be bounded by $\sqrt{8}$ (cf. [17, Theorem 3.1]), while in the *TGV* case, we have $\Vert K\Vert ^2 \leqslant (17+\sqrt{33})/2$ (cf. [7, Section 3.2]). The piggyback algorithm of [6, 20] introduces one adjoint variable for each primal and dual variable above (denoted by $X \in \mathcal {X}$, $Y \in \mathcal {Y}$, $U \in W$, $P \in W^2$, $Q \in W^3$) and performs the same kind of updates also on these new variables to optimize the values of a loss function *L*, resulting in

6.2 $$\begin{aligned} {\left\{ \begin{array}{ll}Y^{k+1}= D{{\,\textrm{prox}\,}}_{\sigma \mathcal {F}^*}(y^k + \sigma K \bar{x}^{k})\\ \cdot \big [Y^k + \sigma K \big (\bar{X}^{k}+ D_x L(x^k, y^k) \big )\big ],\\ X^{k+1}=D{{\,\textrm{prox}\,}}_{\tau \mathcal {G}}(x^k - \tau K^*y^{k+1}) \\ \cdot \big [X^k- \tau K^*\big (Y^{k+1} {+} D_y L(x^k, y^k) \big )\big ],\\ \bar{X}^{k+1} {=} X^{k+1} {+} \theta ( X^{k+1} - X^k ),\end{array}\right. }\nonumber \\ \end{aligned}$$

where ${{\,\textrm{prox}\,}}_{\tau \mathcal {G}}=(\text {Id} + \tau \partial \mathcal {G})^{-1}$ and ${{\,\textrm{prox}\,}}_{\sigma F^*}=(\text {Id} + \tau \partial F^*)^{-1}$ as appearing in (6.1); the latter corresponds to a projection onto $Q_{TV}$ or $Q_{TGV}$ which, as already remarked, is not differentiable on the boundary of these sets.

In our case, we optimize the squared $L^2$ distance to $u_c$ by varying the fidelity parameter $\lambda = 1/\alpha$, so that

6.3 $$\begin{aligned} L(u)  &   = \frac{1}{2} \sum _{ij} (u^{ij} - u^{ij}_c)^2\ \text { and }\ D_\lambda \mathcal {L}(\lambda ) \nonumber \\    &   = \lambda \sum _{ij} \hat{U}^{ij} (\hat{u}^{ij} - u^{ij}_\eta )\ \text { for }\ \mathcal {L}(\lambda ) = L(\hat{u}(\lambda )), \end{aligned}$$

where $\hat{u}$, $\hat{U}$ are the optimal image variable and corresponding adjoint obtained after convergence of (6.1) and (6.2). We have then used the derivative $D_\lambda \mathcal {L}$ to update $\lambda$ with gradient descent steps. We have chosen to not use line search, since with the piggyback algorithm evaluations of energy and of gradient for the solution of the lower level problem require a comparable amount of computational effort, that is, either performing (6.1) alone or together with (6.2) for the same number of lower level steps. We summarize this basic approach in Algorithm 1.

**Algorithm 1:**
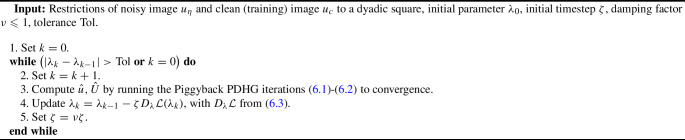
Numerical approach to Level 3 of $(\mathscr {L}\!\mathscr {S})_{{TV-Fid}_\omega }$ and $(\mathscr {L}\!\mathscr {S})_{{TGV-Fid}_\omega }$

**Table 1:** PSNR and SSIM values for the examples of Figs. 3, 4, 5, and 6

	Noisy	*TV* global	*TV* adaptive	*TGV* global	*TGV* adaptive
Synthetic	26.05, 0.349	38.74, 0.946	39.41, 0.957	39.02, 0.949	39.80, 0.961
Lighthouse	24.64, 0.496	30.42, 0.853	30.82, 0.886	30.44, 0.855	30.90, 0.890
Cameraman	28.40, 0.642	32.86, 0.893	33.54, 0.925	32.86, 0.893	33.56, 0.925
Parrot	24.67, 0.447	31.86, 0.880	32.37, 0.898	32.10, 0.889	32.72, 0.909

It is worth noting that we are optimizing only on the parameter $\lambda$ in front of the fidelity term. For the TV case and since this algorithm is applied to Level 3 with constant parameters, only the balance between the two energy terms is relevant and finding an optimal $\lambda _L$ is equivalent to finding an optimal $\alpha _L = 1/\lambda _L$, which can then be assembled over all *L* into a weight $\omega$ for Level 2 of either $({\mathscr {L}}\!{\mathscr {S}})_{{TV-Fid}_\omega }$ or $({\mathscr {L}}\!{\mathscr {S}})_{{TV}_\omega }$. In the TGV setting, optimizing only over one parameter imposes a restriction, but we have chosen to do so to keep the simple approach of Algorithm 1 and avoid more complicated behaviors of the costs when varying both $\alpha _0$ and $\alpha _1$ (or, equivalently, $\lambda$ together with either $\alpha _0$ and $\alpha _1$).

### Effect of parameter discontinuities in Level 2 of $({\mathscr {L}}\!{\mathscr {S}})_{{TV\!}_{\omega }}$, $({\mathscr {L}}\!{\mathscr {S}})_{{TV\!}_{\omega _\epsilon }}$ and $({\mathscr {L}}\!{\mathscr {S}})_{{TV-Fid}_\omega }$

In Fig. 2, we present an example using large regularization parameters and a symmetric input image to demonstrate the effect of parameter discontinuities in Level 2 of the schemes $({\mathscr {L}}\!{\mathscr {S}})_{{TV\!}_{\omega }}$, $({\mathscr {L}}\!{\mathscr {S}})_{{TV\!}_{\omega _\epsilon }}$ and $({\mathscr {L}}\!{\mathscr {S}})_{{TV-Fid}_\omega }$. In the weighted-*TV* result, a jump in the weight results in a spurious discontinuity in the resulting image. Mollifying the weight smooths the transition slightly, and it shifts it to the side with lower weight. Using a weighted fidelity term does not introduce discontinuities besides those present in the input, but still creates visible artifacts near them.

**Algorithm 2:**
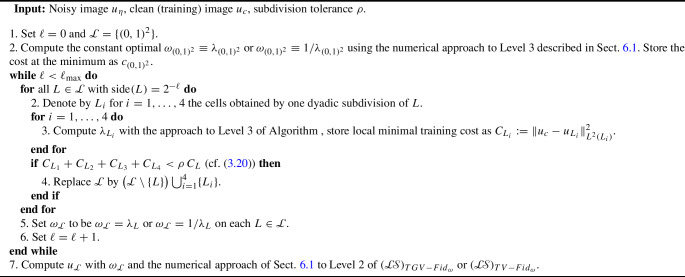
Numerical approach to Level 1

### Dyadic Subdivision Approach To Level 1

In Algorithm 2, we summarize our approach to numerically treat Level 1. We remark that in comparison with the original formulations $({\mathscr {L}}\!{\mathscr {S}})_{{TV\!}_{\omega }}$ and $({\mathscr {L}}\!{\mathscr {S}})_{{TGV\!}_{\omega }}$ as formulated in the introduction, we do not search the entire space of partitions (which would be numerically intractable) and instead work by subdivision as in Example 3.13. This means that for any given cell *L*, we make a local decision whether to subdivide it or not, based on the training costs arising from it before and after subdividing it in four new cells. When performing this subdivision, the parameter from the original cell is used as initialization for the optimization on the newly created ones. Even though this approach strongly restricts the number of possible partitions considered, it still manages to achieve reasonable performance in practice. On a heuristic level, this indicates that if splitting one dyadic square once to add more detail on the parameter does not lead to better performance, then in most cases it is also not advantageous to consider further finer subdivisions of the same square.

### Numerical Examples with the Complete Schemes $({\mathscr {L}}\!{\mathscr {S}})_{{TV-Fid}_\omega }$ and $({\mathscr {L}}\!{\mathscr {S}})_{{TGV-Fid}_{\omega }}$

In Figs. 3, 4, 5, and 6, we present some illustrative examples resulting from the application of Algorithm 2 with $\ell _{\max } = 4$ to several images, for both *TV* and *TGV* regularization and optimizing for one adaptive parameter in the fidelity term, which is also shown along with the partitions overlaid on the noisy input images. In these, we generally see that the adapted fidelity parameter $\lambda$ is higher in areas with finer details. Peak signal to noise ratios and SSIM values for each case are summarized in Table 1. In all cases, *TGV* with adaptive fidelity produces the best results by these metrics, but there are several instances where the gains are very marginal or there are even ties with the corresponding adaptive *TV* results. Nevertheless, it may be argued that even in these cases the *TGV* results are more visually appealing due to reduced staircasing.

For the simple example of Fig. 3, some more direct observations can be made. In it, we see that the spatially adaptive results manage to better preserve the fine structures inside the main object, while TGV greatly diminishes staircasing in regions where the original image is nearly linear. Observe that unlike the fine structures, the boundaries of the main object consisting of a sharp discontinuity along an interface with low curvature do not necessarily force further subdivision, as expected for *TV* or *TGV* regularization.

The synthetic image used in Fig. 3 was created by the authors for this article. The lighthouse and parrot examples in Figs. 4 and 6 have been cropped and converted to grayscale from images in the Kodak Lossless Image Suite. The cameraman image of Fig. 5 is very widely used, but to our knowledge its origin is not quite clear.
